# The Reactions of 6-(Hydroxymethyl)-2,2-dimethyl-1-azaspiro[4.4]nonanes with Methanesulfonyl Chloride or PPh_3_-CBr_4_

**DOI:** 10.3390/molecules26196000

**Published:** 2021-10-02

**Authors:** Yulia V. Khoroshunova, Denis A. Morozov, Andrey I. Taratayko, Sergey A. Dobrynin, Ilia V. Eltsov, Tatyana V. Rybalova, Yulia S. Sotnikova, Dmitriy N. Polovyanenko, Nargiz B. Asanbaeva, Igor A. Kirilyuk

**Affiliations:** 1 N.N. Vorozhtsov Institute of Organic Chemistry SB RAS, Academician Lavrentiev Ave. 9, 630090 Novosibirsk, Russia; dmorozov@nioch.nsc.ru (D.A.M.); taratayk@nioch.nsc.ru (A.I.T.); s.a.dobrynin@gmail.com (S.A.D.); rybalova@nioch.nsc.ru (T.V.R.); sotnikova@nioch.nsc.ru (Y.S.S.); dpolo@nioch.nsc.ru (D.N.P.); nargiz-asan@mail.ru (N.B.A.); kirilyuk@nioch.nsc.ru (I.A.K.); 2 Department of Natural Sciences, Novosibirsk State University, Pirogova Str. 1, 630090 Novosibirsk, Russia; eiv@fen.nsu.ru

**Keywords:** appel reaction, alkylation, nitroxide, azepane, Hofmann elimination

## Abstract

Activation of a hydroxyl group towards nucleophilic substitution via reaction with methanesulfonyl chloride or PPh_3_-CBr_4_ system is a commonly used pathway to various functional derivatives. The reactions of (5*R*(*S*),6*R*(*S*))-1-X-6-(hydroxymethyl)-2,2-dimethyl- 1-azaspiro[4.4]nonanes 1**a**–**d** (X = O·; H; OBn, OBz) with MsCl/NR_3_ or PPh_3_-CBr_4_ were studied. Depending on substituent X, the reaction afforded hexahydro-1*H*,6*H*-cyclopenta[*c*]pyrrolo[1,2-*b*]isoxazole (**2**) (for X = O), a mixture of **2** and octahydrocyclopenta[*c*]azepines (**4**–**6**) (for X = OBn, OBz), or perhydro-cyclopenta[2,3]azeto[1,2-*a*]pyrrol (3) (for X = H) derivatives. Alkylation of the latter with MeI with subsequent Hofmann elimination afforded 2,3,3-trimethyl-1,2,3,4,5,7,8,8a-octahydrocyclopenta[*c*]azepine with 56% yield.

## 1. Introduction

Activation of a hydroxyl group towards nucleophilic substitution via reaction with methanesulfonyl chloride or PPh_3_-CBr_4_ system is a commonly used pathway to various functional derivatives [1,2,3]. Recently, we reported on simple synthesis of (5*R*(*S*),6*R*(*S*))-2,2-dimethyl-6-(hydroxymethyl)-1-azaspiro[4.4]nonane-1-oxyl **1a** from commercially available 5,5-dimethyl-1-pyrroline *N*-oxide (DMPO) [4]. The above nitroxide and its analogs were prepared as single enantiomeric pair with hydroxymethyl group close to the nitroxide one. Close proximity of hydroxy and nitroxide groups make these compounds potential precursors of rigid spin labels, which might allow precise distance measurements via site-directed spin labeling—PELDOR technique [5,6]. Synthesis of these spin labels would require replacement of the hydroxyl group to methanethiosulfonate or maleimido moiety, and Appel reaction or treatment with methanesulfonyl chloride seems to be a proper step towards this direction.

**Scheme 1:**
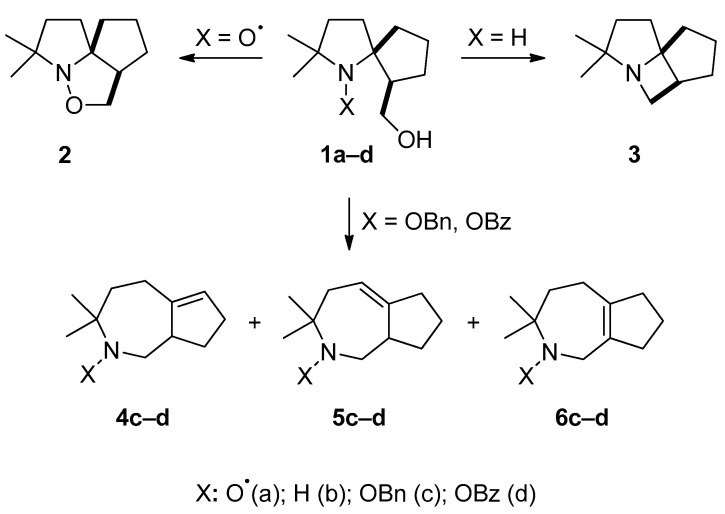
The reactions of 6-(hydroxymethyl)-2,2-dimethyl-1-azaspiro[4.4]nonanes **1a**–**d** with MsCl/NR_3_ and PPh_3_-CBr_4_.

Here, we describe the reactions of **1a** and its diamagnetic analogs (5*R*(*S*),6*R*(*S*))-1-X-6-(hydroxymethyl)-2,2-dimethyl-1-azaspiro[4.4]nonanes **1b**–**d** (X = OBn; OBz; H) with MsCl/NR_3_ or PPh_3_-CBr_4_. Surprisingly, none of these reactions allowed us to isolate expected bromomethyl- or mesyloxymethyl-substituted compounds. The product structure was dependent on substituent X in the position 1 of the pyrrolidine ring. For nitroxide (X = O**·**) hexahydro-1*H*,6*H*-cyclopenta[*c*]pyrrolo[1,2-*b*]isoxazole (**2**) was isolated, unsubstituted amine (X = H) afforded unknown perhydro-cyclopenta[2,3]azeto[1,2-*a*]pyrrol (**3**), while alkoxy- or acyloxyamines (X = OBn, OBz) afforded unknown octahydrocyclopenta[*c*]azepine derivatives (**4c**–**d**, **5c**–**d** and **6c**–**d**) (Scheme 1). To the best of our knowledge, the ring junction similar to perhydrocyclopenta[2,3]azeto[1,2-a]pyrrol was only observed in *cis*,*cis*,*cis*,*cis*-[5.5.5.4]-1-azafenestrane system [7]. Azepanes are of continuous interest for preparation of bioactive compounds. This scaffold is present in numerous pharmaceuticals [8,9,10,11,12,13,14,15,16].

## 2. Results and Discussion

**Scheme 2:**
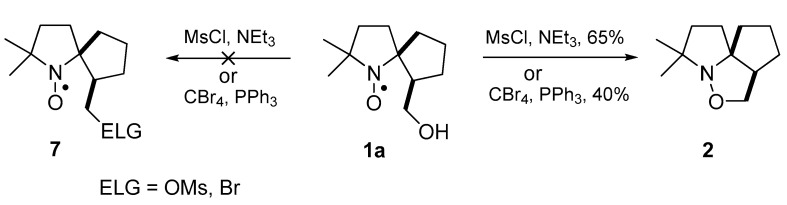
The reactions of **1a** with MsCl/NEt_3_ and PPh_3_-CBr_4_.

Both mesylation and the Appel reaction were successfully used in nitroxide chemistry, however, the Appel reaction often gives low to moderate yields of brominated nitroxides [17], while the yields of OMs derivatives are high [18,19,20]. The reactions of (5*R*(*S*),6*R*(*S*))-6-(hydroxymethyl)-2,2-dimethyl-1-azaspiro[4.4]nonan-1-oxyl (**1a**) with MsCl/NEt_3_ or with PPh_3_-CBr_4_ afforded no expected nitroxides **7**. The reaction mixtures turned brown with significant tarring and the earlier described 3,3-dimethyloctahydrocyclopenta[*c*]pyrrolo[1,2-*b*]isoxazole **2 [4]** was isolated from both reaction mixtures with a moderate yield (Scheme 2).

**Scheme 3:**
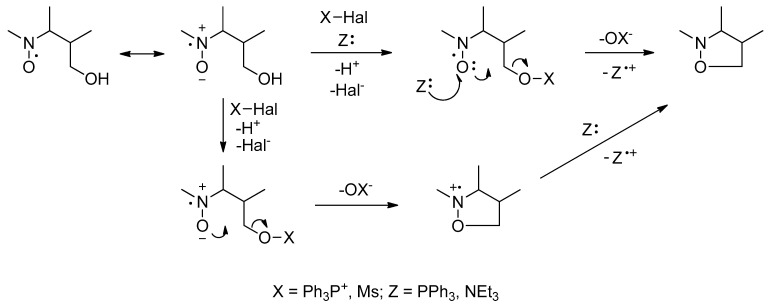
The possible mechanism of the formation of **2** in the reactions of **1a** with MsCl/NEt_3_ and PPh_3_-CBr_4_.

Unusual behavior of **1a** can only be explained by close proximity of hydroxymethyl and nitroxide groups in this molecule (ca. 3 Å distance). The possible mechanism of the formation of **2** is shown in Scheme 3. The reaction must anyway include one-electron reduction and cyclization via nucleophilic substitution steps. It is not clear whether activation of nitroxide oxygen to nucleophilic attack with one-electron transfer is required for a cyclization step or cyclic radical-cation can be formed spontaneously first and then undergoes one-electron reduction. Tertiary amines and phosphines are known to act as single-electron donors in various reactions, see for example [21,22].

**Scheme 4:**
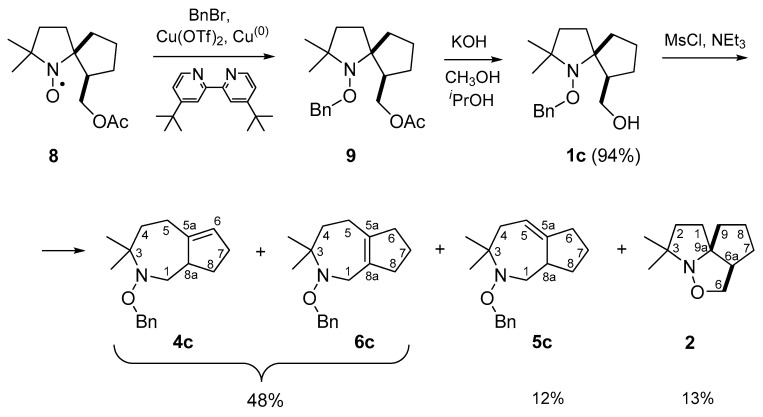
Synthesis of alkoxyamine **1c** and its reaction with MsCl/NEt_3_. The yield of **4c** (pure fraction isolated using preparative HPLC) was 25%. The ratio in the reaction mixture (NMR): **4c**/**5c**/**6c**/**2** = 12.63/3.28/1/9.42.

Conversion into alkoxy groups is a convenient way of the protection of nitroxide groups from reactions onto the oxygen atom [23]. We have previously reported on the synthesis of alkoxyamine **9** via treatment of corresponding nitroxide **8** with benzyl bromide in the presence of in situ formed Cu (I) using Matyjaszewski’s procedure [4]. Alkaline hydrolysis of **9** afforded **1c** with nearly quantitative yield, and subsequent treatment with MsCl in the presence of a base gave a mixture of products (Scheme 4).

**Scheme 5:**
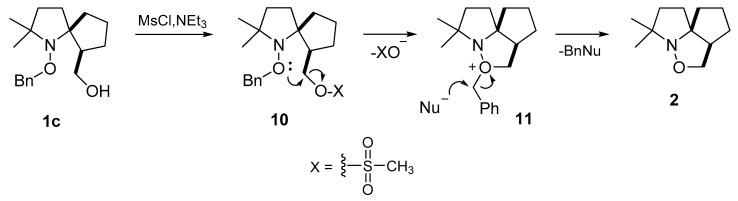
The possible mechanism of product **2** formation.

Column chromatography allowed us to isolate two individual compounds, and in addition, an inseparable mixture of two compounds was collected. HPLC showed that the mixture was composed of two compounds (Figure S62). The main component was isolated from the mixture using preparative HPLC. Non-polar character of the products allowed us to discard quaternary alkoxyammonium salt structure, which could be a result of intramolecular alkylation on alkoxyamine nitrogen in analogy to literature [24,25,26]. IR and NMR spectra allowed us to identify one of the isolated products as **2** (preparative yield about 13%)**.** The formation of product **2** in this process may occur via intramolecular alkylation onto O-alkoxyamine atom of intermediate **10** to give cation **11** with subsequent nucleophilic substitution (Scheme 5).

HRMS spectra of the other two isolated compounds showed the same molecular ions ([M]^+^ ca. 271, see Experimental part) with a formula C_18_H_25_NO, correspondent to products of the formal elimination of water from **1c**. The NMR spectra confirmed that both isomers retained benzyloxy moieties. The multiplets at 5.2–5.4 ppm in ^1^H NMR spectra and signals at 137–138 and 148–150 ppm in ^13^C NMR indicated the formation of a trisubstituted ethylene moiety. Assignment of the structure of the isomers was performed on the basis of ^1^H-^1^H COSY, ^1^H-^13^C HMBC and ^1^H-^13^C HSQC NMR spectra (see Figures S42–S47 and Appendix A). At room temperature, some signals in the spectra of **5c** were broadened, presumably due to slow conformational changes, therefore, the spectra acquired at 333 K with more narrow lines were used for assignments. Analysis of interactions in the 2D spectra allowed us to conclude that compounds **4c** and **5c** have the same octahydrocyclopenta[*c*]azepine backbone, differing only in the position of the double >C=CH bond, which is located in 5- or 7-membered ring.

The NMR analysis of the fraction containing **4c** (major isomer) obtained via column chromatography showed the extra signals. These signals may belong to a third isomer, **6c**, in which a fully substituted double bond is located between the rings. Indeed, in the ^1^H NMR spectrum of this fraction recorded at 333 K (Figure S20) a singlet at 1.18 (s, 6H), multiplet signals of five methylene groups in the region of 1.64–2.40 ppm partially overlapped with the signals of the major isomer **4c**, the singlet signal of the methylene group at the heteroatom (3.51 ppm) and the signal of the benzyl methylene group at 4.63 ppm were observed. In the ^13^C NMR spectrum (Figure S22), two signals in the low-field region at 138.17 and 138.27 ppm were observed, which correspond to the carbons of tetrasubstituted C=C bond. The ratio of the products was determined using the NMR spectrum of the reaction mixture: **4c**/**5c**/**6c**/**2** = 12.63/3.28/1/9.42.

**Scheme 6:**
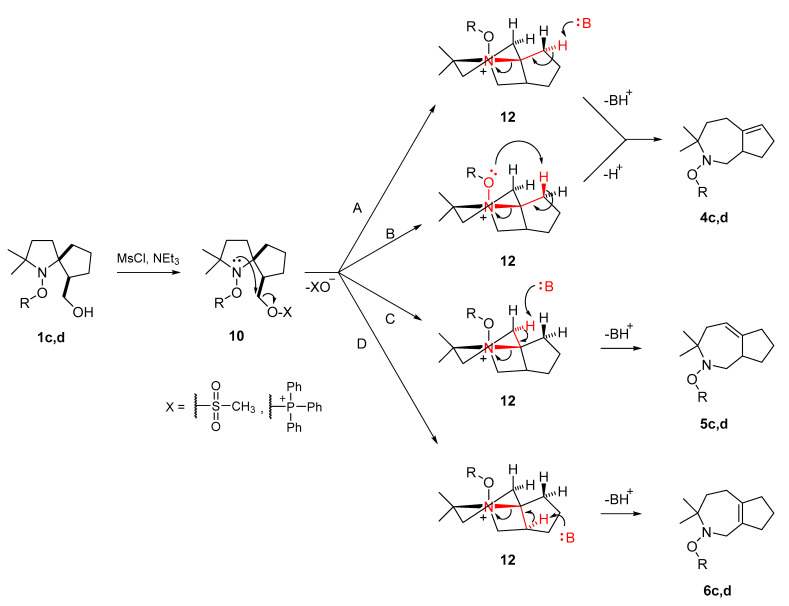
The possible mechanisms of the formation of products **4c**–**d, 5c**–**d,** and **6c**–**d**.

The possible mechanism of **4c**, **5c**, and **6c** formation is likely to imply intramolecular alkylation to give *N*-benzyloxyammonium salt **12** with subsequent Hofmann-type elimination, however, one cannot exclude a contribution of Cope-type elimination via 5-membered transition state with coordination of alkoxyamine oxygen in **1c** with the most closely located hydrogen atom (methylene hydrogen of cyclopentane ring, ca. 2.5 Å distance) (Scheme 6) [27]. This may account for predominant formation of isomer **4c**. Some examples of the conversion of 1-methyl-1-azoniabicyclo[3.2.0]heptanes into azepane derivatives are known [9,28], but in these examples ring opening occurred via nucleophilic substitution, not via elimination.

Under the Appel reaction conditions, **1c** was converted into the same products **4c**, **5c**, **6c** and **2** in the ratio of 3.89/1.35/1/4.61, respectively, according to NMR. Total preparative yield of the isomers **4c**, **5c,** and **6c** was 34%.

**Scheme 7:**
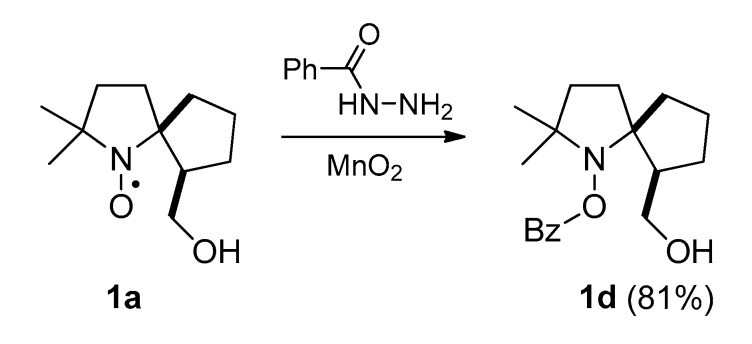
Synthesis of *N*-benzoyloxy derivative **1d**.

With the replacement of benzyl group in **1c** with electron-withdrawing benzoyl, one could make the N-O-R moiety less prone to intramolecular alkylation. To prepare *N*-benzoyloxy derivative **1d**, the nitroxide **1a** was treated with benzhydrazide in the presence of excess MnO_2_ [29]. This method allowed us to avoid the acylation of hydroxymethyl group and afforded **1d** with the yield of 81% as colorless oil not susceptible to oxidation to nitroxide (Scheme 7). The presence of free hydroxy group in **1d** was confirmed with IR absorption band at 3452 cm^−1^ and a broad singlet (1H) at 5.29 ppm in the ^1^H NMR spectrum.

**Scheme 8:**
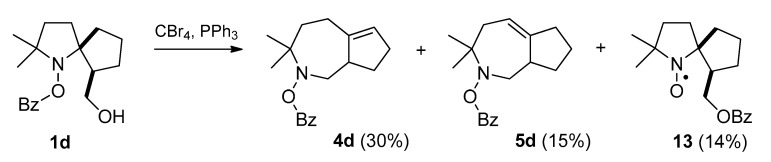
Reaction of **1d** with PPh_3_-CBr_4_.

Reaction of **1d** with PPh_3_-CBr_4_ afforded a mixture, from which three compounds were isolated (Scheme 8).

**Scheme 9:**
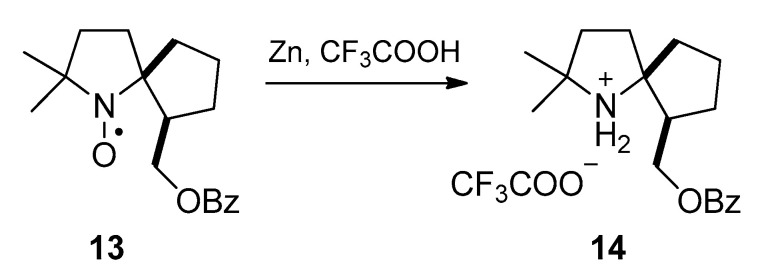
Reduction of nitroxide **13** with Zn in CF_3_COOH.

One of the isolated compounds, a yellow crystalline solid, showed intense triplet in EPR spectrum with *a*_N_ = 1.49 mT (Figure S57). HRMS with a molecular ion, [M^+^] = 302.1752, corresponding to the formula C_18_H_24_NO_3_, IR spectrum with strong absorption band at 1716 cm^−1^ and no absorption above 3100 cm^−1^ favored the structure **13**, which could result from transesterification and oxidation. The structure was confirmed with ^1^H NMR spectrum recorded after reduction of the nitroxide **13** to corresponding ammonium salt **14** with Zn in the presence of trifluoroacetic acid (Scheme 9).

The remaining two products were colorless diamagnetic oils. The ^1^H and ^13^C NMR spectra of both compounds revealed broadening of some signals similarly to those of **5c**. A detailed analysis of the ^1^H-^13^C HMBC, ^1^H-^13^C HSQC, and ^1^H-^1^H COSY spectra of isolated compounds (Figures S48–S53, Appendix A) allowed us to conclude that major and minor products are isomeric octahydrocyclopenta[*c*]azepines **4d** and **5d**. The presence of impurity signals (singlet signal at 1.24 ppm, multiplet signals of five methylene groups in the region of 1.70–2.40 ppm partially overlapped with the signals of the major isomer **5d**, the singlet signal of the methylene group at the heteroatom at 3.78 ppm in ^1^H NMR spectrum and two signals in the low-field region at 130.84 and 138.45 ppm in ^13^C NMR spectrum) of some **5d** samples may indicate the formation of the third isomer **6d**; however, this isomer was not isolated and no other confirmation for this was obtained.

**Scheme 10:**
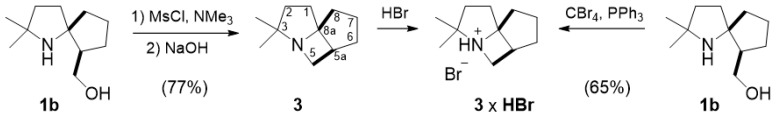
Reaction of **1b** with MsCl/NMe_3_ or PPh_3_-CBr_4_.

Formation of both **4** and **5** presumably occurs via corresponding benzyl- or benzoyloxyammonium salts **12**. However, we did not find references on perhydro-cyclopenta[2,3]azeto[1,2-*a*]pyrrolium salts in literature. To the best of our knowledge, similar ring junction was never observed before, except for *cis*,*cis*,*cis*,*cis*-[5.5.5.4]-1-azafenestrane derivatives [7]. These strained structures may be unstable themselves or in the presence of bases, which are present in the reaction mixtures during mesylation or Appel reaction. So easy cleavage of C-N bond in perhydro-cyclopenta[2,3]azeto[1,2-*a*]pyrrolium salts inspired us to study the behavior of ((5*R*(*S*),6*R*(*S*))-6-(hydroxymethyl)-2,2-dimethyl-1-azaspiro[4.4]nonane **1b** under Appel reaction conditions and mesylation. Both reactions afforded the same amine **3** with the yield of 65–77%. The amine **3** was characterized as a hydrobromide, a colorless crystalline compound (Scheme 10).

**Figure 1:**
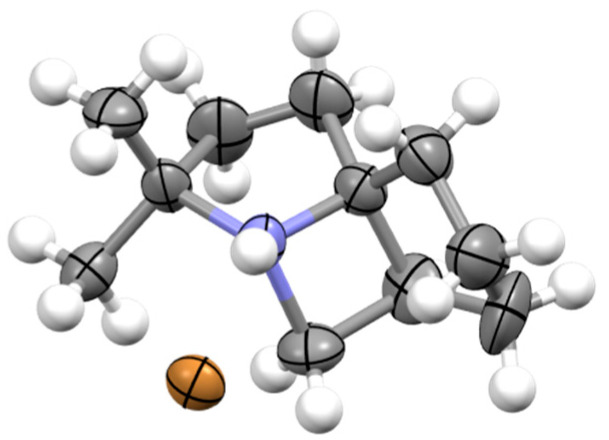
The structure of **3×HBr** according to X-ray analysis.

Analysis of the ^1^H NMR spectrum fine structure of the obtained substance, in addition to signals of pair methyl groups, indicates the presence of two isolated spin systems (Figures S60 and S61, Table S2). To confirm the structure of isolated substance, ^1^H-^1^H COSY, ^1^H-^13^C HMBC, and ^1^H-^13^C HSQC NMR spectra were recorded (Figures S39–S41, Appendix A). In the ^1^H-^13^C HMBC spectrum, the long-range interactions are observed between two geminal hydrogen atoms in the weakest field (i.e., adjacent to heteroatom) and carbons of geminal methyl groups and node carbon atom, confirming C(5)-N bond formation. The single crystal X-ray analysis of the hydrobromide **3×HBr** provided unambiguous confirmation of the structure (Figure 1, see Figure S59).

**Scheme 11:**
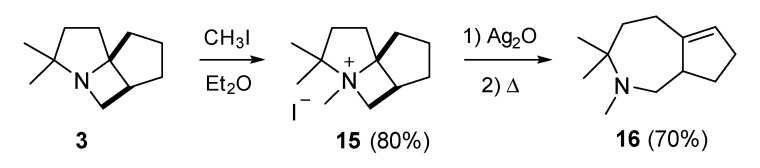
Methylation of **3** with subsequent Hofmann cleavage of quaternary ammonium hydroxide.

The reaction of amine **3** with an excess of methyl iodide gave a crystalline substance, the NMR spectra of which are very similar to the spectra of **3×HBr**, however, in ^1^H NMR, a singlet with the intensity of 3H at 2.94 ppm is observed, and in ^13^C NMR, a signal at 38.72 ppm is observed, which confirms methylation at the nitrogen atom with the formation of **15**, while maintaining the strained tricyclic structure (Scheme 11).

Salt **15** was then treated with wet silver (I) oxide and heating of the resulting quaternary ammonium hydroxide gave a mixture of several compounds. According to GC-MS data, two compounds with M = 179 g/mol (that corresponds to elimination of HI) were observed in the reaction mixture, 83 and 1%, and a product with M = 197 g/mol (14% according to GC) (Figure S58, Table S1). The latter compound may correspond to the amino alcohol, a typical byproduct in Hofmann elimination conditions, a result of the substitution reaction [27]. The preparative yield of the main product was 70%, the minor products were not isolated. Analysis of the ^1^H and ^13^C NMR data allows us to conclude that the isolated product contains a double bond of the >C=CH type; therefore, the assumption of the possible formation of a spirocyclic structure with an exomethylene fragment should be rejected. 2D-NMR spectra (Figures S54–S56, Appendix A) confirmed the octahydrocyclopenta[*c*]azepine structure of compound **16** and showed that the double carbon-carbon bond is located in the 5-membered ring.

## 3. Materials and Methods

### 3.1. General Information

The IR spectra were recorded on a Bruker Vector 22 FT-IR spectrometer (Bruker, Billerica, MA, USA) in KBr pellets (1:150 ratio) or in neat samples (for oily compounds) (see Figures S1–S11). UV spectra were acquired on a HP Agilent 8453 spectrometer (Agilent Technologies, Santa Clara, CA, USA) in ethanol solutions (concentration ~10^−4^ M) (see Figure S12). ^1^H NMR spectra were recorded on a Bruker AV 300 (300.132 MHz), AV 400 (400.134 MHz), AV III 500 (500.030 MHz), AV 600 (600.300 MHz), and DRX 500 (500.130 MHz) spectrometers (Bruker, Billerica, MA, USA). ^13^C NMR spectra were recorded on a Bruker AV 300 (75.467 MHz), AV 400 (100.614 MHz), AV III 500 (125.730 MHz), AV 600 (150. 945 MHz), and DRX 500 (125.758 MHz) spectrometers (see Figures S13–S56). ^15^N NMR spectrum was obtained on a Bruker Avance III 500 FT-spectrometer (Bruker, Billerica, MA, USA) (50.67 MHz) as projection of 2D ^1^H-^15^N-correlation. The ^15^N NMR chemical shifts are referred to external standard of formamide (δ(^15^N) = 112.5 ppm). All the NMR spectra were acquired for 5–10% solutions in CDCl_3_, CD_3_OD, or CDCl_3_-CD_3_OD mixtures at 300 K or in DMSO-*d*_6_ at 333 K using the signal of the solvent as a standard. HRMS analyses were performed with High Resolution Mass Spectrometer DFS (Thermo Electron, Waltham, MA, USA). GC-MS analyses were performed with Chromato-mass spectrometer Agilent 6890 MSD Agilent 5973. HPLC analyses were carried out using an HPLC-UV (Agilent 1100, Agilent Technologies Inc., USA) with an Zorbax C8 column (250 mm × 4.6 mm with 5 µm particle size; Agilent Technologies Inc., USA), and the mobile phase were delivered at a flow rate of 1.0 mL/min. Sample was dissolved in acetonitrile (25 mg/mL). The injection volume was 20 µL, and the column temperature was 35 °C. Mobile phase used was a mixture acetonitrile/water (8:2 *v/v*). 

The structure of compound **3×HBr** was determined by single-crystal X-ray analysis (see SI). The X-ray diffraction experiment was carried out on a Bruker KAPPA APEX II diffractometer (graphite-monochromated Mo Kα radiation). Reflection intensities were corrected for absorption by SADABS program [30]. The structure of salt **3×HBr** was solved by direct methods using the SHELXS-97 program [31] and refined as a 2-component inversion twin by anisotropic (isotropic for all H atoms) full-matrix least-squares method against F2 of all reflections by SHELXL-2014 [32]. The positions of the hydrogen atoms were calculated geometrically and refined in riding model. Crystallographic data for **3×HBr** have been deposited at the Cambridge Crystallographic Data Centre as supplementary publication no. CCDC 1940904. Copy of the data can be obtained, free of charge, on application to CCDC, 12 Union Road, Cambridge CB21EZ, UK; fax: (+44) 122 3336033 or e-mail: deposit@ccdc.cam.ac.uk; internet: www.ccdc.cam.ac.uk (accessed on 13 August 2021). 

The reactions were monitored by thin layer chromatography (TLC) on Merck TLC Silica gel 60 F_254_ plates or Merck TLC Aluminium oxide 60 F_254_, neutral, plates. Kieselgel 60 (Macherey-Nagel GmbH & Co. KG, Düren, Germany) and alumina were utilized as sorbent for the column chromatography.

The EPR spectrum of nitroxide 13 in methanol solution was recorded with an Elexsys E540 X-band spectrometer (Bruker, Billerica, MA, USA) in a 100 µL quartz capillary for 0.1 mM solution, with the following spectrometer settings: field center, 351.600 mT; sweep range, 10 mT; modulation amplitude, 0.15 mT; microwave power, 2 mW; time constant, 10.24 ms; and scan time, 21.39 ms. The EasySpin software (Version 5.2.28, easyspin.org, [33]) was employed for simulation of spectra.

### 3.2. Synthesis

#### 3.2.1. Reaction of 6-(Hydroxymethyl)-2,2-dimethyl-1-azaspiro[4.4]nonan-1-oxyl **1a** with Methanesulfonyl Chloride

Triethylamine (0.174 g, 1.71 mmol) was added to a solution of nitroxide **1a** (0.20 g, 1.01 mmol) in dry CHCl_3_ (5 mL) at 0 °C. Then MsCl (0.139 g, 1.21 mmol) was added dropwise to mixture under cooling on an ice bath. The mixture was stirred at room temperature for 6 h. The reaction was monitored by TLC (SiO_2_, hexane-diethyl ether 1:1 mixture; stained with Dragendorff’s reagent, R_f_(**2**) = 0.45). The reaction mixture was washed with a saturated solution of NaCl (2×10 mL), the organic layer was dried with anhydrous Na_2_SO_4_. After evaporation of the solvent under reduced pressure the crude residue was purified by column chromatography (SiO_2_, hexane-diethyl ether 1:1 mixture as an eluent) to give *3,3-dimethyloctahydrocyclopenta[c]pyrrolo[1,2-b]isoxazole* (**2**): 0.119 g, yield 65%. Physical properties and spectral characteristics coincide to the literature data [4].

#### 3.2.2. Reaction of 6-(Hydroxymethyl)-2,2-dimethyl-1-azaspiro[4.4]nonan-1-oxyl **1a** with PPh_3_-CBr_4_

Carbon tetrabromide (0.84 g, 2.66 mmol) and PPh_3_ (0.70 g, 2.66 mmol) were added to a solution of nitroxide **1a** (0.25 g, 1.26 mmol) in dry CH_2_Cl_2_ (6 mL) and the reaction mixture was stirred at room temperature for 12 h to complete the reaction. The progress of reaction was monitored by TLC (SiO_2_, hexane-diethyl ether 1:1 mixture; stained with Dragendorff’s reagent). After evaporation of the solvent under reduced pressure, the crude residue was purified by column chromatography (SiO_2_, hexane-diethyl ether 1:1 mixture as an eluent) to give **2**, 0.091 g, yield 40%. Physical properties and spectral characteristics coincide to the literature data [4].

#### 3.2.3. Reaction of (2,2-Dimethyl-1-azaspiro[4.4]nonan-6-yl)methanol **1b** with Methanesulfonyl Chloride

Approximately three-fold excess of cold liquid NMe_3_ (1.53 mL, 17.34 mmol), prepared from aqueous solution of NMe_3_ and excess of solid NaOH, was added to the solution of **1b** (1.058 g, 5.78 mmol) in dry CHCl_3_ at 0 °C. Then MsCl (1.06 g, 9.25 mmol) was added dropwise to the mixture under cooling on an ice bath and the reaction mass was stirred at room temperature for 12 h. The progress of the reaction was monitored by TLC (SiO_2_, methanol-ethyl acetate 1:4 mixture as an eluent; stained with iodine vapor, R_f_(**3salt**) = 0.15). The reaction mixture was evaporated under reduced pressure. The residue was dissolved in excess of aqueous solution of NaOH and extracted with diethyl ether (3 × 10 mL). The combined organic layers were dried with anhydrous Na_2_CO_3_. After removal of the solvent under atmosphere pressure, the crude residue was purified by vacuum sublimation (P = 14 mm Hg, T = 110–120 °C) to give **3**.

*(5aS(R),8aR(S))-3,3-Dimethyloctahydrocyclopenta[2,3]azeto[1,2-a]pyrrole* (**3**): 0.73 g, yield 77%. Colorless liquid. IR (neat) ν_max_: 2943, 2860, 2829, 1460, 1444, 1429, 1379, 1363, 1333, 1302, 1290, 1280, 1257, 1246, 1232, 1223, 1207, 1196, 1184, 1147, 1134, 1101, 1084, 1070, 1037, 980, 970, 951, 926, 904, 897, 872, 769, 717, 627 cm^−1^; ^1^H NMR (600 MHz; CDCl_3_, *δ*): 0.91 (s, 3H, CH_3_), 1.08 (s, 3H, CH_3_), 1.37 (ddd, *J*_*d*1_ = 6.5 Hz, *J*_*d*2_ = 12.9 Hz, *J*_*d*3_ = 13.0 Hz, 1H, C(8)H_2_), 1.51 (dddd, *J*_*d*1_ = 7.2 Hz, *J*_*d*2_ = 8.1 Hz, *J*_*d*3_ = 12.5 Hz, *J_d4_* = 12.08 Hz, 1H, C(6)H_2_), 1.59 (dd, *J*_*d*1_ = 6.5 Hz, *J*_*d*2_ = 11.5 Hz, 1H, C(2)H_2_), 1.61 (dd, *J*_*d*1_ = 7.0 Hz, *J*_*d*2_ = 12.8 Hz, 1H, C(6)H_2_), 1.65 (dd, *J*_*d*1_ = 6.4 Hz, *J*_*d*2_ = 13.0 Hz, 1H, C(8)H_2_), 1.74 (dd, *J*_*d*1_ = 7.5 Hz, *J*_*d*2_ = 12.9 Hz, 1H, C(1)H_2_), 1.81 (ddd, *J*_*d*1_ = 6.5 Hz, *J*_*d*2_ = 7.2 Hz, *J*_*d*3_ = 12.5 Hz, 1H, C(7)H_2_), 1.88 (ddd, *J_d_*_1_ = 6.5 Hz, *J*_*d*2_ = 12.7 Hz, *J*_*d*3_ = 12.9 Hz, 1H, C(1)H_2_), 1.96 (ddd, *J_d_*_1_ = 7.5 Hz, *J*_*d*2_ = 11.5 Hz, *J*_*d*3_ = 12.7 Hz, 1H, C(2)H_2_), 2.15 (dddt, *J*_*d*1_ = 6.4 Hz, *J*_*d*2_ = 7.0 Hz, *J*_*d*3_ = 12.7 Hz, *J_t_* = 12.5 Hz, 1H, C(7)H_2_), 2.29 (ddd, *J*_*d*1_ = 4.4 Hz, *J*_*d*2_ = 8.1 Hz, *J*_*d*3_ = 9.3 Hz, 1H, C(5a)H), 2.76 (dd, *J*_*d*1_ = 4.4 Hz, *J*_*d*2_ = 9.8 Hz, 1H, C(5)H_2_), 3.30 (dd, *J*_*d*1_ = 9.3 Hz, *J*_*d*2_ = 9.8 Hz, 1H, C(5)H_2_); ^13^C{^1^H} NMR (150 MHz; CDCl_3_, *δ*): 22.16 (CH_3_), 25.89 C(7), 27.85 (CH_3_), 31.14 C(6), 33.83 C(1), 36.95 C(2), 38.94 C(5a), 39.37 C(8), 48.96 C(5), 61.95 C(3), 82.21 C(8a).

#### 3.2.4. Reaction of (5a*S*,8a*R*)-3,3-Dimethyloctahydrocyclopenta[2,3]azeto[1,2-a]pyrrole **3** with HBr

An aqueous solution of HBr was added dropwise to a solution of amine **3** (0.27 g, 1.64 mmol) in diethyl ether (3 mL) with stirring to pH = 2–3. Then the organic and aqueous phases were separated, the aqueous one was evaporated to dryness under reduced pressure. The solid residue was crystallized from iso-propanol to give **3×HBr**.

*(5aS(R),8aR(S))-3,3-Dimethyloctahydro-1H-cyclopenta[2,3]azeto[1,2-a]pyrrol-4-ium bromide* (**3×HBr**): 0.35 g, yield 87%. Colorless crystals, m.p. 194.5 °C with decomposition (iso-propanol). Elemental analysis: found: C, 53.66; H, 8.11; N, 5.70; Br, 32.49; calcd. for C_11_H_20_BrN: C, 53.67; H, 8.19; N, 5.69; Br, 32.46%; IR (KBr) ν_max_: 3014, 2960, 2928, 2868, 2800, 2754, 2733, 2673, 2950, 2632, 2596, 2571, 2546, 2521, 2496, 2482, 2442, 2415, 2363, 2334, 1470, 1446, 1425, 1406, 1387, 1381, 1350, 1331, 1317, 1304, 1273, 1242, 1213, 1192, 1171, 1146, 1117, 1090, 1065, 1034, 997, 985, 964, 947, 927, 893, 862, 845, 804, 787, 650, 627, 588, 486 cm^−1^; ^1^H NMR (600 MHz; CDCl_3_, *δ*): 1.39 (s, 3H, CH_3_), 1.46 (s, 3H, CH_3_), 1.82 (dddd, *J*_*d*1_ = 6.5 Hz, *J*_*d*2_ = 7.1 Hz, *J*_*d*3_ = 13.0 Hz, *J_d4_* = 13.7 Hz, 1H, C(6)H_2_), 1.89 (dd, *J*_*d*1_ = 6.6 Hz, *J*_*d*2_ = 13.7 Hz, 1H, C(6)H_2_), 1.90 (ddd, *J*_*d*1_ = 6.9 Hz, *J*_*d*2_ = 13.1 Hz, *J*_*d*3_ = 14.8 Hz, 1H, C(8)H_2_), 2.11 (dd, *J*_*d*1_ = 6.4 Hz, *J*_*d*2_ = 13.4 Hz, 1H, C(2)H_2_), 2.13 (ddd, *J*_*d*1_ = 6.5 Hz, *J*_*d*2_ = 6.9 Hz, *J*_*d*3_ = 13.3 Hz, 1H, C(7)H_2_), 2.19 (dd, *J*_*d*1_ = 7.1 Hz, *J*_*d*2_ = 13.7 Hz, 1H, C(1)H_2_), 2.22 (dd, *J*_*d*1_ = 6.1 Hz, *J*_*d*2_ = 14.8 Hz, 1H, C(8)H_2_), 2.30 (ddddd, *J*_*d*1_ = 6.1 Hz, *J*_*d*2_ = 6.5 Hz, *J*_*d*3_ = 13.0 Hz, *J_d4_* = 13.1 Hz, *J_d5_* = 13.3 Hz, 1H, C(7)H_2_), 2.36 (ddd, *J*_*d*1_ = 6.4 Hz, *J*_*d*2_ = 13.3 Hz, *J*_*d*3_ = 13.7 Hz, 1H, C(1)H_2_), 2.53 (ddd, *J*_*d*1_ = 7.1 Hz, *J*_*d*2_ = 13.3 Hz, *J*_*d*3_ = 13.3 Hz, 1H, C(2)H_2_), 2.80 (ddd, *J*_*d*1_ = 5.6 Hz, *J*_*d*2_ = 7.1 Hz, *J*_*d*3_ = 9.4 Hz, 1H, C(5a)H), 3.51 (dd, *J*_*d*1_ = 5.6 Hz, *J*_*d*2_ = 12.3 Hz, 1H, C(5)H_2_), 4.19 (dd, *J*_*d*1_ = 9.4 Hz, *J*_*d*2_ = 12.3 Hz, 1H, C(5)H_2_); ^13^C{^1^H} NMR (150 MHz; CDCl_3_, *δ*): 21.31 (CH_3_), 24.80 (CH_3_), 25.79 C(7), 31.34 C(6), 32.53 C(1), 37.38 C(8), 37.42 C(2), 39.48 C(5a), 49.85 C(5), 67.74 C(3), 91.79 C(8a).

#### 3.2.5. Reaction of ((5*R*(*S*),6*R*(*S*))-2,2-Dimethyl-1-azaspiro[4.4]nonan-6-yl)methanol **1b** with PPh_3_-CBr_4_

Carbon tetrabromide (1.80 g, 5.53 mmol) and PPh_3_ (1.45 g, 5.53 mmol) were added to a solution of **1b** (0.40 g, 2.21 mmol) in dry CH_2_Cl_2_ (8 mL) and the reaction mixture was stirred at room temperature for 12 h to complete the reaction. The progress of reaction was monitored by TLC (SiO_2_, methanol-ethyl acetate 1:6 mixture; stained with iodine vapor, R_f_(**3×HBr**) = 0.2). After evaporation of the solvent under reduced pressure, the crude residue was purified by column chromatography (SiO_2_, methanol-ethyl acetate 1:5 mixture) and recrystallized from *iso*-propanol to give **3×HBr**, 0.35 g, yield 65%.

#### 3.2.6. (1-(Benzyloxy)-2,2-dimethyl-1-azaspiro[4.4]nonan-6-yl)-methanol (**1c**)

An excess of KOH suspension in methanol-*iso*-propanol 1:3 mixture (8 mL) was added to a solution of alkoxyamine **9** [4] (0.342 g, 1.03 mmol) in iso-propanol (25 mL). The reaction mixture was left at room temperature for 12 h to complete the reaction. The progress of reaction was monitored by TLC (SiO_2_, hexane-diethyl ether 1:1 mixture; stained with Dragendorff’s reagent, R_f_(**1c**) = 0.45). Methanol and *iso*-propanol were evaporated under reduced pressure. A saturated solution of NaCl (15 mL) was added to the residue, and the resulting mixture was extracted with CHCl_3_ (3 × 15 mL). The combined organic extracts were dried with anhydrous Na_2_SO_4_. After evaporation of the solvent under reduced pressure, the crude residue was purified by column chromatography (SiO_2_, hexane-diethyl ether 1:1 mixture) to give **1c**.

*(1-(Benzyloxy)-2,2-dimethyl-1-azaspiro[4.4]nonan-6-yl)-methanol* (**1c**): 0.28 g, yield 94%. Colorless oil. Elemental analysis: found: C, 74.85; H, 9.46; N, 4.91; calcd. for C_18_H_27_NO_2_: C, 74.70; H, 9.40; N, 4.73%; IR (neat) ν_max_: 3427, 3089, 3064, 3032, 2958, 2870, 1608, 1497, 1454, 1363, 1317, 1257, 1155, 1082, 1028, 908, 845, 752, 735, 696, 613 cm^−1^; ^1^H NMR (500 MHz; CDCl_3_, *δ*): 1.21 (s, 3H), 1.31 (s, 3H), 1.40–1.57 (m, 3H), 1.60–1.80 (m, 5H), 1.80–1.90 (m, 2H), 2.30–2.38 (m, 1H), 3.78–3.82 (m, 2H), 4.76 (d, *J_d_* = 10 Hz, 1H), 4.94 (d, *J_d_* = 10 Hz, 1H), 5.56 (br.s, 1H), 7.25–7.36 (m, 5H); ^13^C{^1^H} NMR (125 MHz; CDCl_3_, *δ*): 22.55, 22.81, 27.15, 29.96, 34.44, 35.99, 36.09, 50.41, 64.47, 66.57, 76.46, 78.81, 127.83, 128.17, 128.39, 137.12.

#### 3.2.7. Reaction of (1-(Benzyloxy)-2,2-dimethyl-1-azaspiro[4.4]nonan-6-yl)-methanol **1c** with Methanesulfonyl Chloride

Triethylamine (0.62 g, 6.16 mmol) was added to a solution of **1c** (1.018 g, 3.52 mmol) in dry CHCl_3_ (15 mL) at 0 °C. Then MsCl (0.605 g, 5.29 mmol) was added dropwise to the mixture upon stirring and cooling on an ice bath, and the reaction mass was left at room temperature for 72 h. The reaction was monitored by TLC (SiO_2_, hexane-diethyl ether 1:2 mixture, stained with Dragendorff’s reagent; R_f_(**1c**) = 0.55). The mixture was concentrated under reduced pressure, the crude residue was subjected to column chromatography (SiO_2_, hexane-diethyl ether 20:1 mixture, stained with Dragendorff’s reagent; R_f_(**4c**) = 0.4, R_f_(**5c**) = 0.3, and then: hexane-diethyl ether 1:2 mixture, R_f_(**2**) = 0.45) to give **2**, **5c,** and a mixture of **4c** and **6c** 10:1 with preparative yields 13, 12, and 48%, respectively. Pure **4c** was isolated using preparative HPLC (Zorbax C8 (250 mm × 4.6 mm, i.d., 5 µm); mobile phase: acetonitrile/water (8:2 *v/v*).

*2-(Benzyloxy)-3,3-dimethyl-1,2,3,4,5,7,8,8a-octahydrocyclopenta[c]azepine* (**4c**): Colorless oil. Elemental analysis: C, 79.86; H, 9.21; N, 5.17; calcd. for C_18_H_25_NO: C, 79.66; H, 9.28; N, 5.16%; HRMS (EI/DFS) *m*/*z* [M]^+^ calcd. for (C_18_H_25_NO)^+^: 271.1931; found: 271.1932. IR (neat) ν_max_: 3109, 3090, 3066, 3034, 2931, 2850, 1944, 1647, 1606, 1497, 1454, 1433, 1379, 1362, 1307, 1288, 1265, 1242, 1209, 1176, 1155, 1140, 1126, 1084, 1041, 1030, 997, 958, 931, 910, 870, 847, 733, 696, 665, 648, 617, 600 cm^−1^; ^1^H NMR (400 MHz; CDCl_3_, *δ*): 1.07 (s, 3H, CH_3_), 1.25 (s, 3H, CH_3_), 1.32 (ddd, *J*_*d*1_ = 3.1 Hz, *J*_*d*2_ = 7.1 Hz, *J*_*d*3_ = 14.6 Hz, 1H, C(4)H_2_), 1.34 (tdd, *J_t_* = 7.5 Hz, *J*_*d*1_ = 9.0 Hz, *J*_*d*2_ = 12.6 Hz, 1H, C(8)H_2_), 1.85–1.95 (m, 1H, C(4)H_2_), 2.01 (dtd, *J*_*d*1_ = 4.1 Hz, *J_t_* = 8.0 Hz, *J*_*d*2_ = 12.6 Hz, 1H, C(8)H_2_), 2.17–2.26 (m, 2H, C(7)H_2_), 2.26–2.38 (m, 2H, C(5)H_2_), 2.82 (dd, *J*_*d*1_ = 10.7 Hz, *J*_*d*2_ = 13.2 Hz, 1H, C(1)H_2_), 2.90–2.98 (m, 1H, C(8a)H), 3.07 (dd, *J*_*d*1_ = 3.4 Hz, *J*_*d*2_ = 13.2 Hz, 1H, C(1)H_2_), 4.64 (d, *J_d_* = 10.8 Hz, 1H, CH_2_Ph), 4.67 (d, *J_d_* = 10.8 Hz, 1H, CH_2_Ph), 5.29–5.32 (m, 1H, C(6)H), 7.25–7.29 (m, 1H, Ph), 7.31–7.37 (m, 4H, Ph); ^13^C{^1^H} NMR (100 MHz; CDCl_3_, *δ*): 19.81 (CH_3_), 24.27 C(5), 28.87 (CH_3_), 29.53 C(8), 30.87 C(7), 36.75 C(4), 45.41 C(8a), 57.66 C(1), 61.35 C(3), 75.44 (CH_2_Ph), 122.95 C(6), 127.48 (Ph), 128.10 (Ph), 128.22 (Ph), 137.73 (Ph), 147.92 C(5a).

*2-(Benzyloxy)-3,3-dimethyl-1,2,3,4,6,7,8,8a-octahydrocyclopenta[c]azepine* (**5c**): 0.11 g, yield 12%. Colorless oil. HRMS (EI/DFS) *m*/*z* [M]^+^ calcd. for (C_18_H_25_NO)^+^: 271.1931; found: 271.1927. IR (neat) ν_max_: 3088, 3063, 3030, 2947, 2897, 2864, 1606, 1587, 1497, 1452, 1433, 1377, 1360, 1331, 1306, 1275, 1221, 1194, 1176, 1155, 1132, 1105, 1082, 1040, 1005, 968, 935, 912, 876, 852, 825, 798, 748, 733, 696, 677, 654, 634, 609, 600, 565, 542, 463; ^1^H NMR (500 MHz; DMSO-*d*_6_, 333 K, *δ*): 0.98 (s, 3H, CH_3_), 1.20 (s, 3H, CH_3_), 1.20–1.28 (m, 1H, C(8)H_2_), 1.36–1.46 (m, 1H, C(7)H_2_), 1.60–1.68 (m, 1H, C(7)H_2_), 1.72–1.82 (m, 1H, C(4)H_2_), 1.83–1.93 (m, 1H, C(8)H_2_), 2.23 (br.s, 2H, C(6)H_2_), 2.44 (br.s, 1H, C(4)H_2_), 2.68 (m, 1H, C(1)H_2_), 2.78 (br.s, 1H, C(8a)H), 3.04 (dd, *J*_*d*1_ = 12.4 Hz, *J*_*d*2_ = 2.4 Hz, 1H, C(1)H_2_), 4.58–4.66 (m, 2H, CH_2_Ph), 5.36–5.42 (m, 1H, C(5)H), 7.24–7.36 (m, 5H, Ph); ^13^C{^1^H} NMR (125 MHz; DMSO-*d*_6_, 333 K, *δ*): 18.75 (CH_3_), 25.12 C(7), 29.57 (CH_3_), 32.04 C(8), 33.31 C(6), 36.91 (C4), 39.00 C(8a), 53.87 C(1), 59.20 C(3), 74.40 (CH_2_Ph), 116.72 C(5), 127.34 (Ph), 127.96 (Ph), 128.08 (Ph), 137.57 (Ph), 149.34 C(5a); ^15^N NMR (51 MHz, HCONH_2_, DMSO-*d*_6_, *δ*): 185.7.

*2-(Benzyloxy)-3,3-dimethyl-1,2,3,4,5,6,7,8-octahydrocyclopenta[c]azepine* (**6c**): (the data from the NMR spectrum of the mixture): ^1^H NMR (500 MHz; DMSO-*d*_6_, 333 K, *δ*): 1.18 (s, 6H), 1.65–1.69 (m, 2H), 1.75–1.83 (m, 2H), 2.05–2.10 (m, 2H), 2.15–2.40 (m, 4H), 3.50 (s, 2H), 4.63 (br.s., 2H), 7.30–7.42 (m. 5H); ^13^C{^1^H} NMR (125 MHz; DMSO-*d*_6_, 333 K, *δ*): 22.45, 24.33, 24.90, 37.66, 37.80, 38.89, 51.87, 61.38, 75.71, 128.09, 128.65, 129.04, 133.04, 138.17, 138.27.

#### 3.2.8. Reaction of (1-(Benzyloxy)-2,2-dimethyl-1-azaspiro[4.4]nonan-6-yl)-methanol **1c** with PPh_3_-CBr_4_

Carbon tetrabromide (1.21 g, 3.63 mmol) and PPh_3_ (0.98 g, 3.75 mmol) were added to a solution of **1c** (0.35 g, 1.21 mmol) in dry CHCl_3_ (7 mL) and the reaction mixture was stirred at room temperature for 48 h. The reaction was monitored by TLC (SiO_2_, hexane-diethyl ether 1:2 mixture). After evaporation of the solvent under reduced pressure, the crude residue was subjected to column chromatography (SiO_2_, hexane-diethyl ether 20:1 mixture as an eluent) to give a mixture of **4c**, **5c,** and **6c** (0.111 g, yield 34%).

#### 3.2.9. 6-(Hydroxymethyl)-2,2-dimethyl-1-azaspiro[4.4]nonan-1-yl benzoate (**1d**)

Manganese dioxide (7.47 g, 85 mmol) was added to a solution of nitroxide **1а** (0.85 g, 4.29 mmol) in diethyl ether (20 mL). A solution of benzhydrazide (1.16 g, 8.58 mmol) in methanol was added dropwise to the resulting mixture, and the reaction mass was stirred for 30 min. Then manganese dioxide was filtered off through celite, the filtrate was evaporated, and the residue was purified by column chromatography (SiO_2_, hexane-diethyl ether 1:1 mixture as an eluent, detected under UV lamp and stained with Dragendorff’s reagent, R_f_(**1d**) = 0.45).

*6-(Hydroxymethyl)-2,2-dimethyl-1-azaspiro[4.4]nonan-1-yl benzoate* (**1d**): 1.06 g, yield 81%. Yellowish oil. Elemental analysis: C, 70.98; H, 8.41; N, 4.86; calcd. for C_18_H_25_NO_3_: C, 71.26; H, 8.31; N, 4.62%; HRMS (EI/DFS) *m*/*z* [M]^+^ calcd. for (C_18_H_25_NO_3_)^+^: 303.1829; found: 303.1826. IR (neat) ν_max_: 3452, 3088, 3064, 3032, 2958, 2875, 1740, 1601, 1583, 1491, 1452, 1410, 1385, 1367, 1315, 1261, 1244, 1176, 1113, 1082, 1063, 1026, 1001, 976, 947, 924, 897, 885, 854, 802, 710, 687, 667, 646, 617 cm^−1^; NMR (400 MHz; CDCl_3_, *δ*): 1.19 (s, 3H), 1.25 (s, 3H), 1.38–1.56 (m, 2H), 1.56–1.69 (m, 3H), 1.70–1.92 (m, 4H), 2.04 (dt, *J_d_* = 12.4 Hz, *J_t_* = 8.5 Hz, 1H), 2.32–2.41 (m, 1H), 3.61 (dd, *J*_*d*1_ = 6.8 Hz, *J*_*d*2_ = 12.1 Hz, 1H), 3.80 (dd, *J*_*d*1_ = 2.5 Hz, *J*_*d*2_ = 12.1 Hz, 1H), 5.29 (br.s, 1H), 7.39–7.46 (m, 2H), 7.52–7.59 (m, 1H), 7.97–8.03 (m, 2H); ^13^C{^1^H} NMR (100 MHz; CDCl_3_, *δ*): 21.88, 22.35, 26.05, 27.93, 35.08, 35.41, 35.46, 51.50, 62.32, 67.59, 77.79, 128.51, 128.40, 129.31, 133.17, 166.01.

#### 3.2.10. Reaction of (1-(Benzoyloxy)-2,2-dimethyl-1-azaspiro[4.4]nonan-6-yl)-methanol **1d** with PPh_3_-CBr_4_

Carbon tetrabromide (0.638 g, 1.92 mmol) and PPh_3_ (0.50 g, 1.92 mmol) were added to a solution of **1d** (0.194 g, 0.64 mmol) in dry CHCl_3_ (6 mL) and the reaction mixture was stirred at room temperature for 12 h. The reaction was monitored by TLC (SiO_2_, hexane-diethyl ether 2:1 mixture; detected under UV lamp and stained with Dragendorff’s reagent). After evaporation of the solvent under reduced pressure, the crude residue was subjected to column chromatography (SiO_2_, from hexane-ethyl acetate 50:1 to hexane-ethyl acetate 25:1 mixture as an eluent) to give **4d**, **5d,** and **13**.

*3,3-Dimethyl-1,4,5,7,8,8a-hexahydrocyclopenta[c]azepin-2(3H)-yl benzoate* (**4d**): 0.055 g, yield 30%. Colorless oil. Elemental analysis: C, 75.88; H, 8.25; N, 4.80; calcd. for C_18_H_23_NO_2_: C, 75.76; H, 8.12; N, 4.62%; HRMS (EI/DFS) *m*/*z* [M]^+^ calcd. for (C_18_H_23_NO_2_)^+^: 285.1723; found: 285.1720. IR (neat) ν_max_: 3088, 3061, 3037, 2976, 2931, 2850, 1741, 1645, 1601, 1583, 1491, 1450, 1383, 1363, 1313, 1259, 1238, 1176, 1157, 1126, 1084, 1063, 1024, 999, 960, 935, 906, 889, 874, 862, 802, 708, 687, 671, 646 cm^−1^; ^1^H NMR (600 MHz; CDCl_3_, *δ*): 1.19 (s, 3H, CH_3_), 1.22 (s, 3H, CH_3_), 1.34 (dddd, *J*_*d*1_ = 6.6 Hz, *J*_*d*2_ = 6.9 Hz, *J*_*d*3_ = 8.7 Hz, *J_d4_* = 13.0 Hz, 1H, C(8)H_2_), 1.46 (ddd, *J*_*d*1_ = 4.1 Hz, *J*_*d*2_ = 5.2 Hz, *J*_*d*3_ = 15.0 Hz, 1H, C(4)H_2_), 2.04 (dddd, *J*_*d*1_ = 4.7 Hz, *J*_*d*2_ = 8.4 Hz, *J*_*d*3_ = 8.7 Hz, *J_d4_* = 12.7 Hz, 1H, C(8)H_2_), 2.07–2.18 (m, 1H, C(4)H_2_), 2.17–2.29 (m, 2H, C(7)H_2_), 2.37–2.48 (m, 2H, C(5)H_2_), 3.06 (dd, *J*_*d*1_ = 11.0 Hz, *J*_*d*2_ = 13.0 Hz, 1H, C(1)H_2_), 3.15–3.29 (m, 2H, C(8a)H + C(1)H_2_), 5.30–5.32 (m, 1H, C(6)H), 7.39–7.43 (m, 2H, Ph), 7.51–7.55 (m, 1H, Ph), 7.98–8.02 (m, 2H, Ph); ^13^C{^1^H} NMR (150 MHz; CDCl_3_, *δ*): 20.30 (CH_3_), 24.23 C(5), 29.29 (CH_3_), 29.41 C(8), 30.77 C(7), 35.55 C(4), 44.02 C(8a), 59.15 C(1), 62.27 C(3), 123.80 C(6), 128.27 (Ph), 129.23 (Ph), 129.58 (Ph), 132.74 (Ph), 146.74 C(5a), 164.97 (C = O).

*3,3-Dimethyl-3,4,6,7,8,8a-hexahydrocyclopenta[c]azepin-2(1H)-yl benzoate* (**5d**): 0.027 g, yield 15%. Colorless oil. HRMS (EI/DFS) *m*/*z* [M]^+^ calcd. for (C_18_H_23_NO_2_)^+^: 285.1723; found: 285.1725. IR (neat) ν_max_: 3088, 3061, 3032, 2949, 2931, 2899, 2866, 2854, 1743, 1601, 1583, 1491, 1450, 1435, 1381, 1362, 1331, 1313, 1257, 1248, 1192, 1176, 1130, 1084, 1063, 1024, 1007, 993, 970, 939, 893, 870, 851, 831, 802, 754, 710, 688, 671 cm^−1^; ^1^H NMR (500 MHz; DMSO-*d*_6_, *δ*): 1.10 (s, 3H, CH_3_), 1.16 (s, 3H, CH_3_), 1.21–1.28 (m, 1H, C(8)H_2_), 1.38–1.49 (m, 1H, C(7)H_2_), 1.61–1.71 (m, 1H, C(7)H_2_), 1.84–1.96 (m, 2H, C(4)H, C(8)H), 2.28 (br.s, 2H, C(6)H_2_), 2.62 (br.s, 1H, C(4)H), 2.91 (br.s, 1H, C(8a)H), 2.99 (t, *J_t_* = 12.4 Hz, 1H, C(1)H_2_), 3.12 (dd, *J*_*d*1_ = 12.4 Hz, *J*_*d*2_ = 2.0 Hz, 1H, C(1)H_2_), 5.44–5.51 (m, 1H, C(5)H), 7.52 (t, *J_t_* = 7.7 Hz, 2H, *m*-Ph), 7.64 (tt, *J*_*t*1_ = 7.5 Hz, *J*_*t*2_ = 1.1 Hz, 1H, *p*-Ph), 7.94 (dd, *J*_*d*1_ = 8.2 Hz, *J*_*d*2_ = 1.1 Hz, 2H, *o*-Ph); ^13^C{^1^H} NMR (125 MHz; DMSO-*d*_6_, *δ*): 19.25 (CH_3_), 25.05 C(7), 29.34 (CH_3_), 31.83 (C8), 33.36 C(6), 36.62 C(4), 38.61 C(8a), 55.36 C(1), 59.89 C(3), 116.70 C(5), 128.69 (*m*-Ph), 128.73 (*o*-Ph), 129.22 (*i*-Ph), 133.03 (*p*-Ph), 149.51(C(5a), 163.84 (C=O); ^15^N NMR (51 MHz, HCONH_2_, DMSO-*d*_6_, *δ*): 194.4.

*6-((Benzoyloxy)methyl)-2,2-dimethyl-1-oxo-1-azaspiro[4.4]nonan-1-oxyl* (**13**): 0.027 g, yield 14%. Yellow crystalline solid, m.p. 68.2 °C with decomposition (hexane). Elemental analysis: C, 71.77; H, 7.97; N, 4.68; calcd. for C_18_H_24_NO_3_: C, 71.50; H, 8.00; N, 4.63%; HRMS (EI/DFS) *m*/*z* [M]^+^ calcd. for (C_18_H_24_NO_3_)^+^: 302.1751; found: 302.1752. IR (KBr) ν_max_: 3070, 3063, 2968, 2928, 2873, 2854, 1716, 1601, 1583, 1491, 1454, 1406, 1371, 1360, 1350, 1313, 1282, 1273, 1250, 1203, 1180, 1163, 1130, 1115, 1084, 1072, 1022, 991, 968, 951, 928, 893, 849, 717, 688, 665, 590, 569, 447 cm^−1^; UV (EtOH) λmax (log ε): 229 (4.06).

#### 3.2.11. Reduction of Nitroxide **13** with Zn in CF_3_COOH for NMR

A suspension of zinc dust (100 mg) in a solution of nitroxide **13** (0.015 g, 0.050 mmol) in CD_3_OD (0.4 mL) in a small glass vial was heated to reflux upon vigorous stirring and trifluoroacetic acid (0.1 mL) was added dropwise. The mixture was stirred for 10–15 min, and the solution was transferred into an NMR tube through a pipette tip with a tightly inserted paper filter. The vial was rinsed with a small portion of CDCl_3_–CD_3_OD mixture and this solution was filtered into the same NMR tube until the normal NMR sample volume was reached.

*(5R(S),6R(S))-6-((Benzoyloxy)methyl)-2,2-dimethyl-1-azaspiro[4.4]nonan-1-ium 2,2,2-trifluoroacetate* (**14**): ^1^H NMR (400 MHz; CDCl_3_–CD_3_OD mixture, *δ*): 1.47 (s, 3H), 1.50 (s, 3H), 1.71–1.87 (m, 2H), 1.88–2.07 (m, 3H), 2.08–2.24 (m, 2H), 2.44–2.56 (m, 1H), 4.36 (dd, *J*_*d*1_ = 7.1 Hz, *J*_*d*2_ = 11.8 Hz, 1H), 4.48 (dd, *J*_*d*1_ = 6.3 Hz, *J*_*d*2_ = 11.8 Hz, 1H), 7.40–7.50 (m, 2H), 7.55–7.63 (m, 1H), 7.93–8.03 (m, 2H).

#### 3.2.12. 3,3,4-Trimethyloctahydro-1*H*-cyclopenta[2,3]azeto[1,2-a]pyrrol-4-ium iodide (**15**)

Iodomethane (0.23g, 1.64 mmol) was added to a solution of **3** (0.09 g, 0.545 mmol) in dry diethyl ether (2 mL) and the reaction mass was left over 12 h. Then the precipitate formed was filtered off and washed with diethyl ether.

*3,3,4-Trimethyloctahydro-1H-cyclopenta[2,3]azeto[1,2-a]pyrrol-4-ium iodide* (**15**): 0.133 g, yield 80%. White crystalline solid, m.p. 187.2–187.4 °C. Elemental analysis: C, 47.19; H, 7.23; N, 4.56; calcd. for C_12_H_22_IN: C, 46.91; H, 7.22; N, 4.59%. IR (KBr) ν_max_: 3007, 2956, 2874, 2833, 2816, 2756, 2600, 2580, 1470, 1443, 1433, 1396, 1383, 1358, 1335, 1315, 1304, 1294, 1275, 1240, 1217, 1196, 1178, 1149, 1138, 1099, 1086, 1068, 1034, 986, 953, 931, 916, 895, 881, 852, 839, 796, 779, 735, 642, 606, 571, 499, 411; ^1^H NMR (300 MHz; CDCl_3_, *δ*): 1.34 (s, 3H), 1.53 (s, 3H), 1.80–2.39 (m, 8H), 2.50 (dd, *J*_*d*1_ = 5.9 Hz, *J*_*d*2_ = 16.0 Hz, 1H), 2.60 (ddd, *J*_*d*1_ = 6.2 Hz, *J*_*d*2_ = 13.0 Hz, *J*_*d*3_ = 13.1 Hz, 1H), 2.77–2.89 (m, 1H), 2.94 (c, 3H), 3.52 (dd, *J*_*d*1_ = 6.2 Hz, *J*_*d*2_ = 12.6 Hz, 1H), 4.65 (dd, *J*_*d*1_ = 9.9 Hz, *J*_*d*2_ = 12.6 Hz, 1H); ^13^C{^1^H} NMR (75 MHz; CDCl_3_, *δ*): 20.72, 24.74, 26.90, 30.09, 33.16, 33.44, 37.80, 38.72, 40.20, 62.65, 74.37, 99.18.

#### 3.2.13. 2,3,3-Trimethyl-1,2,3,4,5,7,8,8a-octahydrocyclopenta[c]azepine (**16**)

Wet silver (I) oxide (5.94 mmol) and water (12 mL) were added to salt **15** (0.729 g, 2.37 mmol) and stirred for 12 h. Then the solid residue was filtered off, the filtrate was concentrated in vacuum. The residue was heated on a water bath under reflux until the disappearance of a solid insoluble in diethyl ether, and then was extracted with diethyl ether. After evaporation of the solvent under reduced pressure, the crude residue was purified by column chromatography (alumina, pentane-diethyl ether 6:1 as an eluent) to give **16**.

*2,3,3-Trimethyl-1,2,3,4,5,7,8,8a-octahydrocyclopenta[c]azepine* (**16**): 0.296 g, yield 70%. Colorless oil. HRMS (EI/DFS) *m*/*z* [M]^+^ calcd. for (C_12_H_21_N)^+^: 179.1669; found: 179.1668. Elemental analysis: C, 80.36; H, 11.82; N, 7.60; calcd. for C_12_H_21_N: C, 80.38; H, 11.81; N, 7.81%. IR (neat) ν_max_: 3466, 3039, 2964, 2945, 2926, 2847, 2798, 2789, 2777, 1649, 1618, 1464, 1454, 1429, 1377, 1362, 1344, 1315, 1288, 1267, 1236, 1205, 1174, 1128, 1109, 1088, 1049, 1024, 1005, 974, 952.7 941, 918, 908, 852, 800, 694, 638, 577, 530, 488. ^1^H NMR (600 MHz; CDCl_3_, *δ*): 0.94 (s, 3H, CH_3_), 1.09 (dd, *J*_*d*1_ = 0.4 Hz, *J*_*d*2_ = 0.7 Hz, 3H, CH_3_), 1.22 (dddd, *J*_*d*1_ = 8.8 Hz, *J*_*d*2_ = 8.9 Hz, *J*_*d*3_ = 9.2 Hz, *J_d4_* = 12.5 H, 1H, C(8)H_2_), 1.32–1.39 (m, 1H, C(4)H_2_), 1.65–1.71 (m, 1H, C(4)H_2_), 1.94 (dddd, *J*_*d*1_ = 5.1 Hz, *J*_*d*2_ = 5.5 Hz, *J*_*d*3_ = 7.8 Hz, *J_d4_* = 12.6 Hz, 1H, C(8)H_2_), 2.15–2.21 (m, 2H, C(7)H_2_), 2.23–2.29 (m, 2H, C(5)H_2_), 2.32 (s, 3H, CH_3_), 2.35 (dd-quartet, *J*_*d*1_ = 4.2 Hz, *J*_*d*2_ = 13.9 Hz, *J_quartet_* = 0.4 Hz, 1H, C(1)H_2_), 2.55 (dd, *J*_*d*1_ = 10.7 Hz, *J*_*d*2_ = 13.9 Hz, 1H, C(1)H_2_), 2.72–2.79 (m, 1H, C(8a)H), 5.20–5.22 (m, 1H, C(6)H); ^13^C{^1^H} NMR (150 MHz; CDCl_3_, *δ*): 20.27 (CH_3_), 24.70 C(5), 28.19 (CH_3_), 29.35 C(8), 30.83 C(7), 38.72 (NCH_3_), 39.95 C(4), 48.35 C(8a), 55.96 C(3), 57.34 C(1), 122.48 C(6), 148.90 C(5a).

## 4. Conclusions

Thus, conversion of hydroxyl group in (5*R*(*S*),6*R*(*S*))-6-(hydroxymethyl)- 2,2-dimethyl-1-X-1-azaspiro[4.4]nonanes (X = H, O·, OBn, OBz) into good leaving group always leads to intramolecular alkylation. The structure of the cyclic products depends on the substituent X at N-1 atom. If X = OBn or OBz, the initially formed 1-OBn- or 1-OBz-1-azoniabicyclo[3.2.0]heptanes undergo spontaneous azetidine ring opening with formation of mixture of isomeric octahydrocyclopenta[*c*]azepine derivatives. Alternatively, thermal degradation of 3,3,4-trimethyloctahydro-1*H*-cyclopen-ta[2,3]azeto[1,2-*a*]pyrrol-4-ium hydroxide gives 2,3,3-trimethyl-1,2,3,4,5,7,8,8a-octa-hydrocyclopenta[*c*]azepine with high selectivity. 

The heterocycles with spiro-(2-hydroxymethyl)cyclopentane moieties can be easily prepared from cyclic nitrones via addition of pent-4-enylmagnesium bromide-oxidation-intramolecular cycloaddition-isoxazolidine ring opening sequence [4,34,35]. Here, we demonstrated an example of conversion of these spirocyclic structures into azepane derivatives. Similar transformations may provide a promising pathway to valuable scaffolds for the synthesis of biologically active compounds.

## Data Availability

Data is contained within the article and supplementary material.

## Supplementary Materials

Figures S1–S11: IR spectra of **1c**–**d**, **3**, **3×HBr**, **4c**–**d**, **5c**–**d**, **13**, **15**, **16**; Figure S12: UV spectrum of **13**; Figures S13–S56: NMR spectra of **1c**–**d**, **3**, **3×HBr**, **4c**–**d**, **5c**–**d**, **13**, **14, 15**, **16**; Figure S57: EPR spectrum of **13**; Figure S58 and Table S1: gas chromatography data of Hofmann elimination of **15**; Figure S59: X-ray analysis data of **3×HBr**; Figures S60, S61 and Table S2: NMR spectrum fine structure analysis of **3×HBr**; Figure S62: HPLC analysis data of **4c** and **6c** mixture and **4c** are available online

## Sample Availability

Samples of the compounds **3**, **15**, **16** are available from the authors.

## Appendix A

### Appendix A.1. 2-(Benzyloxy)-3,3-dimethyl-1,2,3,4,5,7,8,8a-octahydrocyclopenta[c]azepine *(**4c**)*

Assignment of ^1^H and ^13^C signals was carried out using the ^1^H-^13^C HSQC technique (Figure S44). Based on the spectrum, the hydrogen atoms of the methyl, methylene, and methine groups were determined. In the ^1^H-^13^C HMBC spectrum (Figure S43) of **4c**, the long-range interactions were observed between the C(3) atom with chemical shift 61.35 ppm and the hydrogen atoms of the low-field methylene group, apparently adjacent to the heteroatom (C(1)H_2_-group, (dd at 2.82 and dd at 3.07 ppm). Such an interaction could not be observed in the spectrum of **1c,** clearly indicating C(1)H_2_-N bond formation in **4c**. Since the alkoxyammonium structure was discarded, one of the C-N bonds must be broken. The node carbon atom at geminal methyl groups –C(3)– has interactions with protons of two methylene groups С(4)H_2_- (ddd at 1.32 and multiplet at 1.85–1.95 ppm) and C(5)H_2_- (multiplet at 2.26–2.38 ppm), while interaction in ^1^H-^1^H COSY spectrum (Figure S42) shows that the protons of C(5)H_2_- and C(4)H_2_- groups are neighboring. According to the ^1^H-^13^C HMBC spectrum, interactions between the hydrogen atoms of the C(1)H_2_-group and the carbon atoms of the C(8a)H- (45.41 ppm) and C(8)H_2_- (29.53 ppm) groups and the fully substituted carbon atom of the ethylene moiety C(5a) with chemical shift at 147.93 ppm are observed indicating the presence spin system C(1)H_2_-C(8a)H-C(8)H_2_ in **4c**. The long-range interactions are observed between fully substituted carbon atom of ethylene moiety C(5a) and the protons at C(4)H_2_-, C(5)H_2_-, C(7)H_2_- (multiplet at 2.17–2.26 ppm), and C(8)H_2_- (tdd at 1.34 and ddd at 2.02 ppm) groups. The described above interactions correspond to annulated 5- and 7-membered rings system. Since there is no interaction of carbon C(3) with the proton of С(6)H= fragment, the double bond >C=CH is located in 5-membered cyclopentene ring.

Assignments for *3,3-Dimethyl-1,4,5,7,8,8a-hexahydrocyclopenta[c]azepin-2(3H)-yl benzoate* (**4d**) were performed in similar manner.

### Appendix A.2. 2-(Benzyloxy)-3,3-dimethyl-1,2,3,4,6,7,8,8a-octahydrocyclopenta[c]azepine *(**5c**)*

Assignment of ^1^H and ^13^C signals was carried out using the ^1^H-^13^C HSQC technique (Figure S47). Based on the obtained spectrum, the hydrogen atoms of the methyl, methylene, and methine groups were determined. In the ^1^H-^13^C HMBC spectrum of **5c** (Figure S46), the long-range interactions were observed between the C(3) atom with chemical shift 59.20 ppm and the hydrogen atoms of the low-field methylene group, apparently adjacent to the heteroatom (C(1)H_2_-group, br.s. at 2.78 and dd at 3.04 ppm). Such an interaction could not be observed in the spectrum of **1c,** clearly indicating C(1)H_2_-N bond formation in **5c**. Since the alkoxyammonium structure was discarded, one of the C-N bonds must be broken. According to the ^1^H-^13^C HMBC spectrum, the node carbon atom at geminal methyl groups –C(3)– has also interactions with protons of methylene group С(4)H_2_- (multiplet at 1.72–1.82 and br.s. at 2.44 ppm) and C(5)H= (multiplet at 5.36–5.42 ppm), while interaction in ^1^H-^1^H COSY spectrum (Figure S45) shows that the protons of C(4)H_2_- and C(5)H= groups are neighboring. In turn, the hydrogen atom of the C(5)H= group interacts with the protons of the C(6)H_2_- (br.s. at 2.23 ppm) group, which is clear from the ^1^H-^1^H COSY spectrum. Interactions between the hydrogen atoms of the C(7)H_2_-group (multiplet at 1.36–1.46 and multiplet at 1.60–1.68 ppm) with the protons of C(6)H_2_- and C(8)H_2_- (multiplet at 1.20–1.28 and multiplet at 1.83–1.93 ppm) groups in the ^1^H-^1^H COSY spectrum indicate the presence of spin system C(6)H_2_-C(7)H_2_-C(8)H_2_ in the product **5c**. According to the ^1^H-^13^C HMBC spectrum, interactions between the hydrogen atoms of the C(1)H_2_-group and the carbon atoms of the C(8a)H- (39.00 ppm) and C(8)H_2_- (32.04 ppm) groups are observed indicating the presence spin system C(1)H_2_-C(8a)H-C(8)H_2_. Finally, the long-range interactions in the ^1^H-^13^C HMBC spectrum between fully substituted carbon atom of the ethylene moiety C(5a) with chemical shift at 149.34 ppm with hydrogen atoms of C(1)H_2_-, C(6)H_2_-, C(7)H_2_-, C(8)H_2_-, and C(4)H_2_- groups allow us to conclude that the structure of **5c** is annulated 5- and 7-membered rings system with the double bond >C=CH located in 7-membered tetrahydroazepine ring.

### Appendix A.3. 3-Dimethyl-3,4,6,7,8,8a-hexahydrocyclopenta[c]azepin-2(1H)-yl benzoate *(**5d**)*

Assignment of ^1^H and ^13^C signals was carried out using the ^1^H-^13^C HSQC technique (Figure S53). Based on the spectrum, the hydrogen atoms of the methyl, methylene, and methine groups were determined. Analysis of the ^1^H-^1^H COSY spectrum (Figure S51) showed that the hydrogen atom of the =CH fragment interacts with two methylene fragments: C(6)H_2_- and C(4)H_2_-. On the other hand, the COSY spectrum clearly shows the bound fragment C(6)H_2_-C(7)H_2_-C(8)H_2_-C(8a)H, which indicates the presence of the cyclopentane fragment. The result of experiment ^1^H-^13^C HMBC (Figure S52) can also serve as a confirmation of the location of the double bond. The spectrum clearly shows the interaction of one of the methyl groups with the carbon atom of the =CH-fragment, which confirms the orientation of the double bond into the 7-membered ring. The hydrogen atom of this fragment interacts with carbon atoms at 33.36 and 36.62 ppm. The fully-substituted carbon atom of the double bond C(5a) has multiple interactions with the hydrogen atoms of the cyclopentane fragment, while for C(5) this interaction is observed only with the methylene group C(6)H_2_. Cross-peaks from signals at 1.91 ppm in ^1^H-NMR spectrum were not taken into account in the analysis, because this multiplet contains signals from hydrogen atoms of both cycles.

### Appendix A.4. (5aS(R),8aR(S))-3,3-Dimethyloctahydro-1H-cyclopenta[2,3]azeto[1,2-a]pyrrol-4-ium bromide (**3×HBr**) and 2,3,3-Trimethyl-1,2,3,4,5,7,8,8a-octahydrocyclopenta[c]azepine *(**16**)*

Taking the signal C(5a) atom in ^13^C NMR spectra at 39.48 ppm, ^1^H-^13^C HSQC spectra (Figure S41) analysis allowed to assign the signal of a hydrogen atom at C(5a)H at 2.80 ppm. In ^1^H-^1^H COSY (Figure S39) spectrum, this signal has cross-peaks with multiplets at 1.82, 3.51, and 4.19 ppm. On the other hand, signals at 3.51 and 4.19 ppm do not have other cross-peaks. This can only occur if signals at 3.51 and 4.19 ppm belong to C(5) hydrogens. Further analysis of 2D NMR spectra allowed us to assign all signals of carbon and hydrogen atoms. Moreover, the suggested structure is in a good agreement with the simulated spin system (see Figure S60 and S61, Table S2). Finally, the structure of **3×HBr** was confirmed by X-ray data.

Analysis of NMR spectra of **16** was performed in a similar manner and in comparison with spectral data of products **4**–**6**. The signals at 148.9 and 122.5 ppm in ^13^C NMR spectrum were attributed to carbon atoms C(5a) and C(6). In ^1^H NMR spectrum, the signal of C(6)H hydrogen is observed at 5.21 ppm. Analysis of the ^1^H-^1^H COSY spectrum (Figures S54) showed that the hydrogen atom of the =CH fragment interacts with two methylene fragments: C(5)H_2_- and C(7)H_2_- and methine hydrogen at C(8a)H. Further analysis of 2D NMR spectra (Figures S54–S56) demonstrated good agreement with the proposed structure of **16**.
