# Supplementary material for: The Reactions of 6-(Hydroxymethyl)-2,2-dimethyl-1-azaspiro[4.4]nonanes with Methanesulfonyl Chloride or PPh3-CBr4
Source: Molecules. 2021 Oct 2;26(19):6000. doi: 10.3390/molecules26196000 (PMC8512565; doi:10.3390/molecules26196000)
Supplement: Supplementary file 1 [file molecules-26-06000-s001.zip › molecules-1377463-supplementary.pdf]

The reactions of 6-(hydroxymethyl)-2,2-dimethyl-1-azaspiro[4.4]nonanes with methanesulfonyl chloride and PPh_3-CBr_4

Yulia V. Khoroshunova*[1,2], Denis A. Morozov[1], Andrey I. Taratayko[1,2], Sergey A. Dobrynin[1], Ilya V. Yeltsov[2], Tatyana V. Rybalova[1], Yulia S. Sotnikova[1], Dmitriy N. Polovyanenko[1], Nargiz B. Asanbaeva[1,2] and Igor A. Kirilyuk[1]

[1] N.N. Vorozhtsov Institute of Organic Chemistry SB RAS, Academician Lavrentiev Ave. 9, Novosibirsk 630090, Russian Federation; horoshunova@nioch.nsc.ru

[2] Novosibirsk State University, Pirogova Str. 1, Novosibirsk 630090, Russian Federation

* Correspondence: horoshunova@nioch.nsc.ru

Supporting Information

Table of Contents

1. IR spectral data S4

1.1 (5a*S*(*R*),8a*R*(*S*))-3,3-Dimethyloctahydrocyclopenta[2,3]azeto[1,2-*a*]pyrrole (**3**) (**Figure S1**) . S4

1.2 (5a*S*(*R*),8a*R*(*S*))-3,3-Dimethyloctahydro-1*H*-cyclopenta[2,3]azeto[1,2-*a*]pyrrol-4-ium bromide (**3×HBr**) (**Figure S2**) S4

1.3 ((5*R*(*S*),6*R*(*S*)-1-(Benzyloxy)-2,2-dimethyl-1-azaspiro[4.4]nonan-6-yl)-methanol (**1c**) (Figure S3) S5

1.4 2-(Benzyloxy)-3,3-dimethyl-1,2,3,4,5,7,8,8a-octahydrocyclopenta[*c*]azepine (**4c**) (**Figure S4**) S5

1.5 2-(Benzyloxy)-3,3-dimethyl-1,2,3,4,6,7,8,8a-octahydrocyclopenta[*c*]azepine (**5c**) (**Figure S5**) S6

1.6 (5*R*(*S*),6*R*(*S*))-6-(Hydroxymethyl)-2,2-dimethyl-1-azaspiro[4.4]nonan-1-yl benzoate (**1d**) (**Figure S6**) S6

1.7 3,3-Dimethyl-1,4,5,7,8,8a-hexahydrocyclopenta[*c*]azepin-2(3*H*)-yl benzoate (**4d**) (**Figure S7**) S7

1.8 3,3-Dimethyl-3,4,6,7,8,8a-hexahydrocyclopenta[*c*]azepin-2(1*H*)-yl benzoate (**5d**) (**Figure S8**) S7

1.9 (5*R*(*S*),6*R*(*S*))-6-((Benzoyloxy)methyl)-2,2-dimethyl-1-azaspiro[4.4]nonan-1-oxyl (**13**) (**Figure S9**) S8

1.10 (5a*S*(*R*),8a*R*(*S*))-3,3,4-Trimethyloctahydro-1*H*-cyclopenta[2,3]azeto[1,2-*a*]pyrrol-4-ium iodide (**15**) (**Figure S10**) S8

1.11 2,3,3-Trimethyl-1,2,3,4,5,7,8,8a-octahydrocyclopenta[*c*]azepine (**16**) (**Figure S11**) S9

2. UV spectral data S9

2.1 (5*R*(*S*),6*R*(*S*))-6-((Benzoyloxy)methyl)-2,2-dimethyl-1-azaspiro[4.4]nonan-1-oxyl (**13**) (**Figure S12**) S9

3. ¹H and ¹³C NMR spectral data S10
3.1 (5a*S*(*R*),8a*R*(*S*))-3,3-Dimethyloctahydrocyclopenta[2,3]azeto[1,2-*a*]pyrrole (**3**) (**Figure S13, S14**) S10
3.2 (5a*S*(*R*),8a*R*(*S*))-3,3-Dimethyloctahydro-1*H*-cyclopenta[2,3]azeto[1,2-*a*]pyrrol-4-ium bromide (**3×HBr**) (**Figure S15, S16**) S11
3.3 ((5*R*(*S*),6*R*(*S*))-(1-(Benzyloxy)-2,2-dimethyl-1-azaspiro[4.4]nonan-6-yl)-methanol (**1c**) (**Figure S17, S18**) S12
3.4 2-(Benzyloxy)-3,3-dimethyl-1,2,3,4,5,7,8,8a-octahydrocyclopenta[*c*]azepine (**4c**) (**Figure S19, S20, S21, S22**) S13
3.5 2-(Benzyloxy)-3,3-dimethyl-1,2,3,4,6,7,8,8a-octahydrocyclopenta[*c*]azepine (**5c**) (**Figure S23, S24**) S15
3.6 (5*R*(*S*),6*R*(*S*))-6-(Hydroxymethyl)-2,2-dimethyl-1-azaspiro[4.4]nonan-1-yl benzoate (**1d**) (**Figure S25, S26**) S16
3.7 3,3-Dimethyl-1,4,5,7,8,8a-hexahydrocyclopenta[*c*]azepin-2(3*H*)-yl benzoate (**4d**) (**Figure S27, S28**) S17
3.8 3,3-Dimethyl-3,4,6,7,8,8a-hexahydrocyclopenta[c]azepin-2(1*H*)-yl benzoate (**5d**) (**Figure S29, S30**) S18
3.9 (5*R*(*S*),6*R*(*S*))-6-((Benzoyloxy)methyl)-2,2-dimethyl-1-azaspiro[4.4]nonan-1-ium 2,2,2-trifluoroacetate (**14**) (**Figure S31**) S19
3.10 (5a*S*(*R*),8a*R*(*S*))-3,3,4-Trimethyloctahydro-1*H*-cyclopenta[2,3]azeto[1,2-*a*]pyrrol-4-ium iodide (**15**) (**Figure S32, S33**) S19
3.11 2,3,3-Trimethyl-1,2,3,4,5,7,8,8a-octahydrocyclopenta[*c*]azepine (**16**) (**Figure S34, S35**) S20
4. 2D NMR spectral data S21
4.1 (5a*S*(*R*),8a*R*(*S*))-3,3-Dimethyloctahydrocyclopenta[2,3]azeto[1,2-*a*]pyrrole (**3**) (**Figure S36, S37, S38**) S21
4.2 (5a*S*(*R*),8a*R*(*S*))-3,3-Dimethyloctahydro-1*H*-cyclopenta[2,3]azeto[1,2-*a*]pyrrol-4-ium bromide (**3×HBr**) (**Figure S39, S40, S41**) S23
4.3 (2-(Benzyloxy)-3,3-dimethyl-1,2,3,4,5,7,8,8a-octahydrocyclopenta[*c*]azepine (**4c**) (**Figure S42, S43, S44**) S24
4.4 (2-(Benzyloxy)-3,3-dimethyl-1,2,3,4,6,7,8,8a-octahydrocyclopenta[*c*]azepine (**5c**) (**Figure S45, S46, S47**) S26
4.5 (3,3-Dimethyl-1,4,5,7,8,8*a*-hexahydrocyclopenta[*c*]azepin-2(3*H*)-yl benzoate (**4d**) (**Figure S48, S49, S50**) S27
4.6 (3,3-Dimethyl-3,4,6,7,8,8*a*-hexahydrocyclopenta[*c*]azepin-2(1*H*)-yl benzoate (**5d**) (**Figure S51, S52, S53**) S29
4.7 (2,3,3-Trimethyl-1,2,3,4,5,7,8,8a-octahydrocyclopenta[*c*]azepine (**16**) (**Figure S54, S55, S56**) S30
5. EPR spectral data S32
5.1 (5*R*(*S*),6*R*(*S*))-6-((Benzoyloxy)methyl)-2,2-dimethyl-1-azaspiro[4.4]nonan-1-oxyl (**13**) (**Figure S57**) S32

6. Gas chromatography data S32
6.1 Gas chromatography data of Hofmann elimination of salt **15** (reaction mass) (**Figure S58, Table S1**) S32
7. X-ray analysis data for compound 3×HBr S33
7.1 Experimental details for compound **3×HBr** S33
7.2 The structure and atom numbering of **3×HBr (Figure S59)** S33
8. NMR spectrum fine structure analysis S34
8.1 (5a*S*(*R*),8a*R*(*S*))-3,3-Dimethyloctahydro-1*H*-cyclopenta[2,3]azeto[1,2-*a*]pyrrol-4-ium bromide (**3×HBr**) (**Figure S60**) S34
8.2 Line shape analysis of multiplets for **3×HBr** (**Figure S61, Table S2)** S35
9. HPLC analysis S37
9.1 HPLC analysis of 2-(benzyloxy)-3,3-dimethyl-1,2,3,4,5,7,8,8a-octahydrocyclopenta[*c*]azepine (**4c**) and 2-(benzyloxy)-3,3-dimethyl-1,2,3,4,5,6,7,8-octahydrocyclopenta[*c*]azepine (**6c**) mixture and pure **4c** fraction (**Figure S62**) S37

1. IR spectral data

1.1 (5a*S*(*R*),8a*R*(*S*))-3,3-Dimethyloctahydrocyclopenta[2,3]azeto[1,2-*a*]pyrrole (**3**)

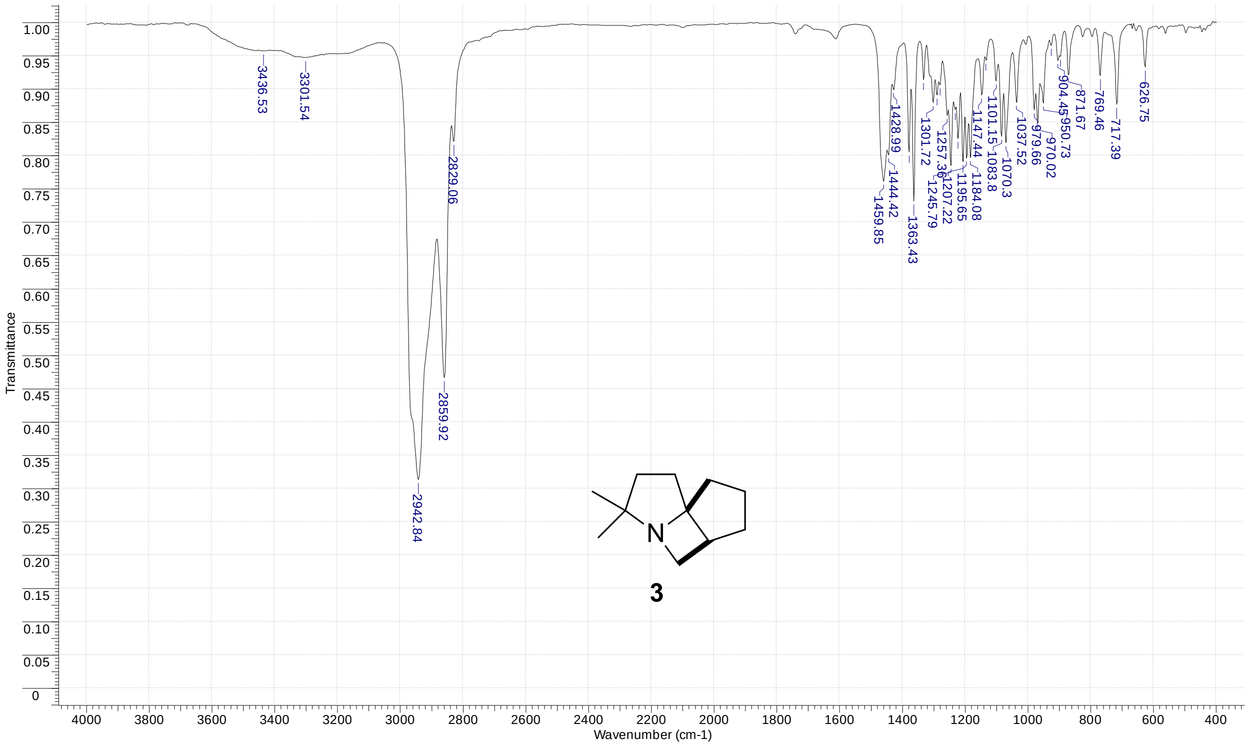

Figure S1. IR spectrum of **3** (neat)

1.2 (5a*S*(*R*),8a*R*(*S*))-3,3-Dimethyloctahydro-1*H*-cyclopenta[2,3]azeto[1,2-*a*]pyrrol-4-ium bromide (**3×HBr**)

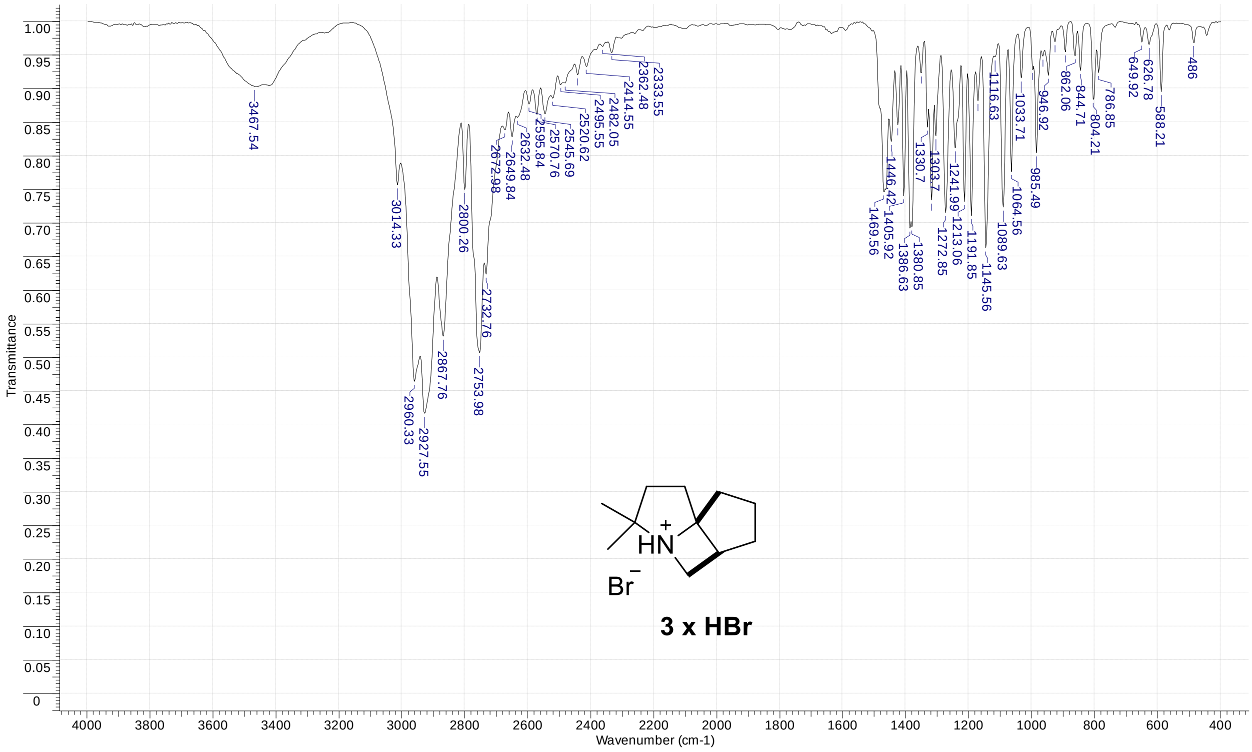

Figure S2. IR spectrum of **3×HBr** (KBr)

1.3 ((5*R*(*S*),6*R*(*S*)-1-(Benzyloxy)-2,2-dimethyl-1-azaspiro[4.4]nonan-6-yl)-methanol (**1c**)

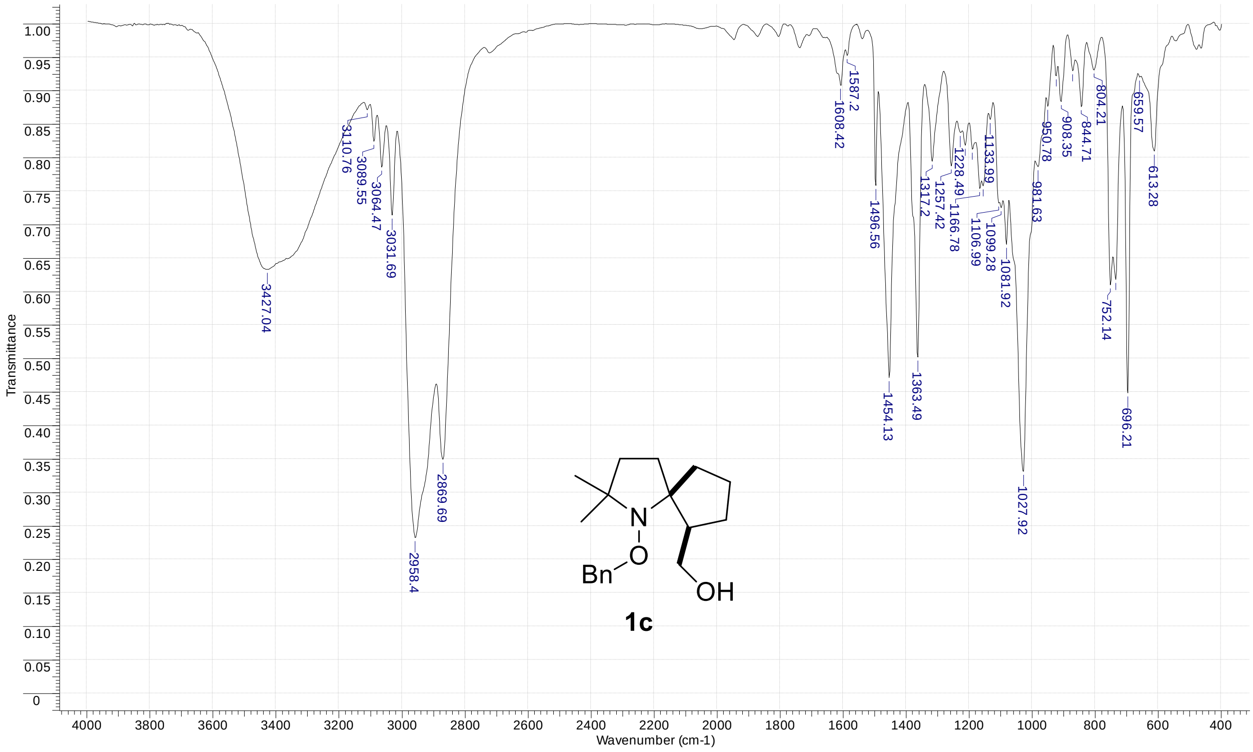

Figure S3. IR spectrum of **1c** (neat)

1.4 2-(Benzyloxy)-3,3-dimethyl-1,2,3,4,5,7,8,8a-octahydrocyclopenta[*c*]azepine (**4c**)

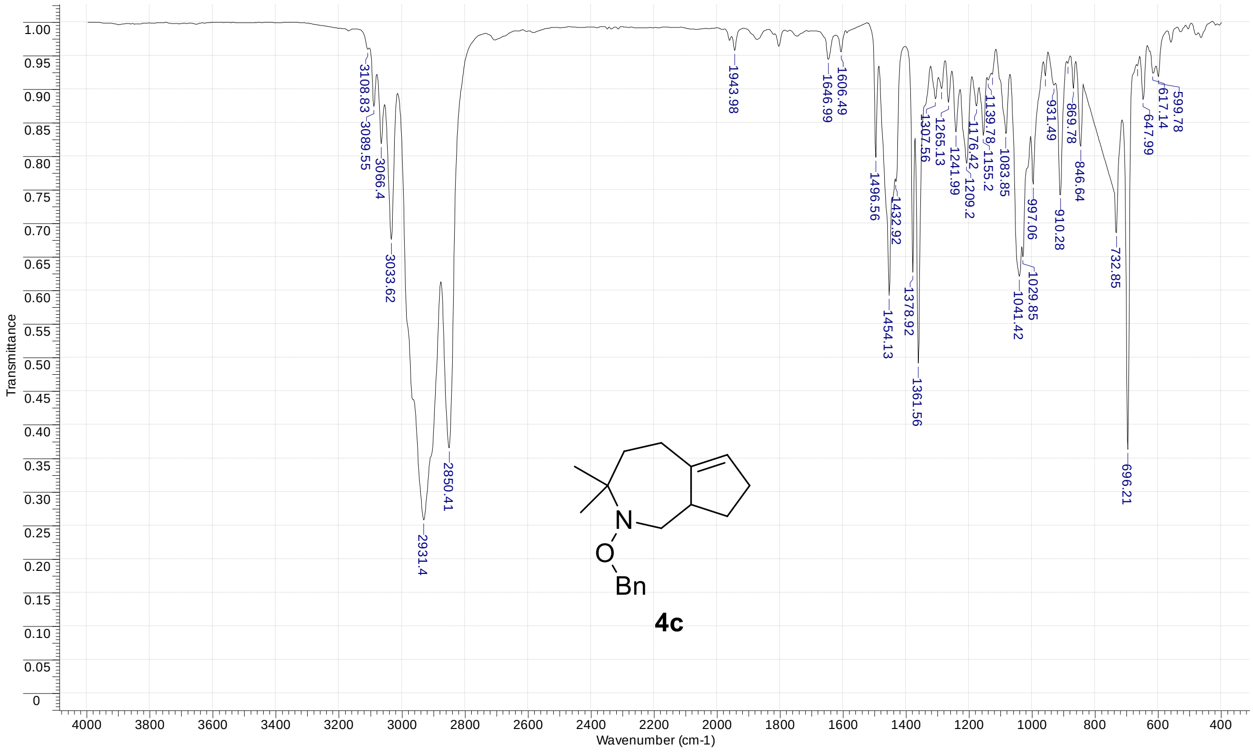

Figure S4. IR spectrum of **4c** (2% solution in CCl_4)

1.5 2-(Benzyloxy)-3,3-dimethyl-1,2,3,4,6,7,8,8a-octahydrocyclopenta[*c*]azepine (**5c**)

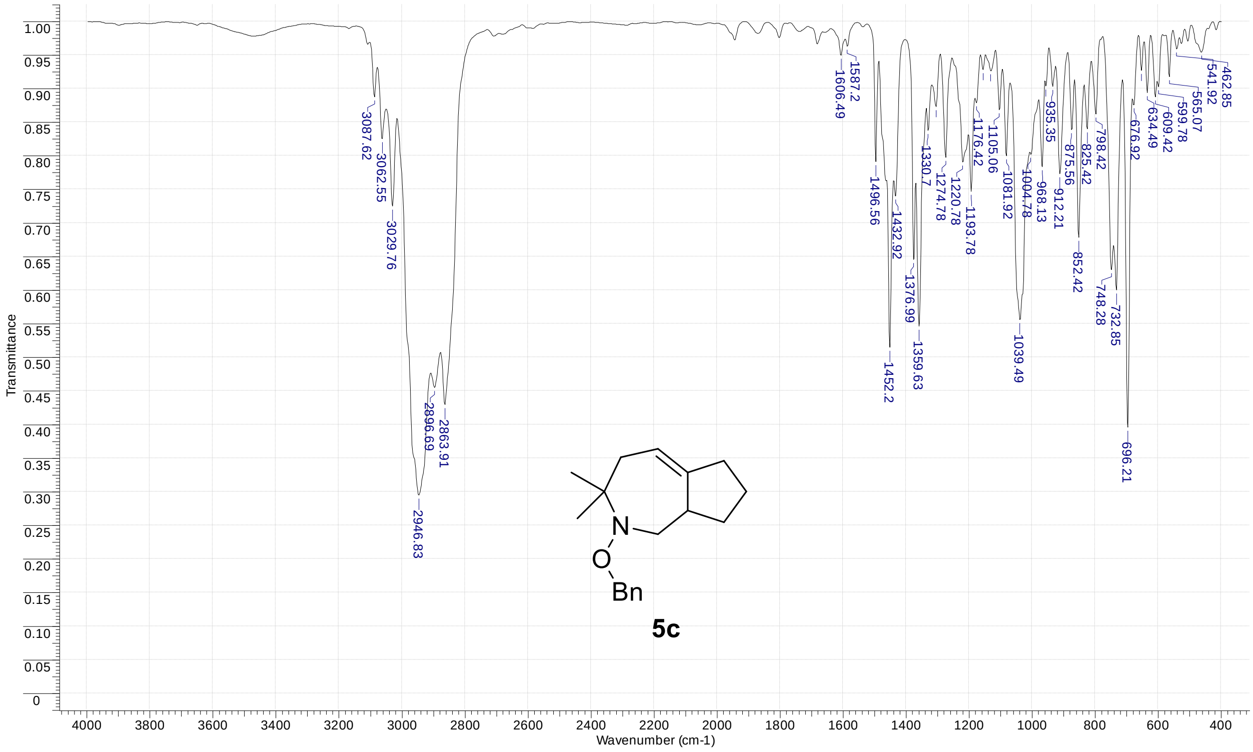

Figure S5. IR spectrum of **5c** (neat)

1.6 (5*R*(*S*),6*R*(*S*))-6-(Hydroxymethyl)-2,2-dimethyl-1-azaspiro[4.4]nonan-1-yl benzoate (**1d**)

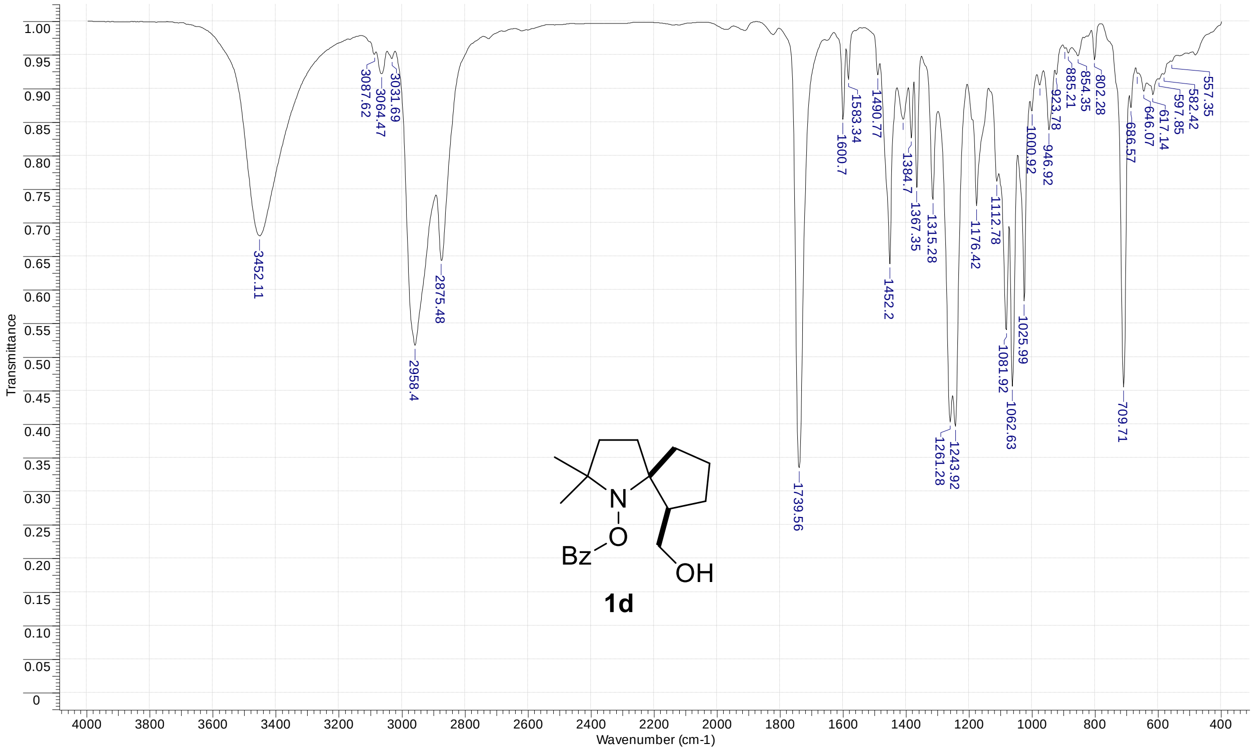

Figure S6. IR spectrum of **1d** (neat)

1.7 3,3-Dimethyl-1,4,5,7,8,8a-hexahydrocyclopenta[*c*]azepin-2(3*H*)-yl benzoate (**4d**)

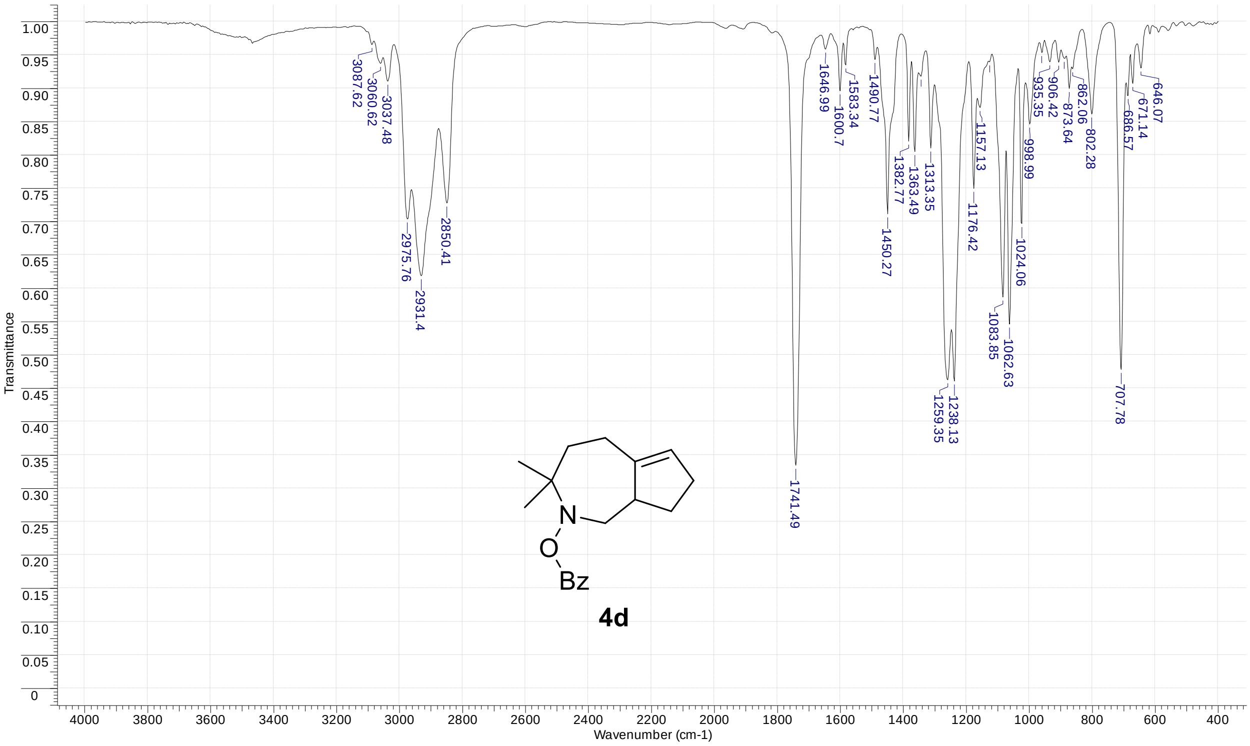

Figure S7. IR spectrum of **4d** (neat)

1.8 3,3-Dimethyl-3,4,6,7,8,8a-hexahydrocyclopenta[*c*]azepin-2(1*H*)-yl benzoate (**5d**)

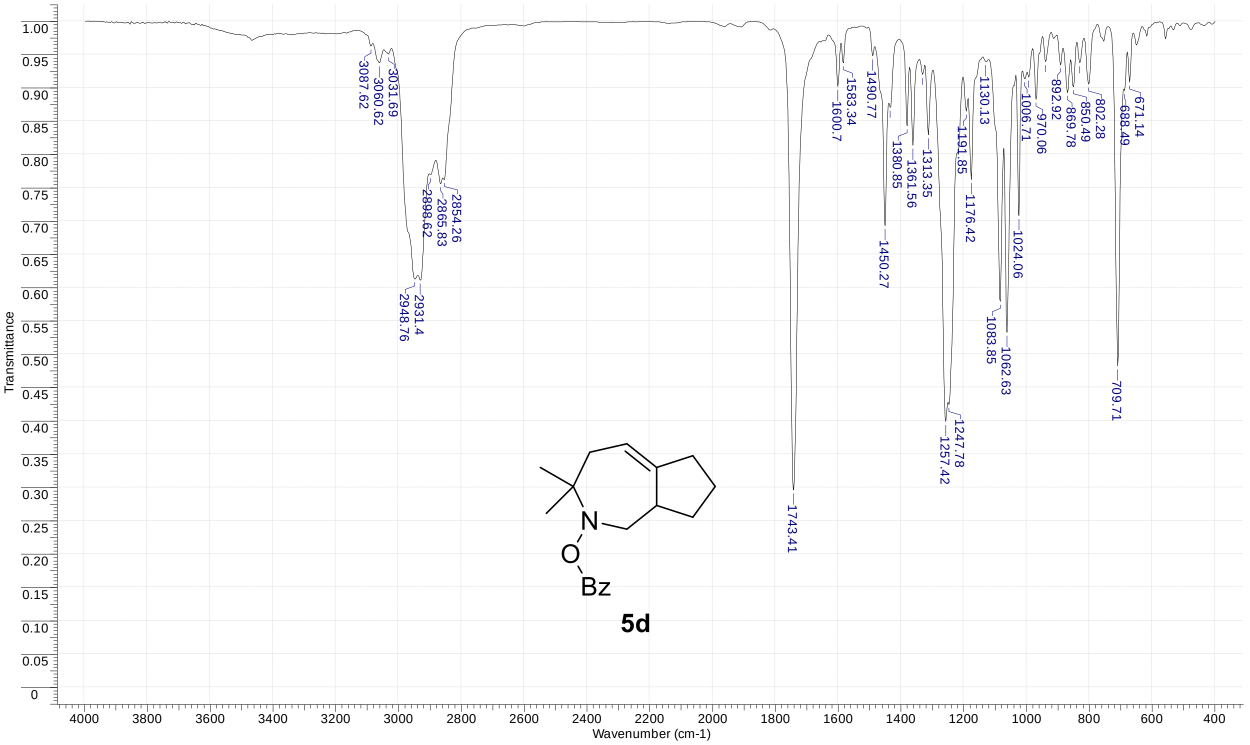

Figure S8. IR spectrum of **5d** (neat)

1.9 (5*R*(*S*),6*R*(*S*))-6-((Benzoyloxy)methyl)-2,2-dimethyl-1-azaspiro[4.4]nonan-1-oxyl (**13**)

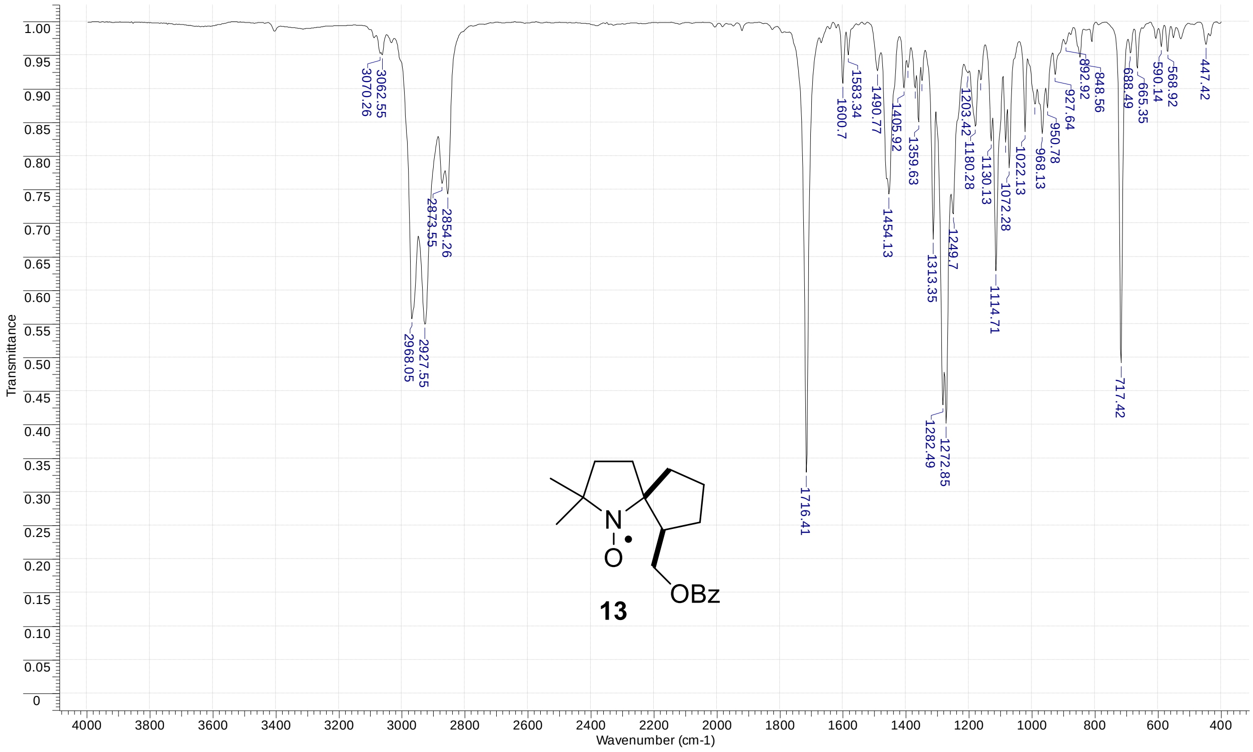

Figure S9. IR spectrum of **13** (KBr)

1.10 (5a*S*(*R*),8a*R*(*S*))-3,3,4-Trimethyloctahydro-1*H*-cyclopenta[2,3]azeto[1,2-*a*]pyrrol-4-ium iodide (**15**)

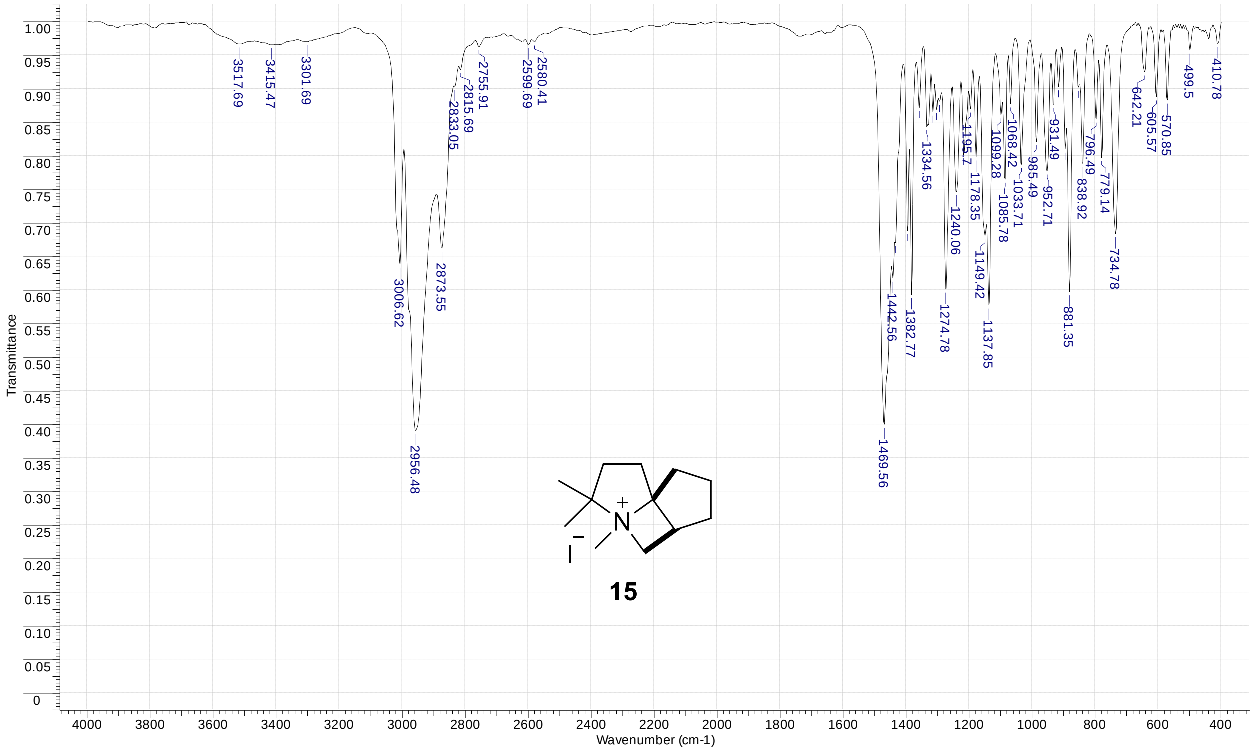

Figure S10. IR spectrum of **15** (KBr)

1.11 2,3,3-Trimethyl-1,2,3,4,5,7,8,8a-octahydrocyclopenta[*c*]azepine (**16**)

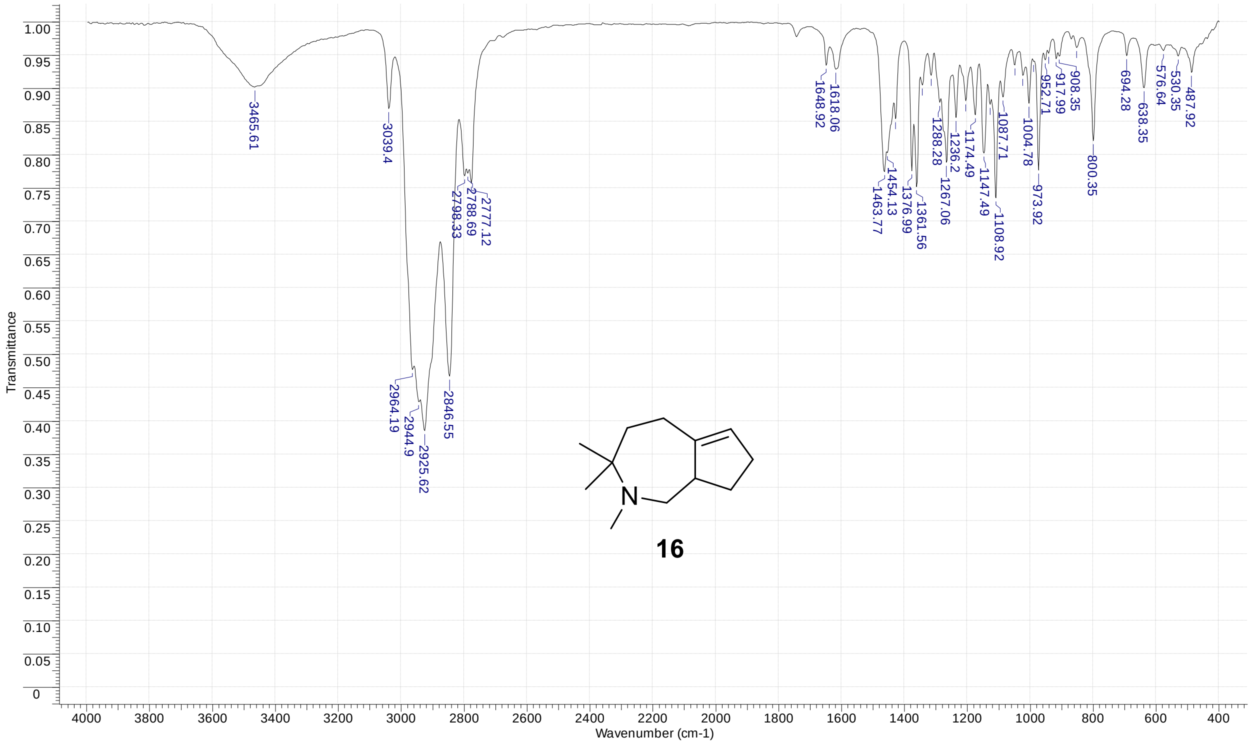

Figure S11. IR spectrum of **16** (neat)

2. UV spectral data

2.1 (5*R*(*S*),6*R*(*S*))-6-((Benzoyloxy)methyl)-2,2-dimethyl-1-azaspiro[4.4]nonan-1-oxyl (**13**)

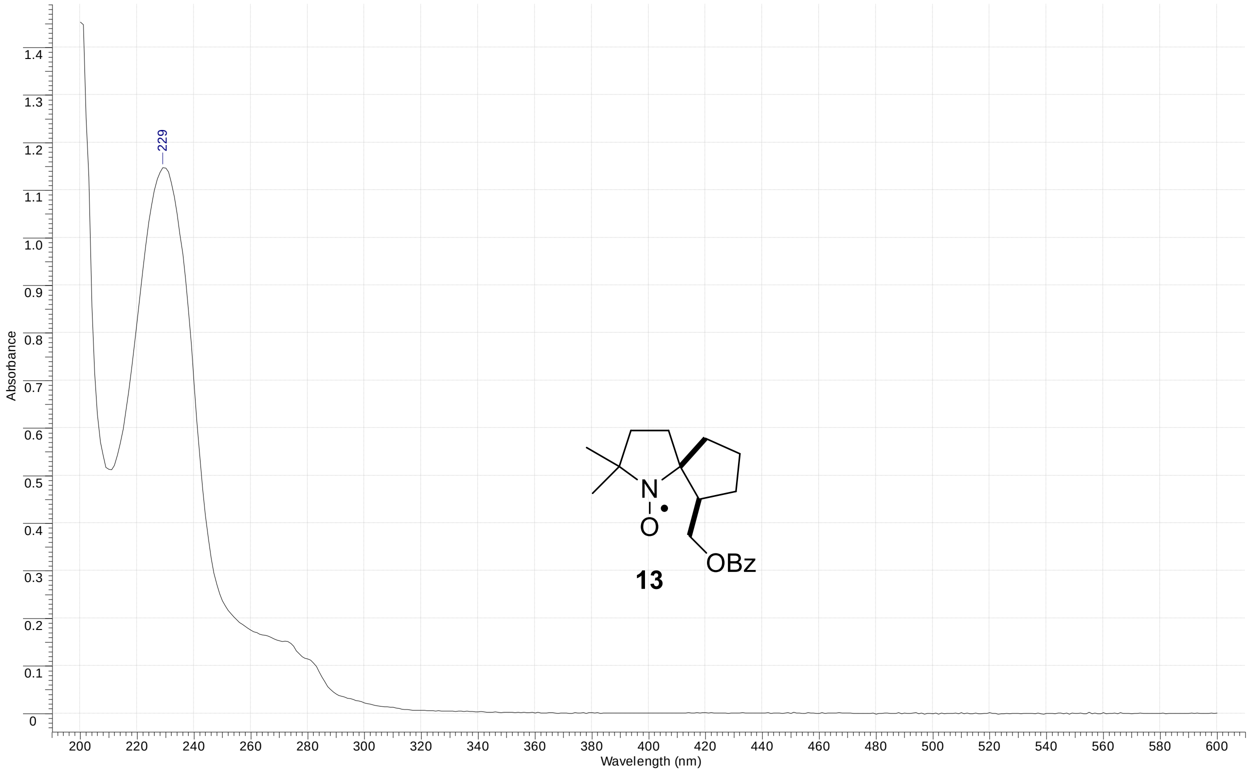

Figure S12. UV spectrum of **13** in EtOH (0.757mg/25ml, L=1cm)

3. ^{1}H and ^{13}C NMR spectral data

3.1 (5a*S*(*R*),8a*R*(*S*))-3,3-Dimethyloctahydrocyclopenta[2,3]azeto[1,2-*a*]pyrrole (**3**)

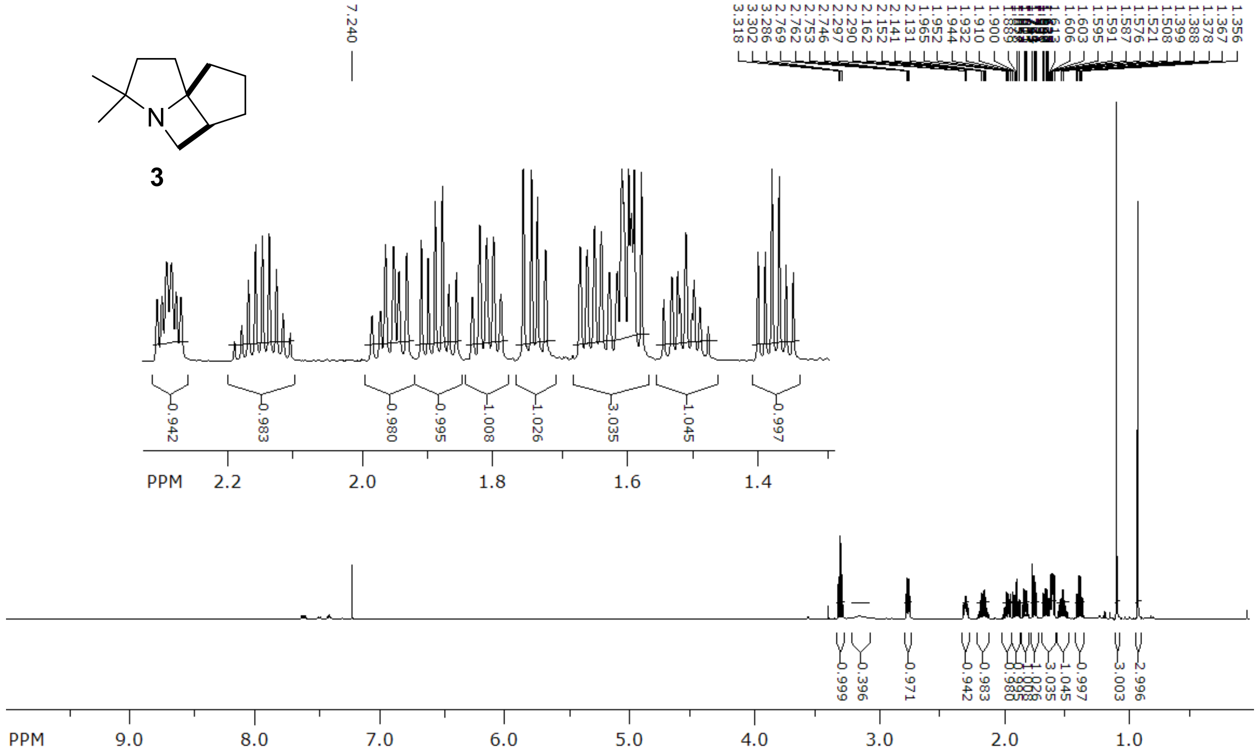

Figure S13. ^{1}H NMR spectrum of **3** in $CDCl_3$ at 600 MHz

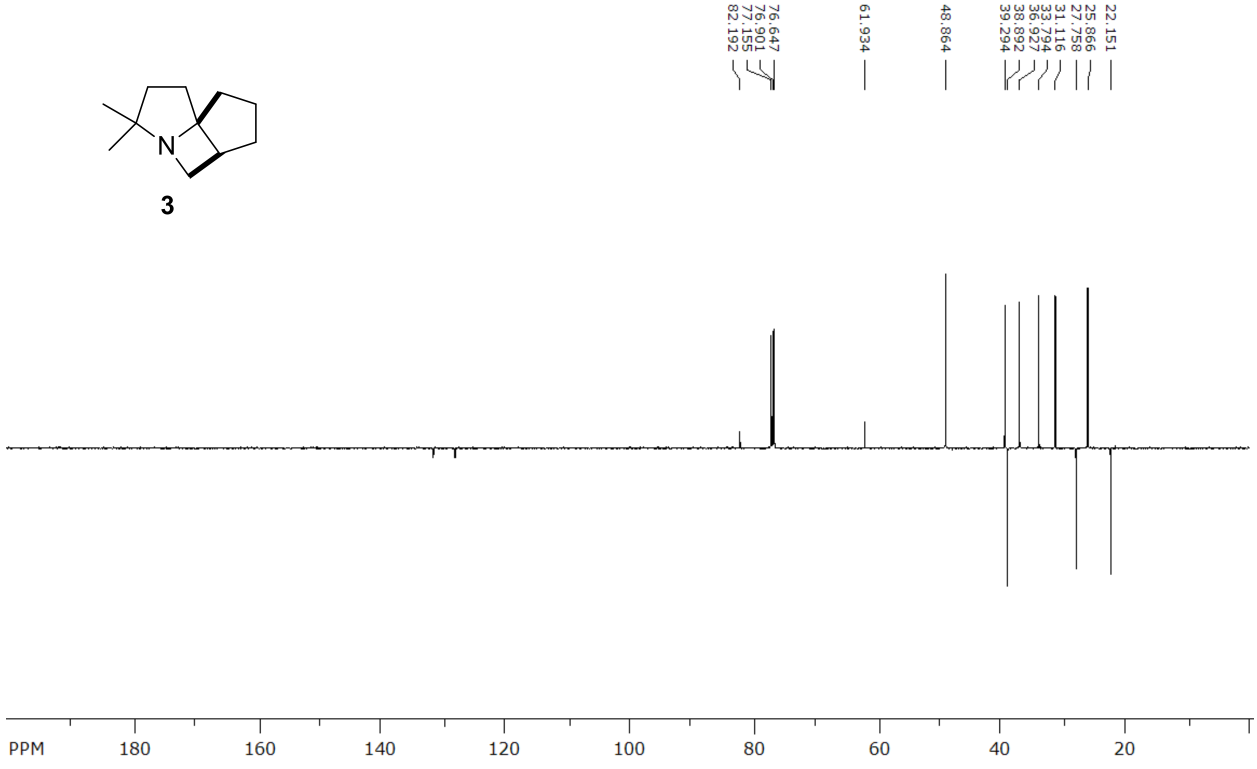

Figure S14. ^{13}C NMR spectrum of **3** in $CDCl_3$ at 125 MHz

3.2 (5a*S*(*R*),8a*R*(*S*))-3,3-Dimethyloctahydro-1*H*-cyclopenta[2,3]azeto[1,2-*a*]pyrrol-4-ium bromide (**3×HBr**)

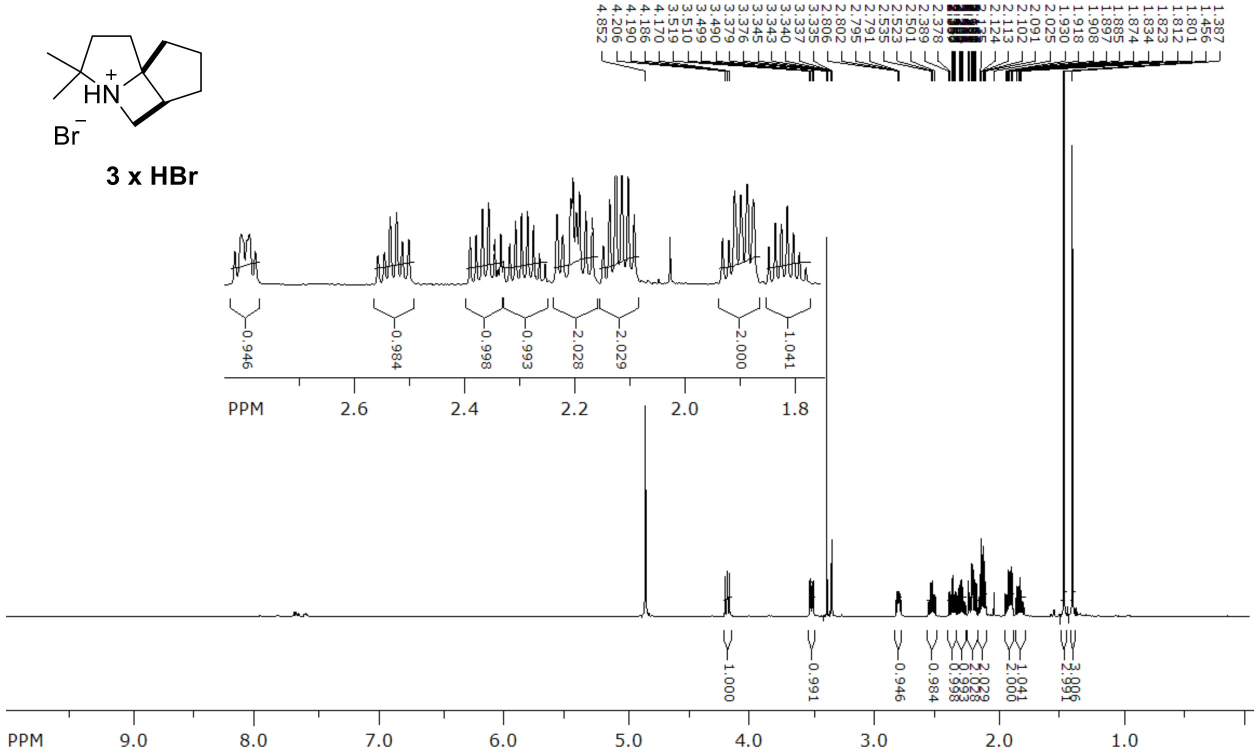

Figure S15. ^{1}H NMR spectrum of **3×HBr** in CD_3OD at 600 MHz

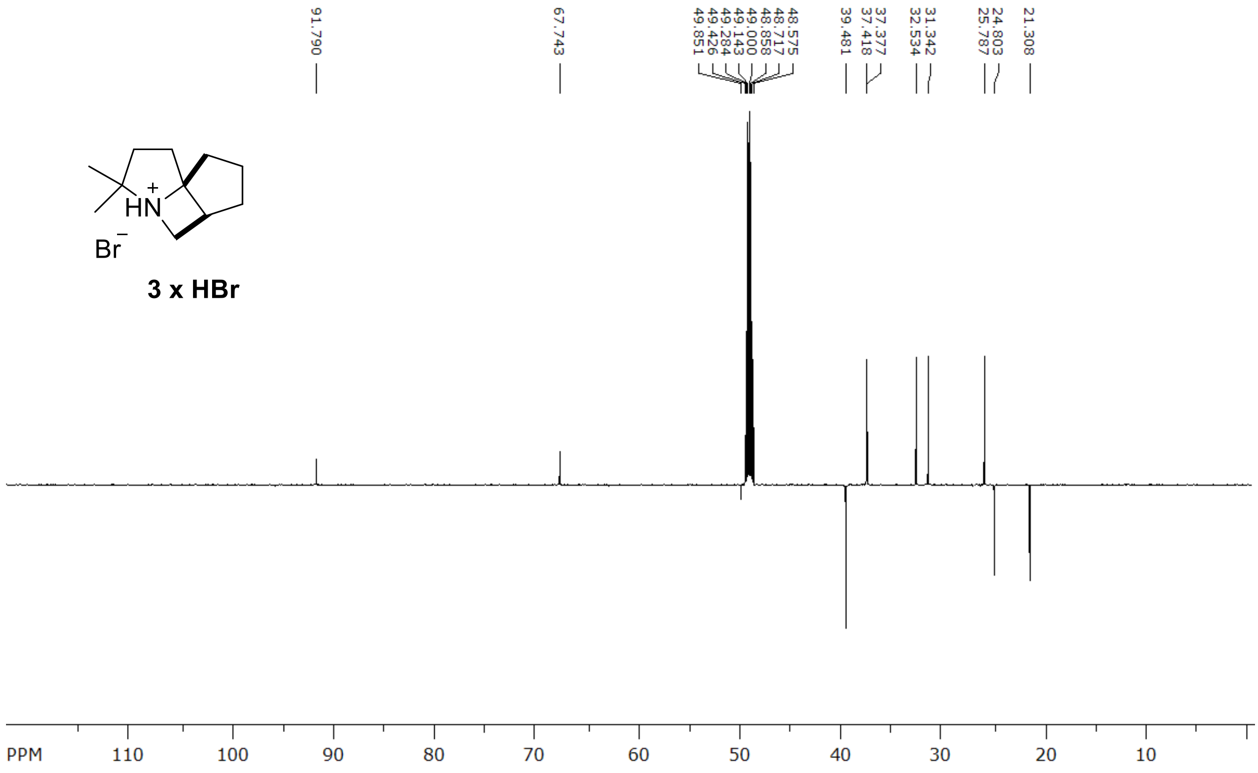

Figure S16. ^{13}C NMR spectrum of **3*HBr** in CD_3OD at 150 MHz

3.3 ((5*R*(*S*),6*R*(*S*))-(1-(Benzyloxy)-2,2-dimethyl-1-azaspiro[4.4]nonan-6-yl)-methanol (**1c**)

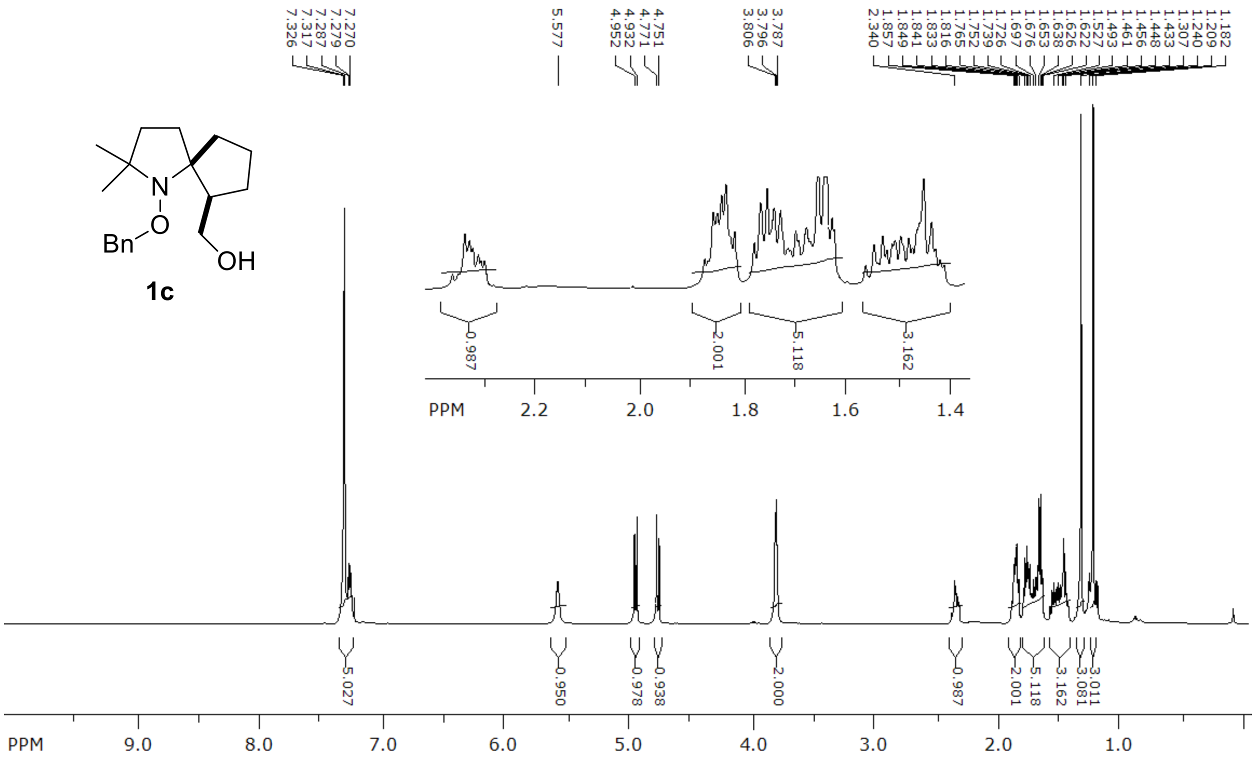

Figure S17. ^{1}H NMR spectrum of **1c** in $CDCl_3$ at 500 MHz

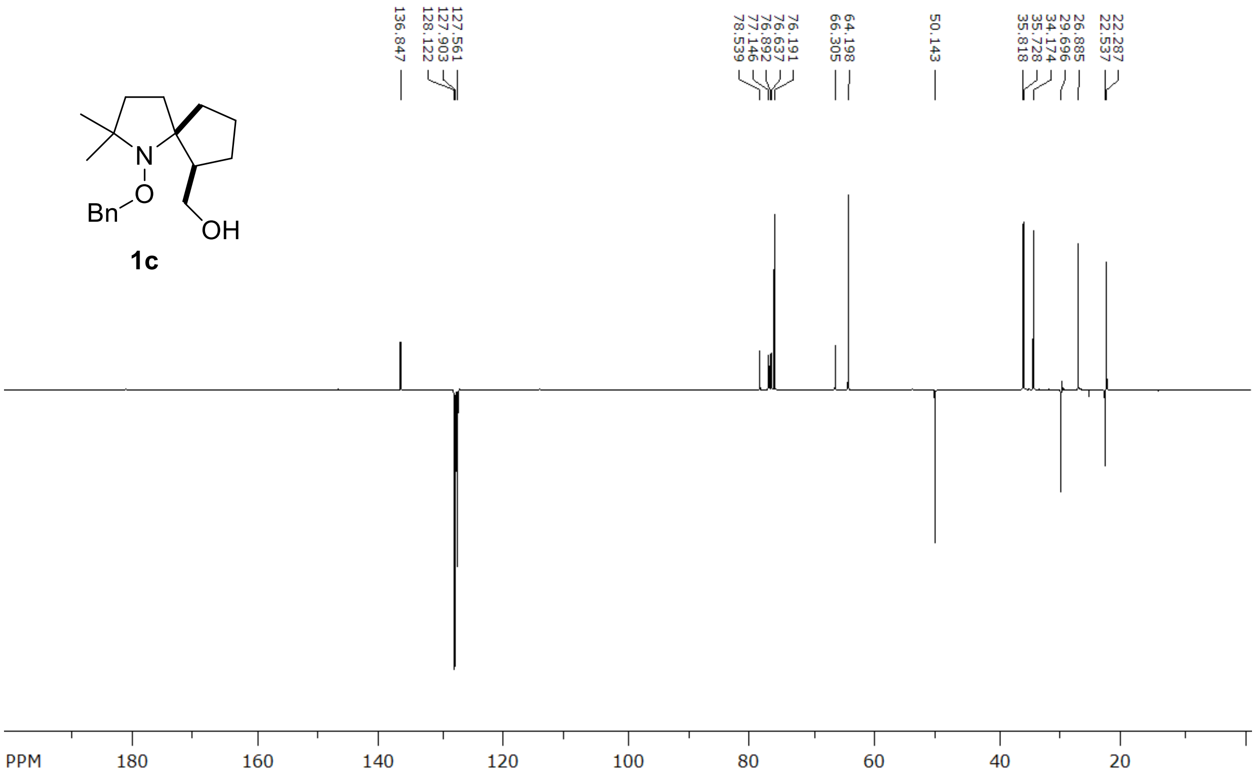

Figure S18. ^{13}C NMR spectrum of **1c** in $CDCl_3$ at 125 MHz

3.4 2-(Benzyloxy)-3,3-dimethyl-1,2,3,4,5,7,8,8a-octahydrocyclopenta[*c*]azepine (**4c**)

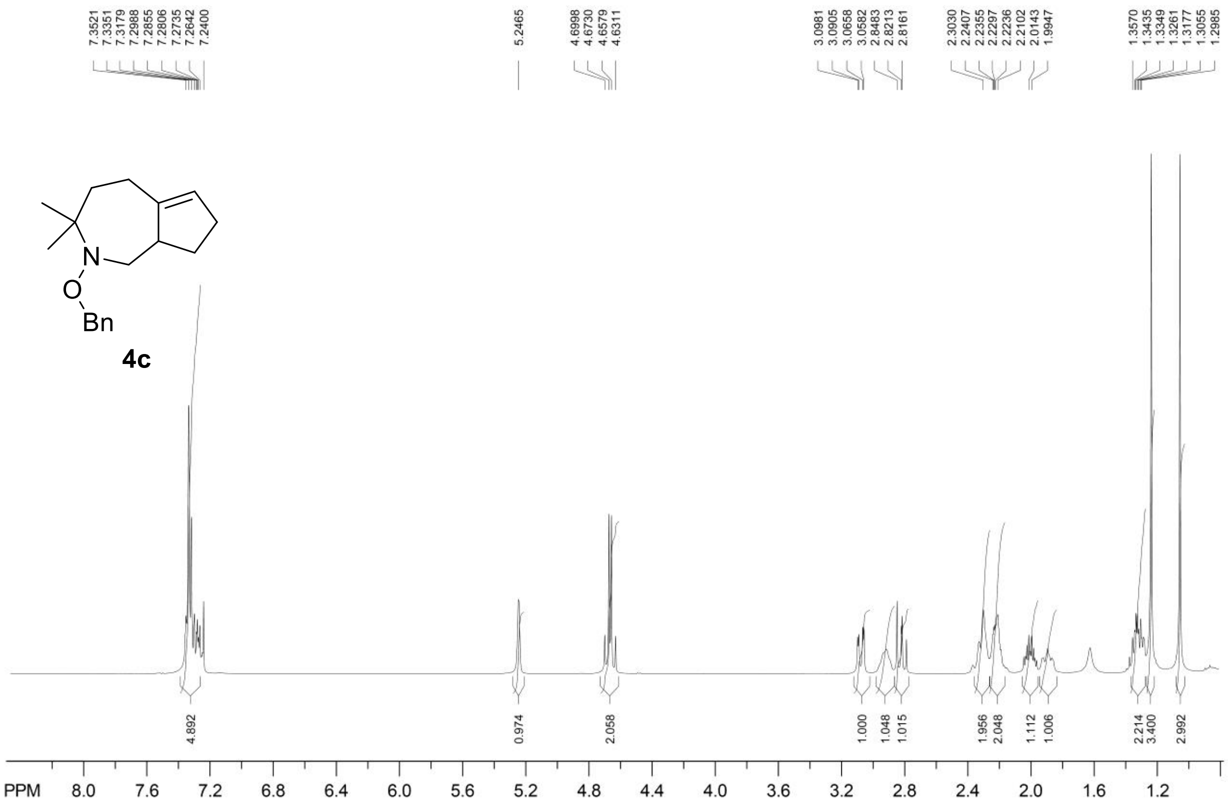

Figure S19. ^{1}H NMR spectrum of **4c** in $CDCl_3$ at 400 MHz

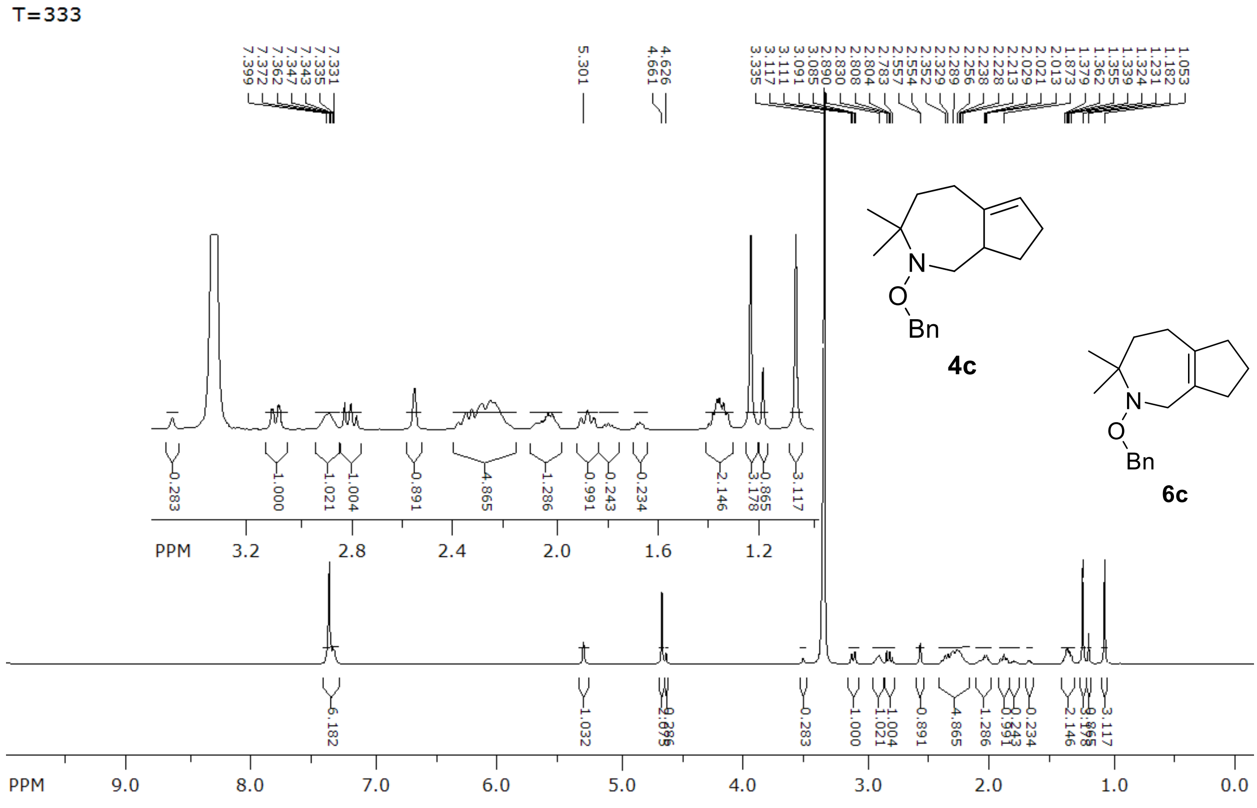

Figure S20. ^{1}H NMR spectrum of **4c** and **6c** mixture in DMSO-d_6 at 500 MHz (T=333 K)

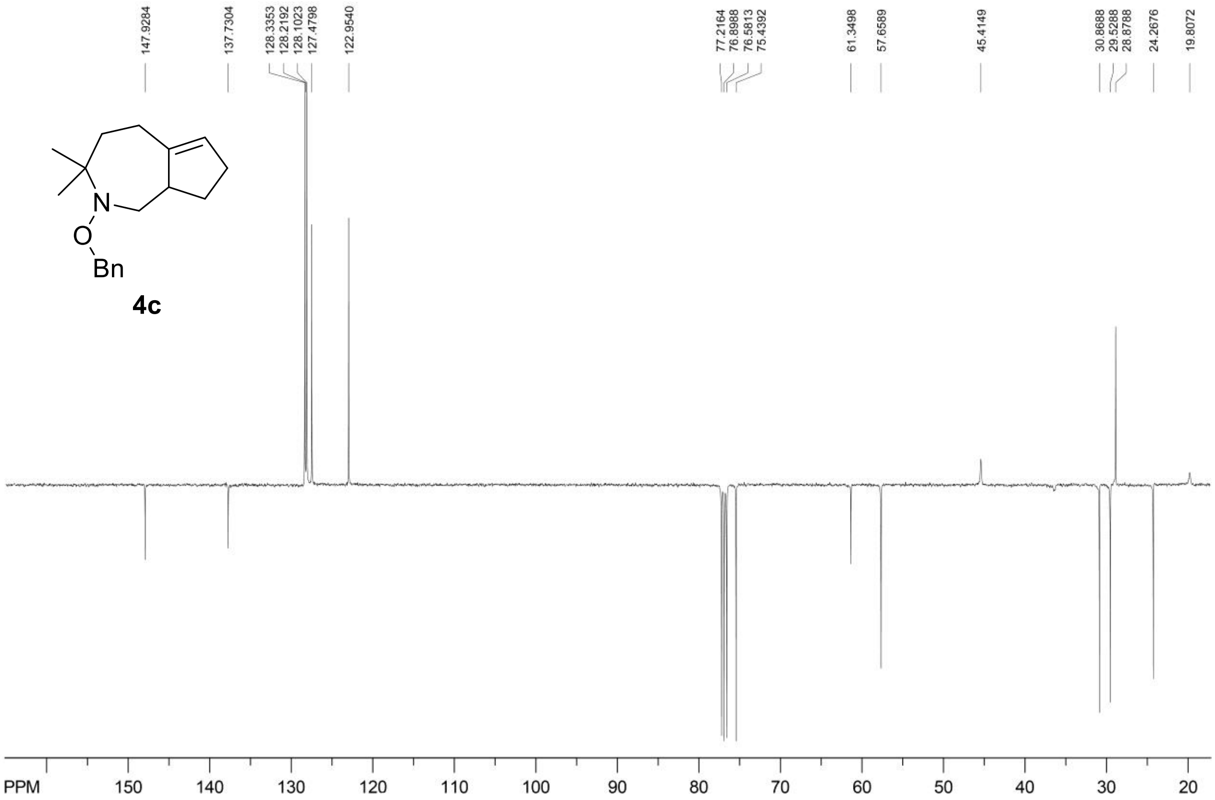

Figure S21. ^{13}C NMR spectrum of **4c** in $CDCl_3$ at 100 MHz

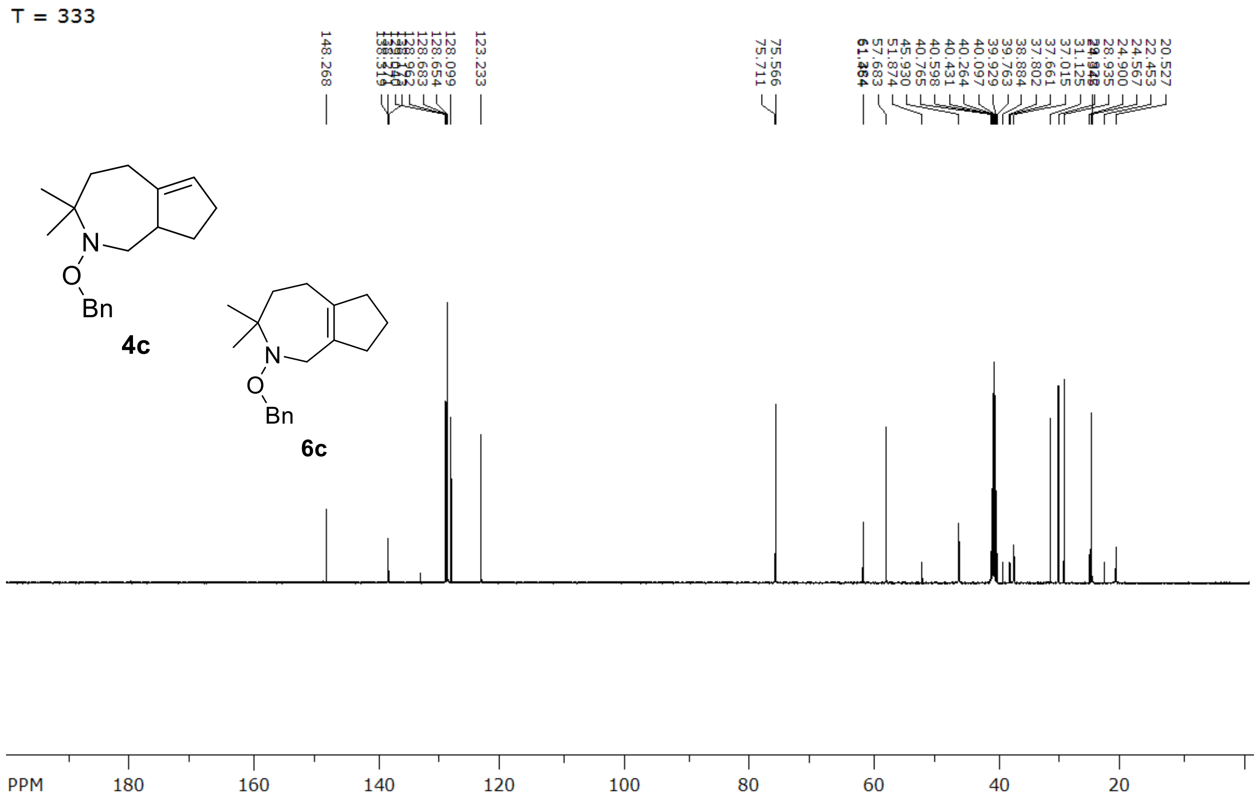

Figure S22. ^{13}C NMR spectrum of **4c** and **6c** mixture in DMSO-d_6 at 125 MHz (T=333 K)

3.5 2-(Benzyloxy)-3,3-dimethyl-1,2,3,4,6,7,8,8a-octahydrocyclopenta[*c*]azepine (**5c**)

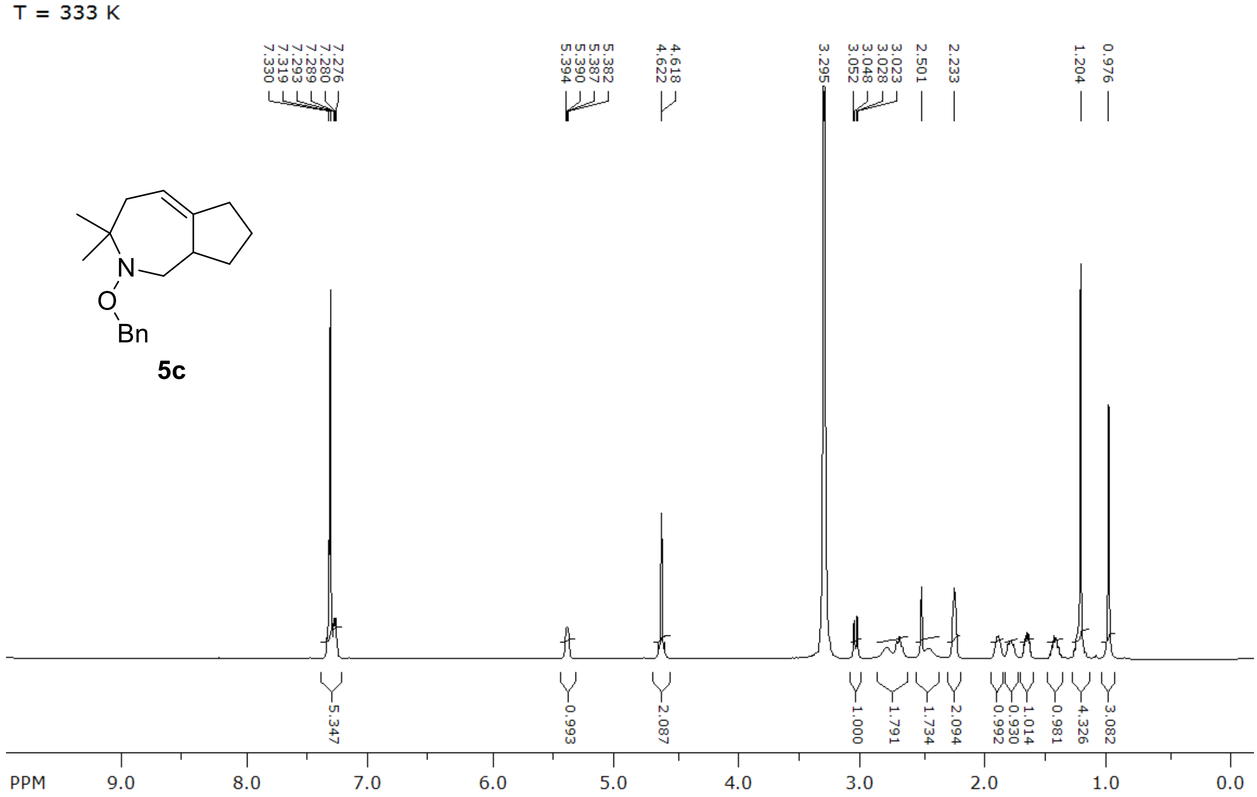

Figure S23. ^{1}H NMR spectrum of **5c** in DMSO-d_6 at 500 MHz (T=333 K)

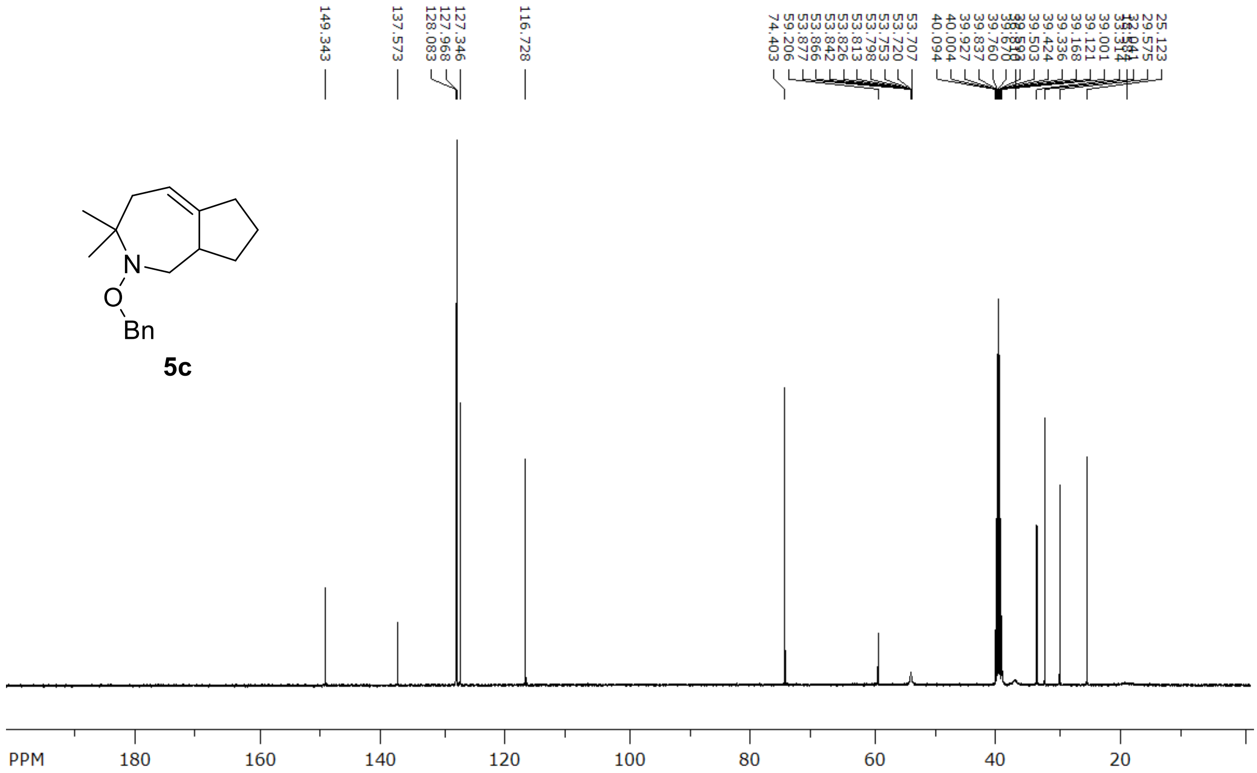

Figure S24. ^{13}C NMR spectrum of **5c** in DMSO-d_6 at 125 MHz

3.6 (5*R*(*S*),6*R*(*S*))-6-(Hydroxymethyl)-2,2-dimethyl-1-azaspiro[4.4]nonan-1-yl benzoate (**1d**)

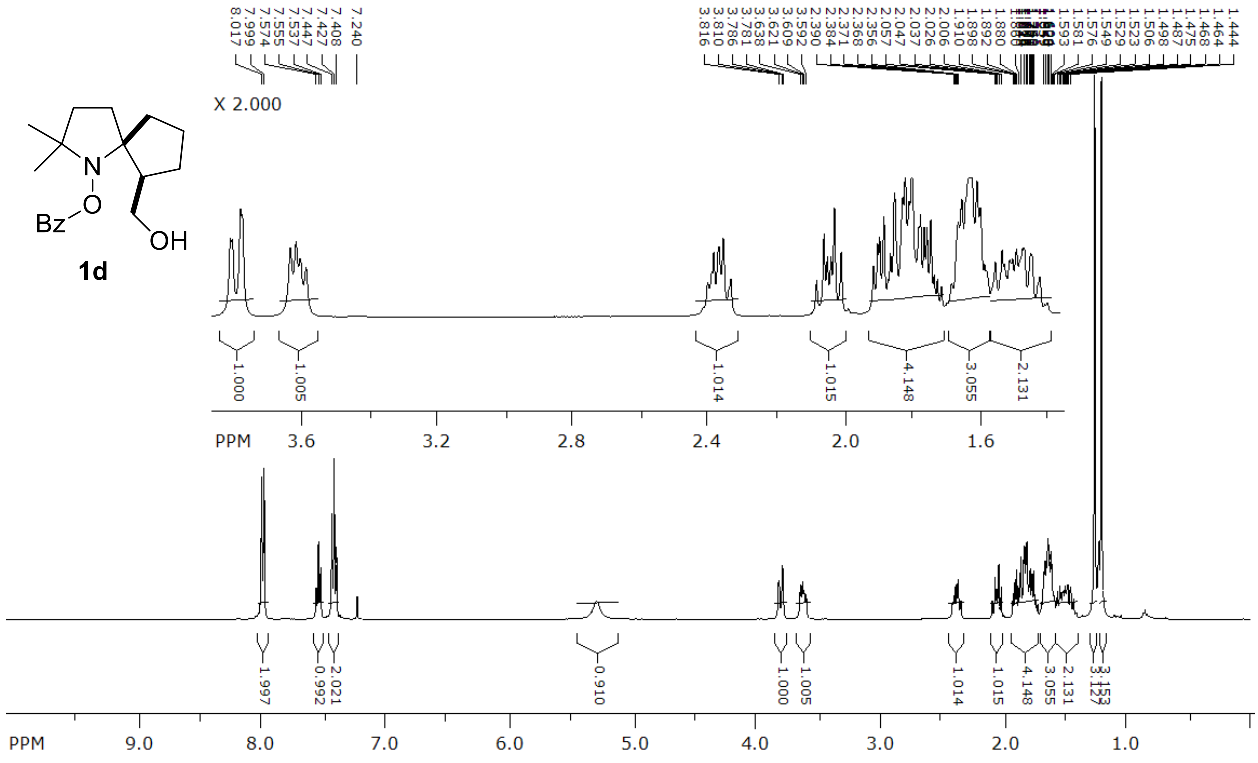

Figure S25. ^{1}H NMR spectrum of **1d** in $CDCl_3$ at 400 MHz

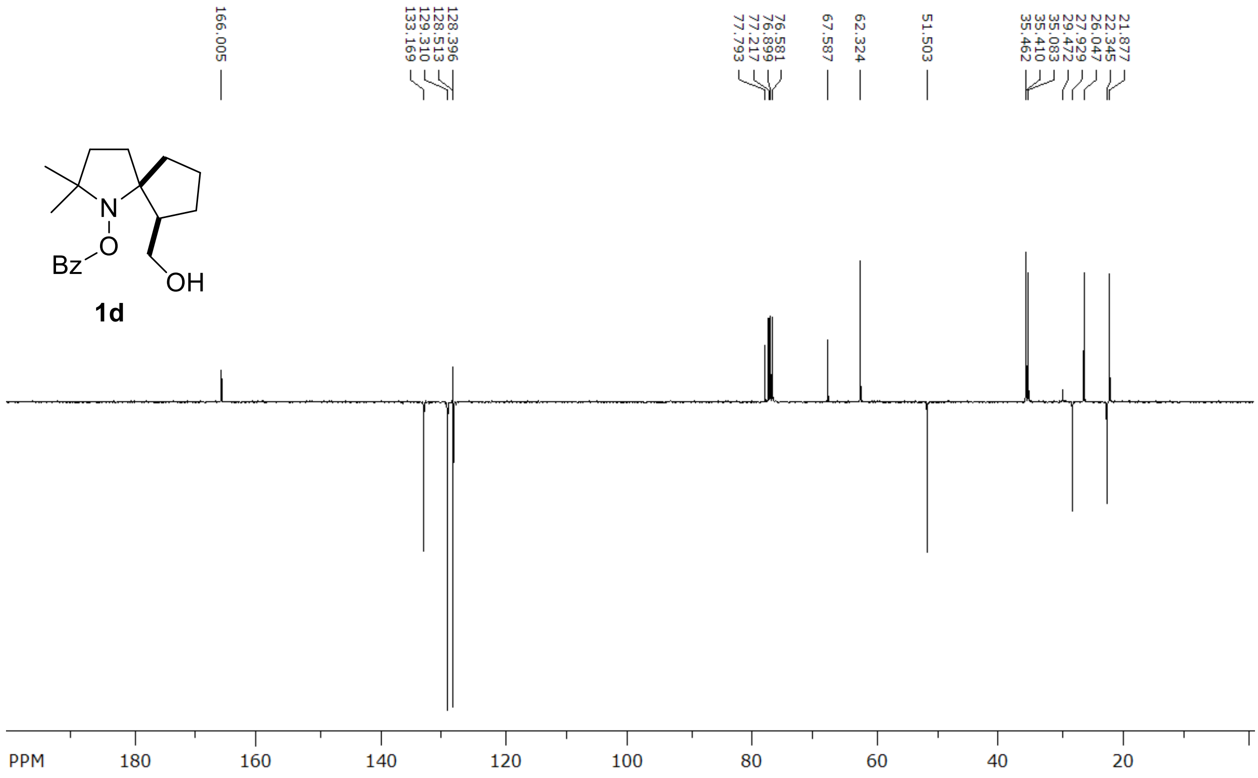

Figure S26. ^{13}C NMR spectrum of **1d** in $CDCl_3$ at 100 MHz

3.7 3,3-Dimethyl-1,4,5,7,8,8a-hexahydrocyclopenta[*c*]azepin-2(3*H*)-yl benzoate (**4d**)

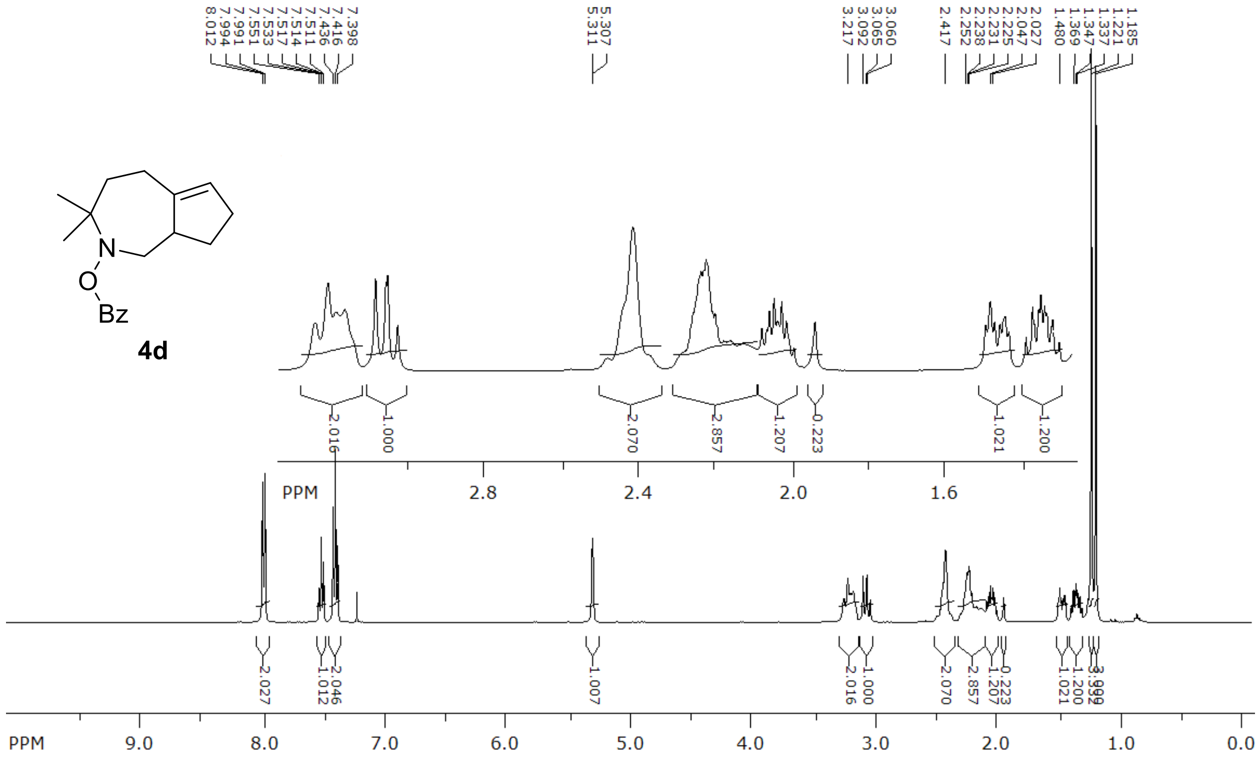

Figure S27. ^{1}H NMR spectrum of **4d** in $CDCl_3$ at 400 MHz

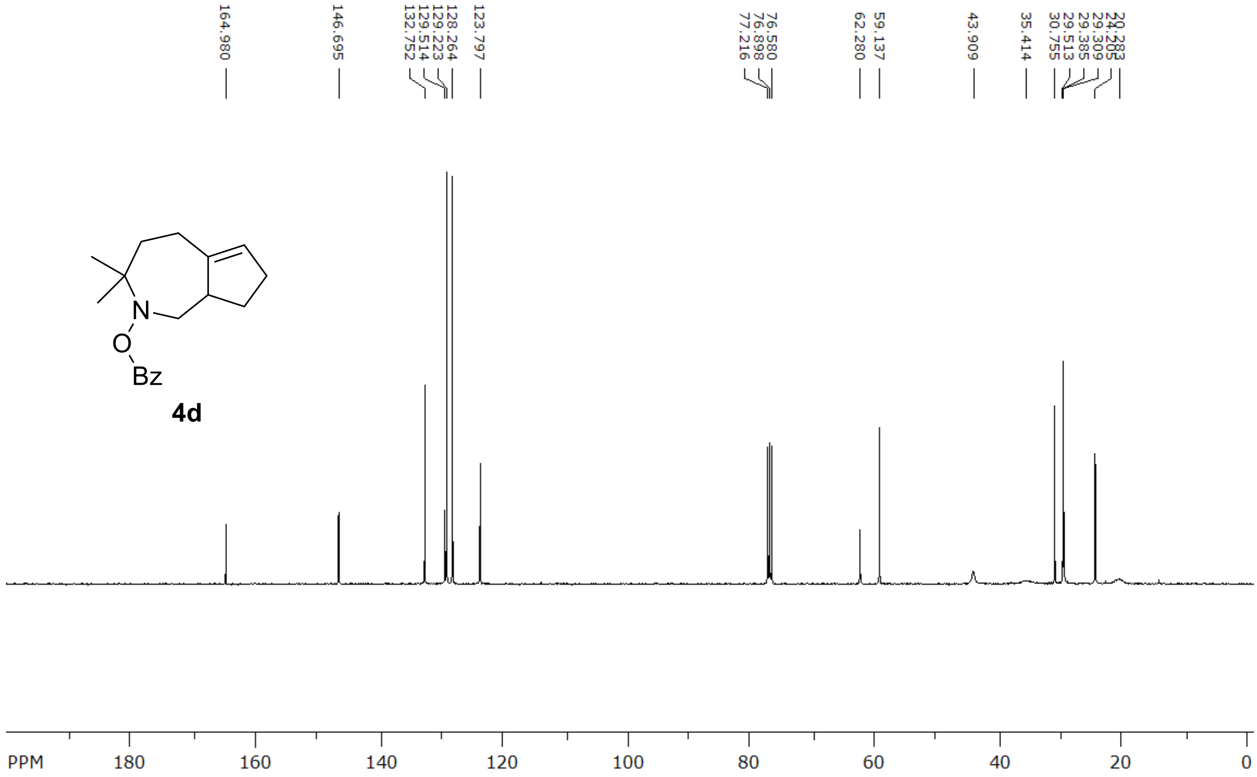

Figure S28. ^{13}C NMR spectrum of **4d** in $CDCl_3$ at 100 MHz

3.8 3,3-Dimethyl-3,4,6,7,8,8a-hexahydrocyclopenta[c]azepin-2(1*H*)-yl benzoate (**5d**)

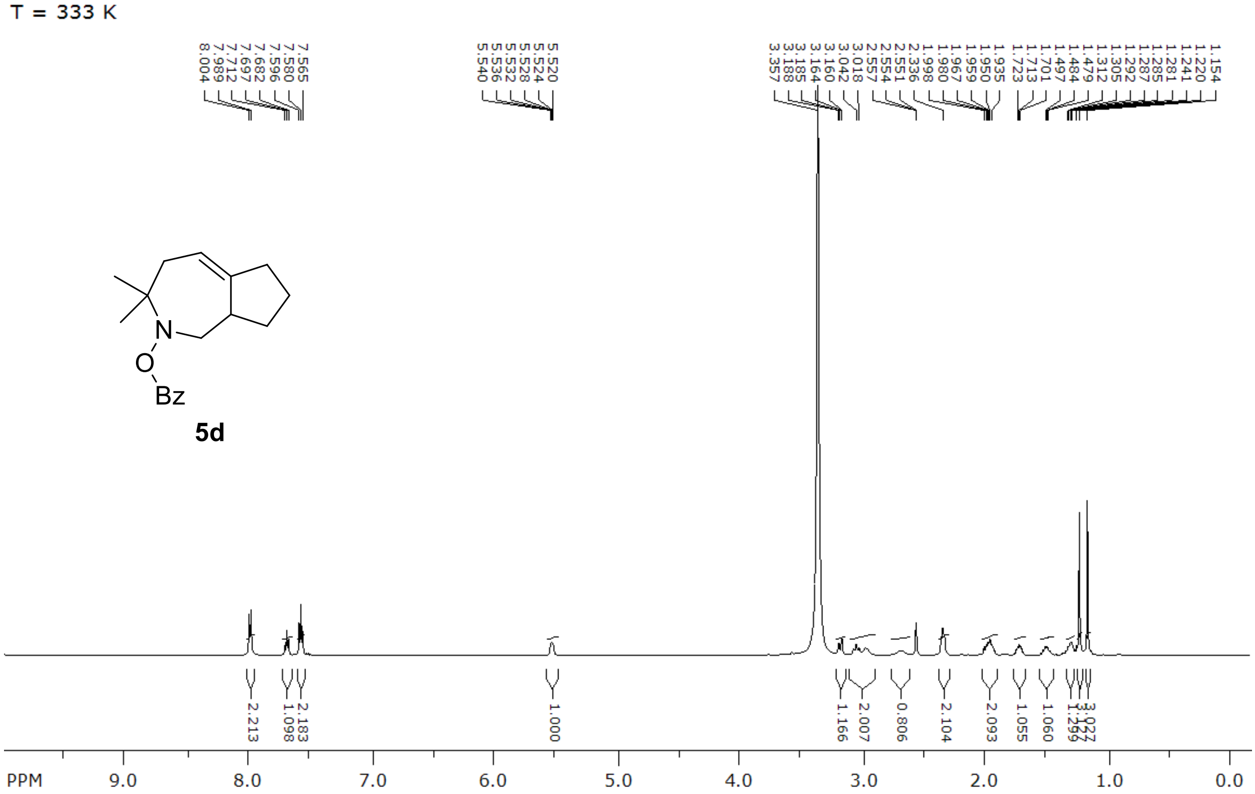

Figure S29. ^{1}H NMR spectrum of **5d** in DMSO-d_6 at 500 MHz (T=333 K)

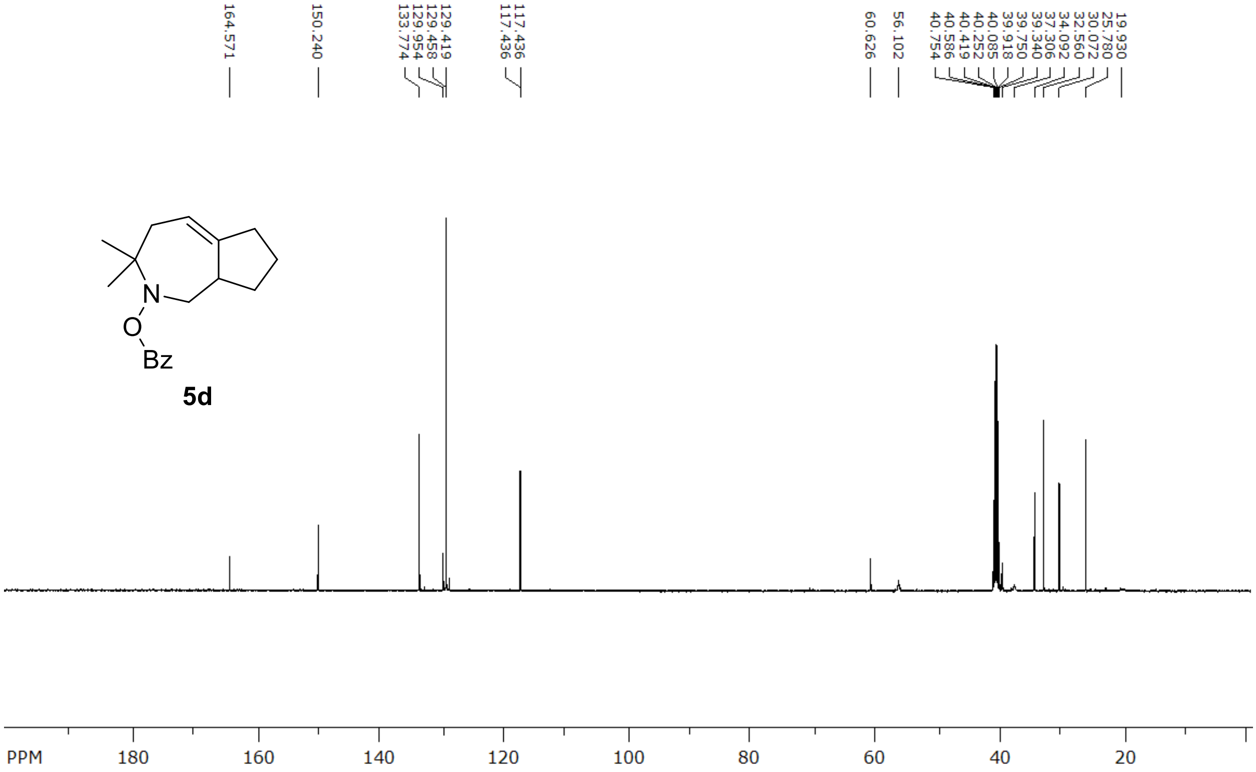

Figure S30. ^{13}C NMR spectrum of **5d** in DMSO-d_6 at 125 MHz (T=333 K)

3.9 (5*R*(*S*),6*R*(*S*))-6-((Benzoyloxy)methyl)-2,2-dimethyl-1-azaspiro[4.4]nonan-1-ium 2,2,2-trifluoroacetate (**14**)

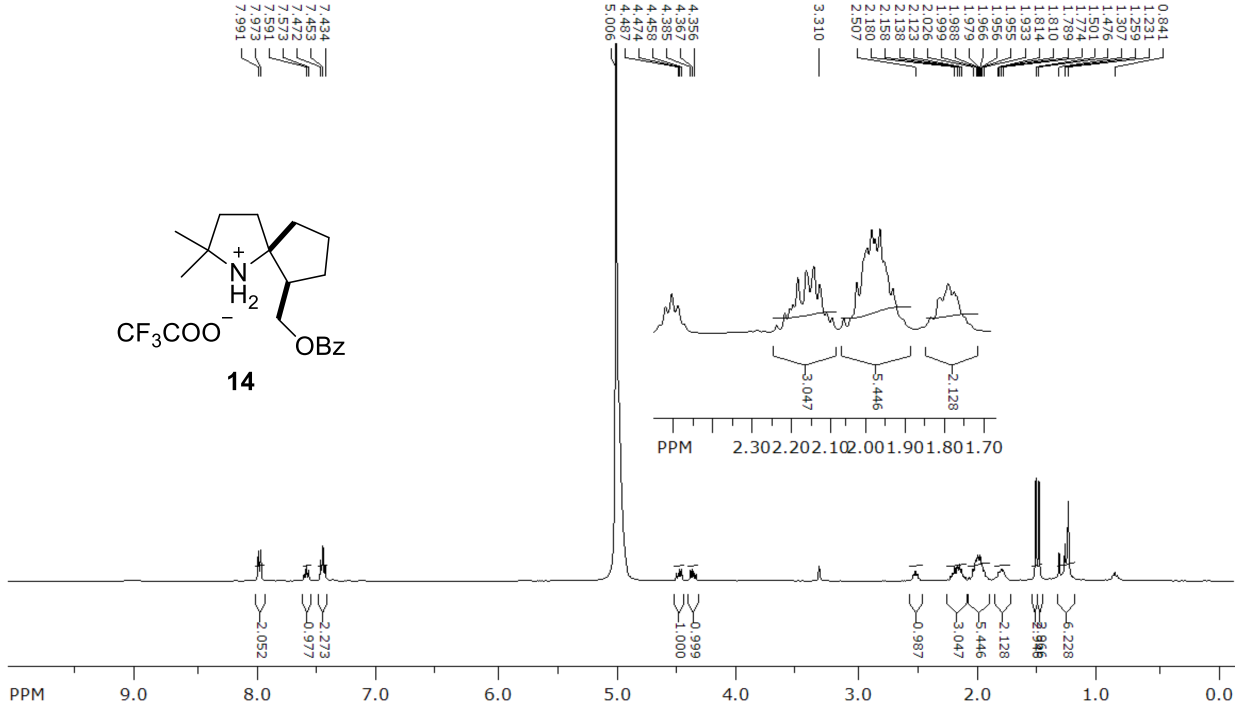

Figure S31. ^{1}H NMR spectrum of **14** in $CDCl_3$-CD_3OD-CF_3COOH mixture at 400 MHz

3.10 (5a*S*(*R*),8a*R*(*S*))-3,3,4-Trimethyloctahydro-1*H*-cyclopenta[2,3]azeto[1,2-*a*]pyrrol-4-ium iodide (**15**)

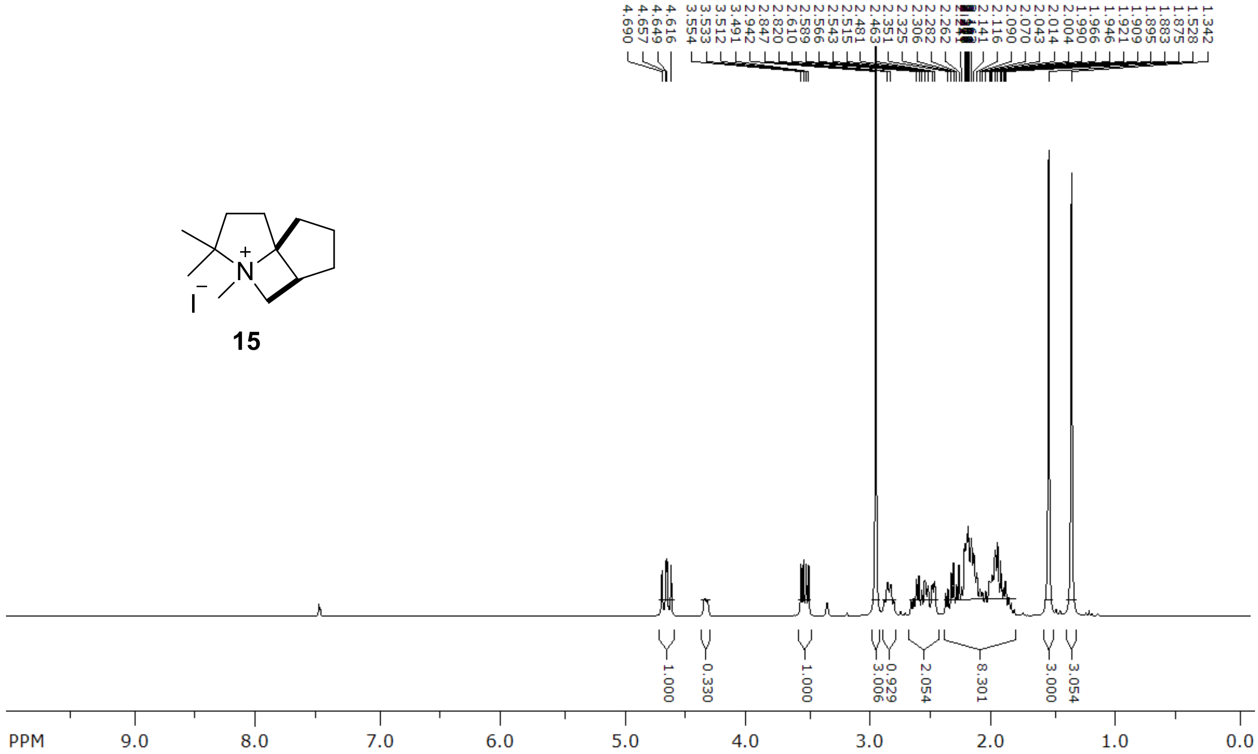

Figure S32. ^{1}H NMR spectrum of **15** in $CDCl_3$-CD_3OD mixture at 300 MHz

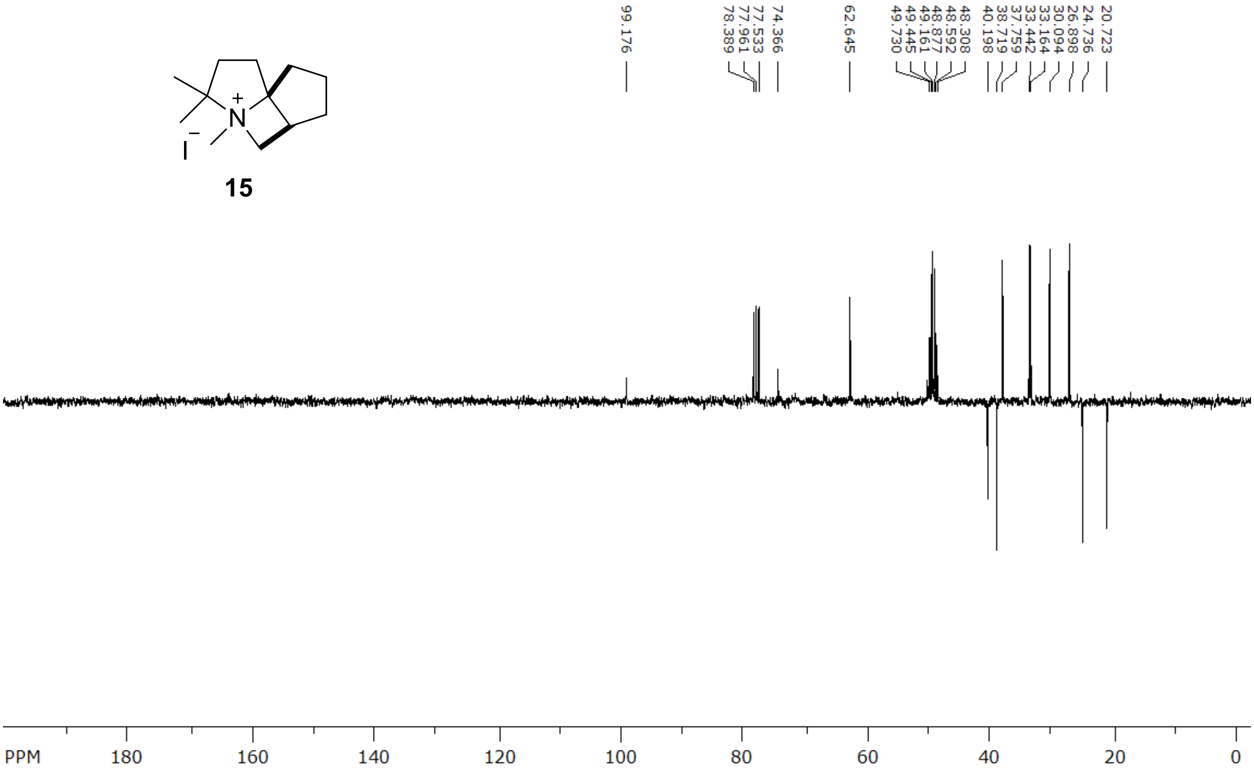

Figure S33. ^{13}C NMR spectrum of **15** in $CDCl_3$-CD_3OD mixture at 75 MHz

3.11 2,3,3-Trimethyl-1,2,3,4,5,7,8,8a-octahydrocyclopenta[*c*]azepine (**16**)

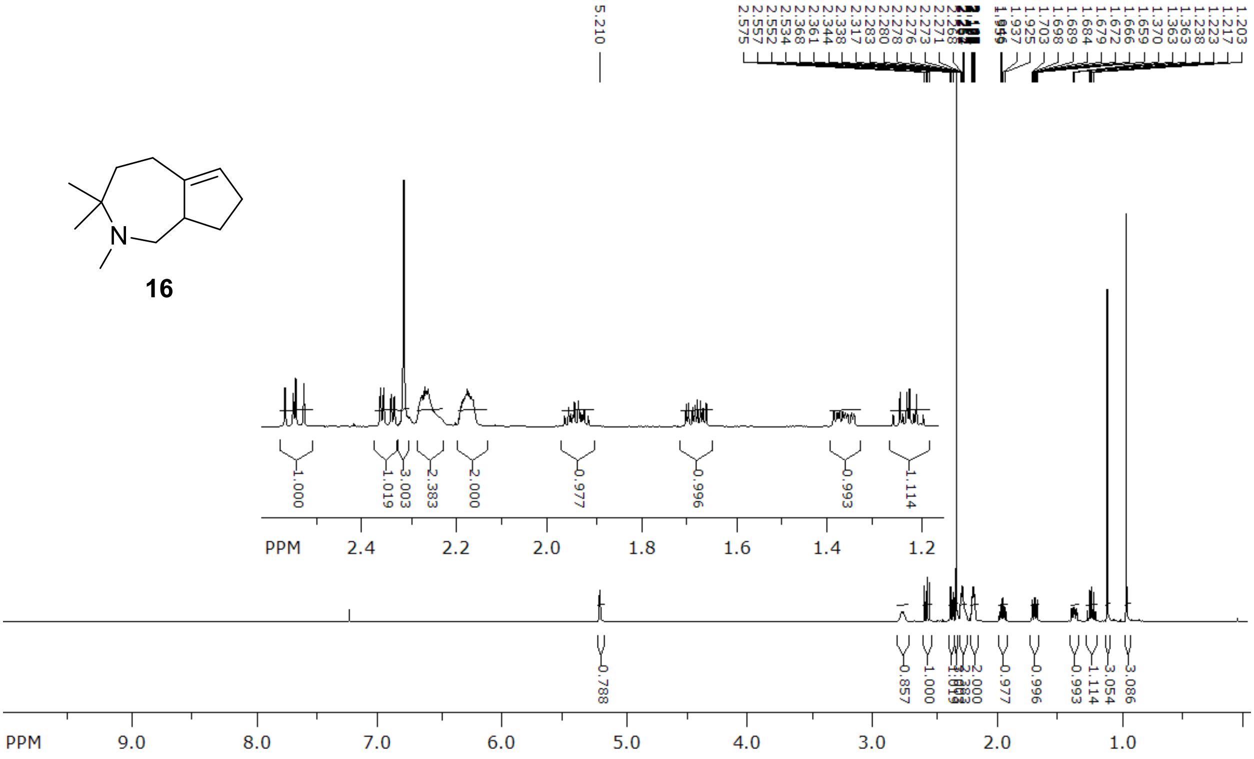

Figure S34. ^{1}H NMR spectrum of **16** in $CDCl_3$ at 600 MHz

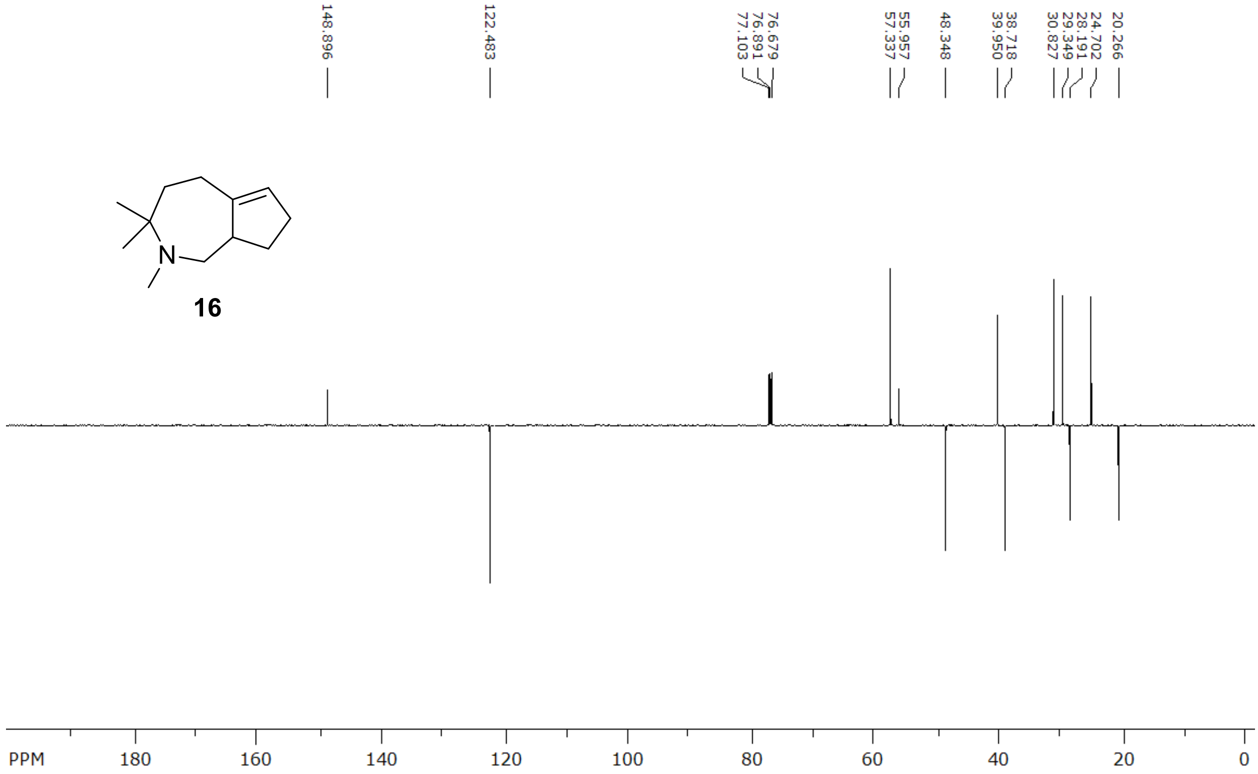

Figure S35. ^{13}C NMR spectrum of **15** in $CDCl_3$ at 150 MHz.

4. 2D NMR spectral data.

4.1 (5a*S*(*R*),8a*R*(*S*))-3,3-Dimethyloctahydrocyclopenta[2,3]azeto[1,2-*a*]pyrrole (**3**)

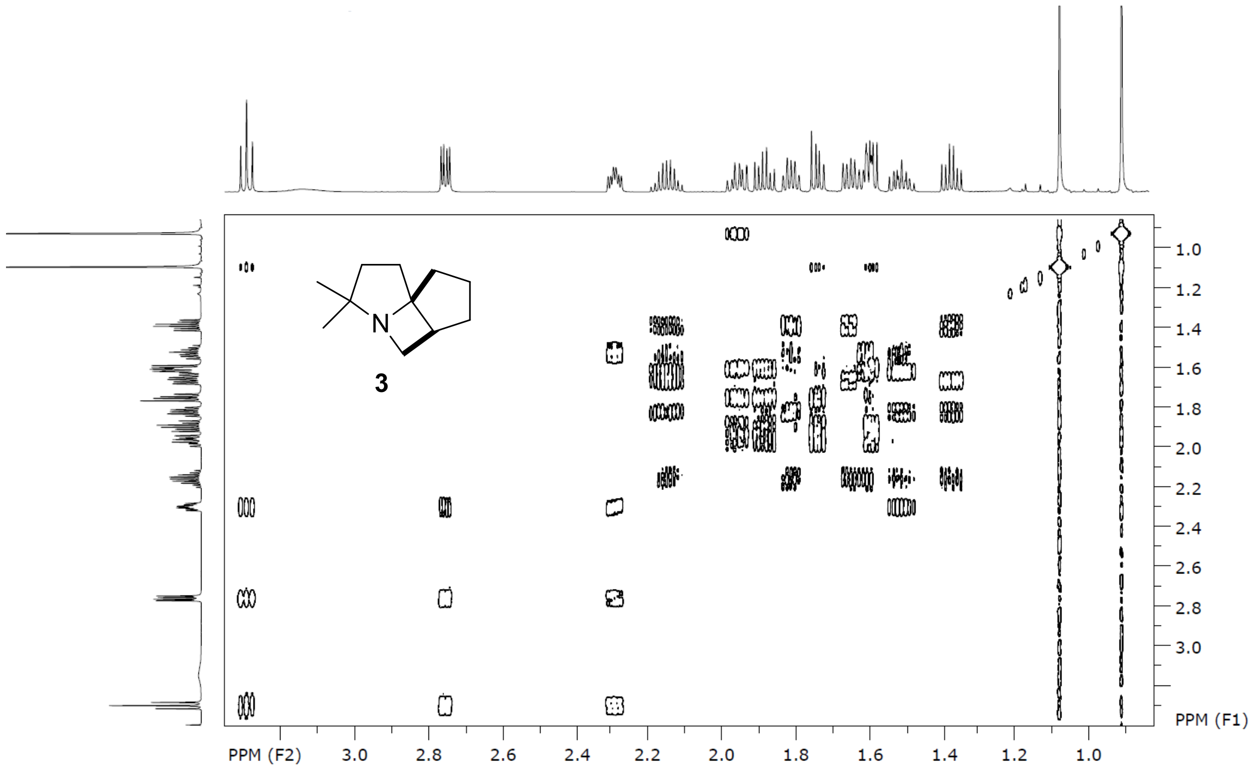

Figure S36. ^{1}H-^{1}H COSY NMR spectrum of **3** in $CDCl_3$.

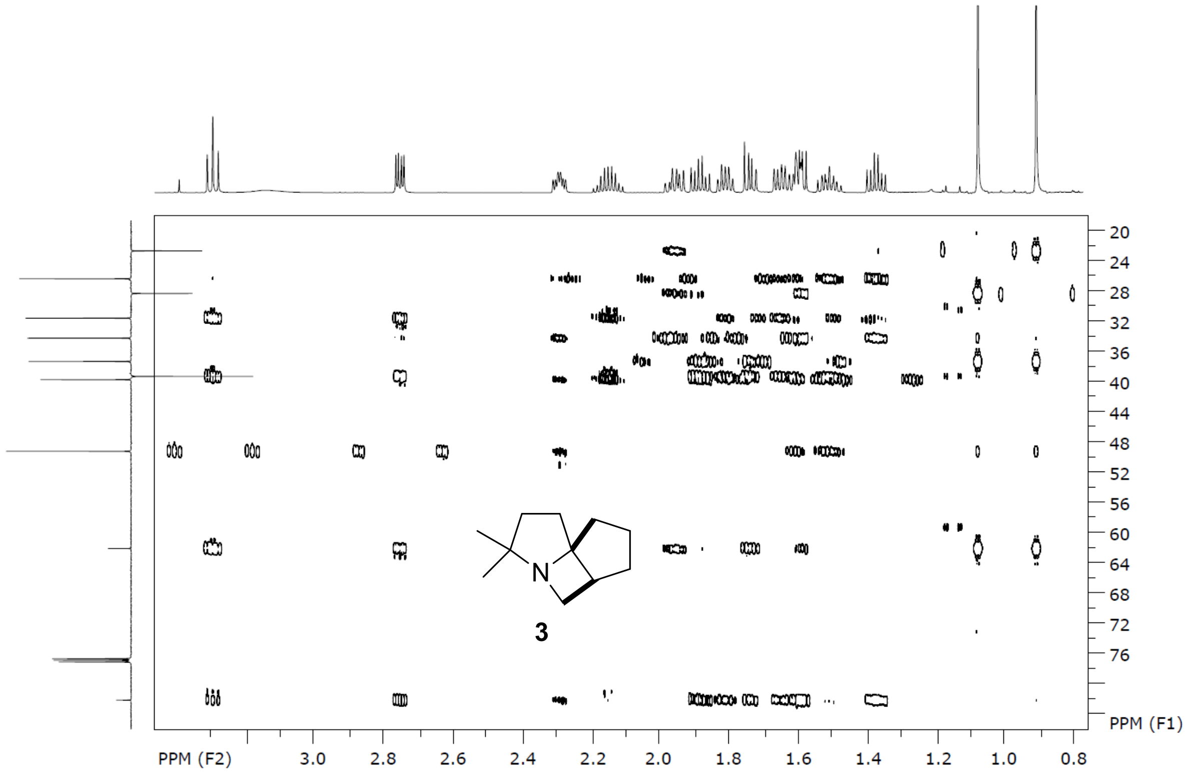

Figure S37. ^{1}H-^{13}C HMBC NMR spectrum of **3** in $CDCl_3$.

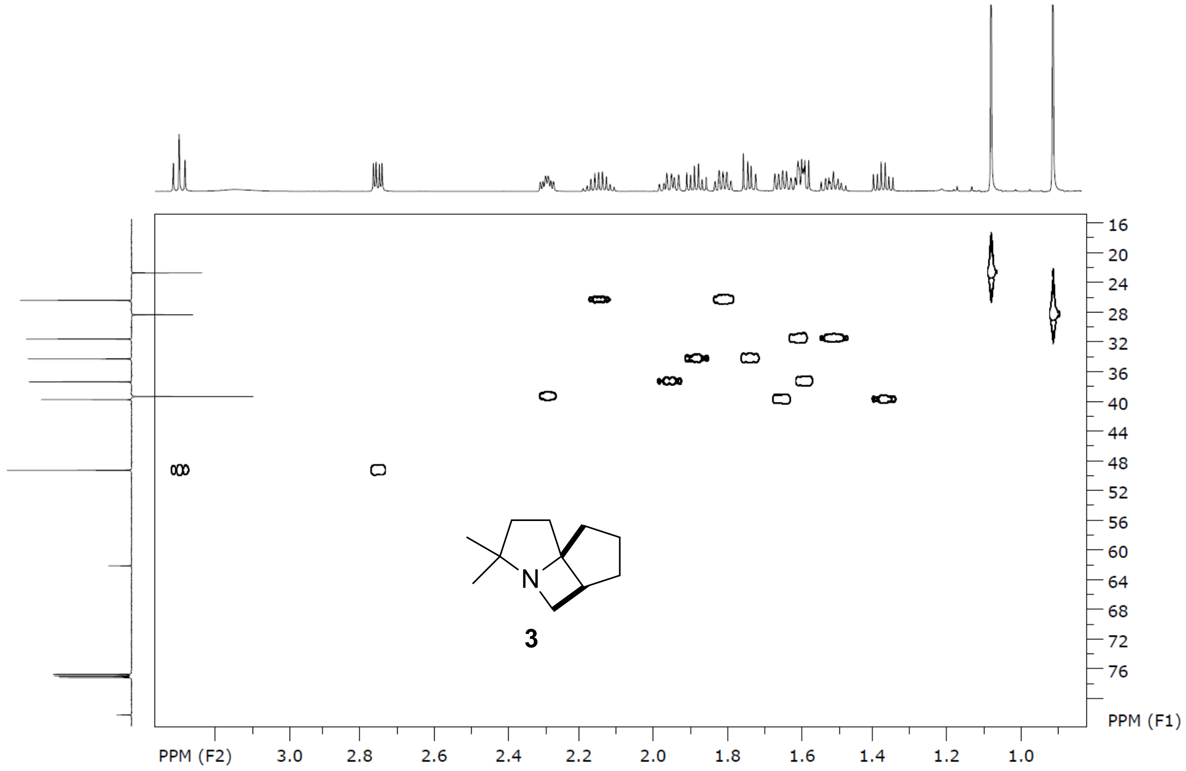

Figure S38. ^{1}H-^{13}C HSQC NMR spectrum of **3** in $CDCl_3$.

4.2 (5a*S*(*R*),8a*R*(*S*))-3,3-Dimethyloctahydro-1*H*-cyclopenta[2,3]azeto[1,2-*a*]pyrrol-4-ium bromide (**3×HBr**)

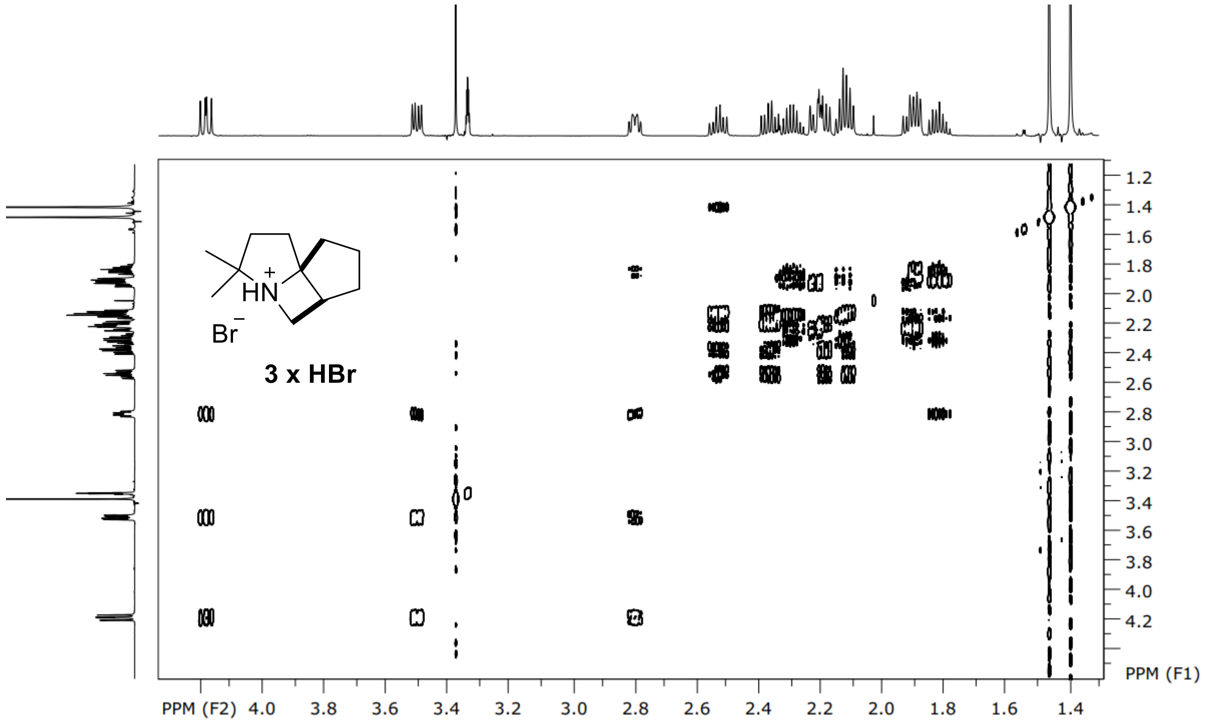

Figure S39. ^{1}H-^{1}H COSY NMR spectrum of **3×HBr** in CD_3OD

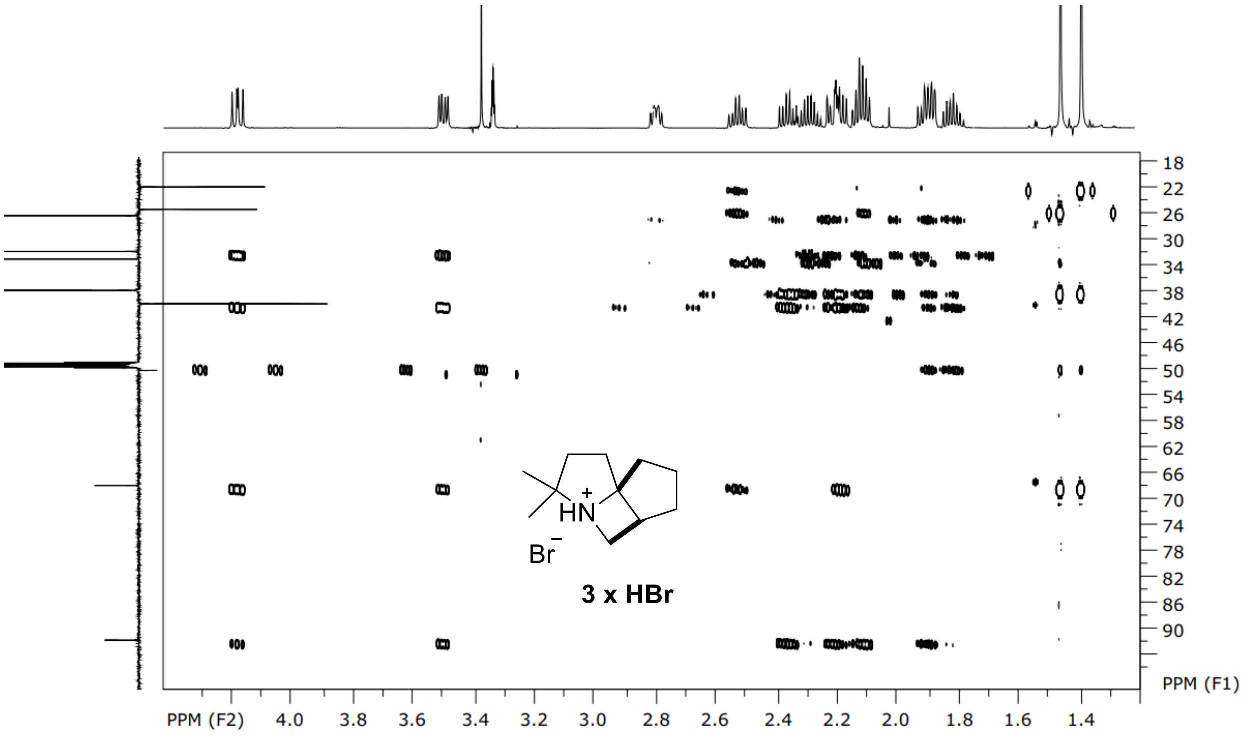

Figure S40. ^{1}H-^{13}C HMBC NMR spectrum of **3×HBr** in CD_3OD

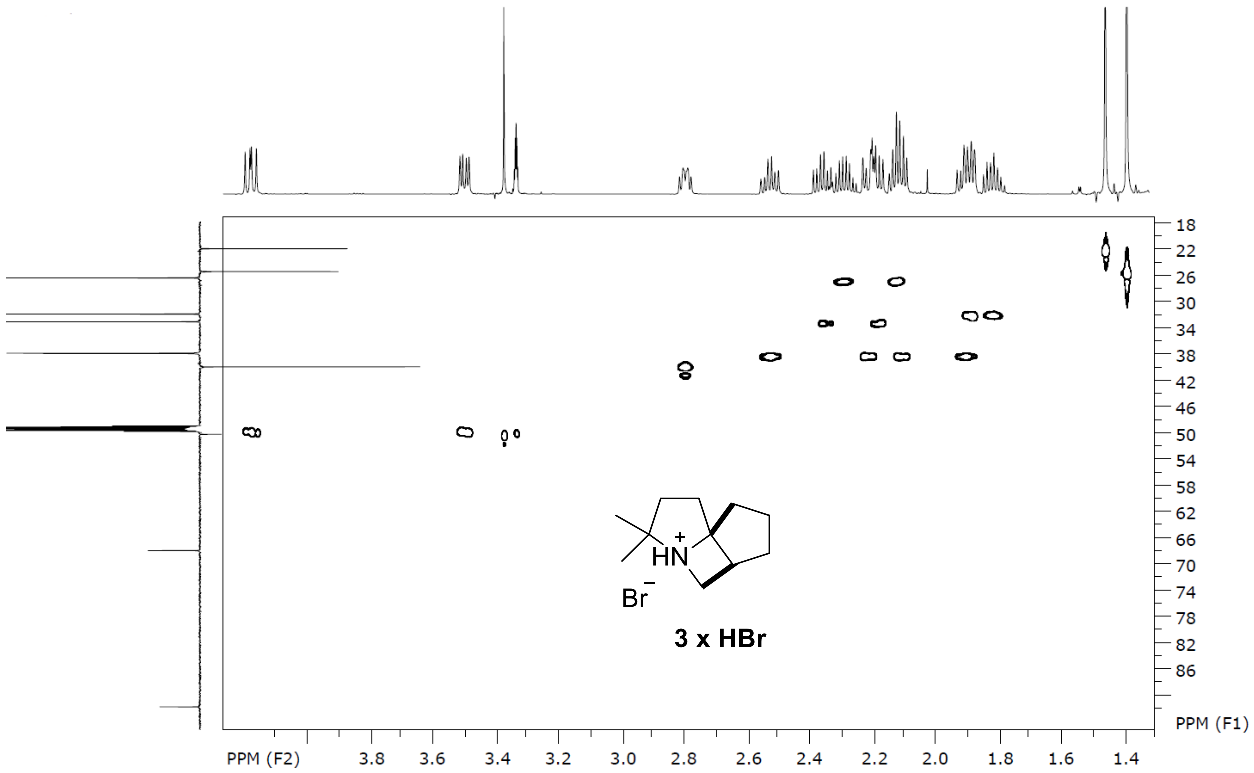

Figure S41. ^{1}H-^{13}C HSQC NMR spectrum of **3×HBr** in CD_3OD

4.3 2-(Benzyloxy)-3,3-dimethyl-1,2,3,4,5,7,8,8a-octahydrocyclopenta[*c*]azepine (**4c**)

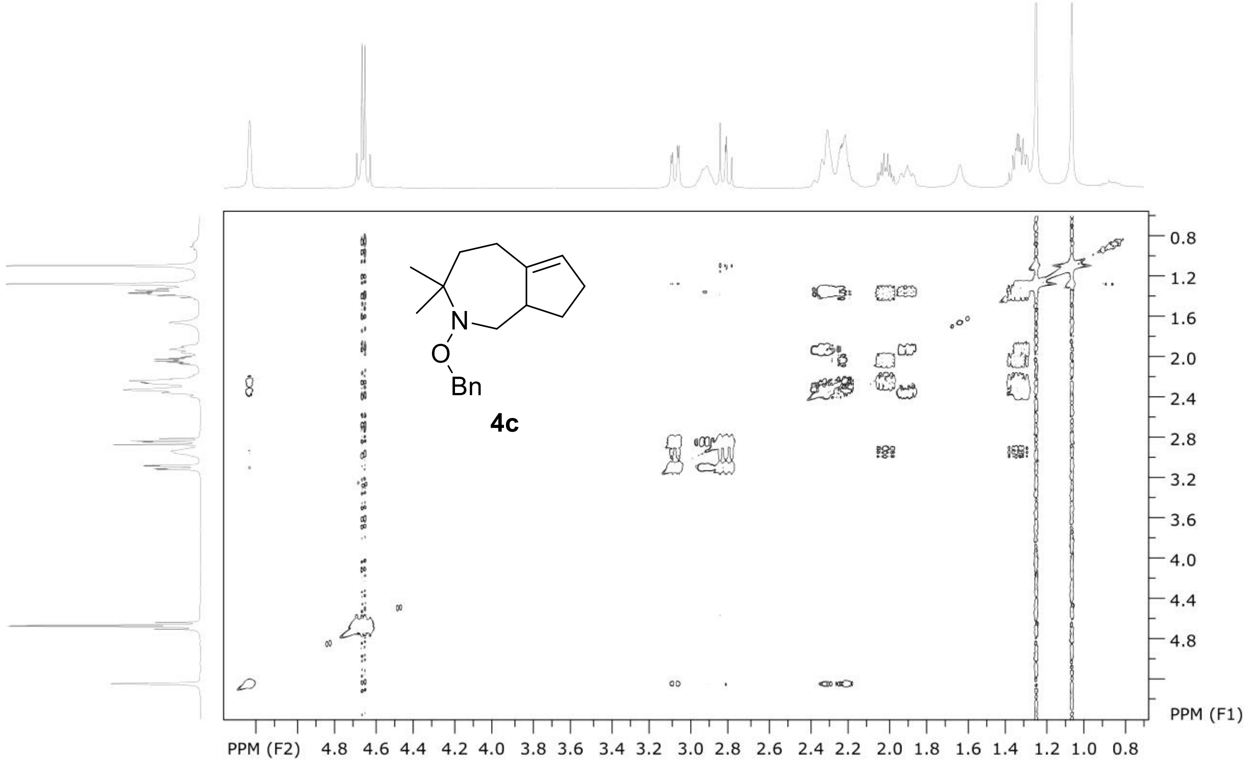

Figure S42. ^{1}H-^{1}H COSY NMR spectrum of **4c** in $CDCl_3$

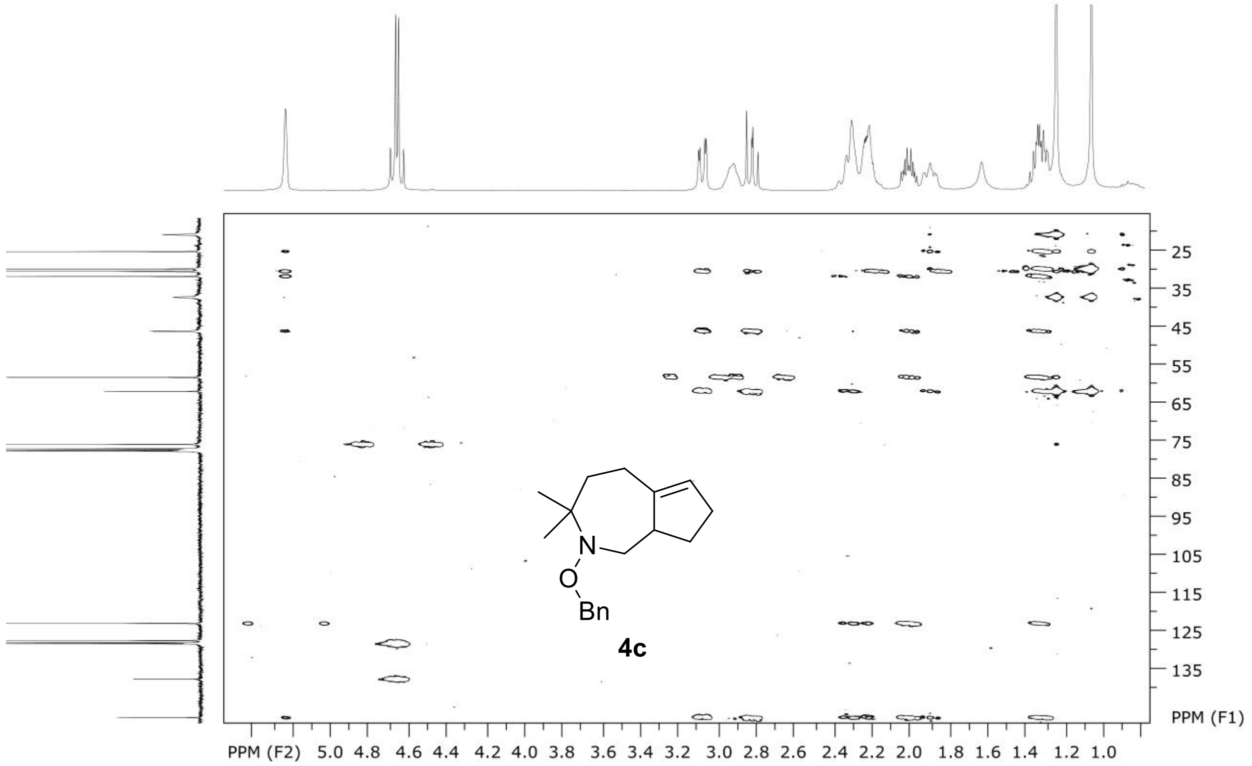

Figure S43. ^{1}H-^{13}C HMBC NMR spectrum of **4c** in $CDCl_3$

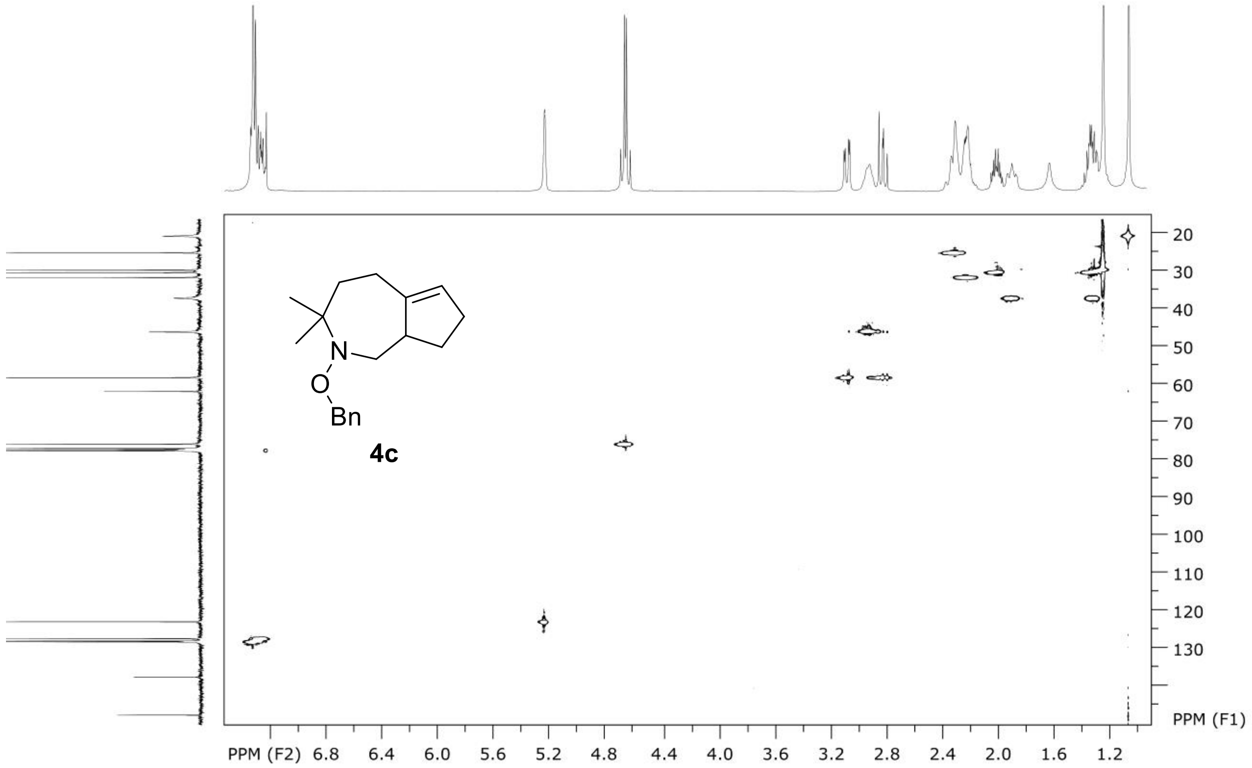

Figure S44. ^{1}H-^{13}C HSQC NMR spectrum of **4c** in $CDCl_3$

4.4 2-(Benzyloxy)-3,3-dimethyl-1,2,3,4,6,7,8,8a-octahydrocyclopenta[*c*]azepine (**5c**)

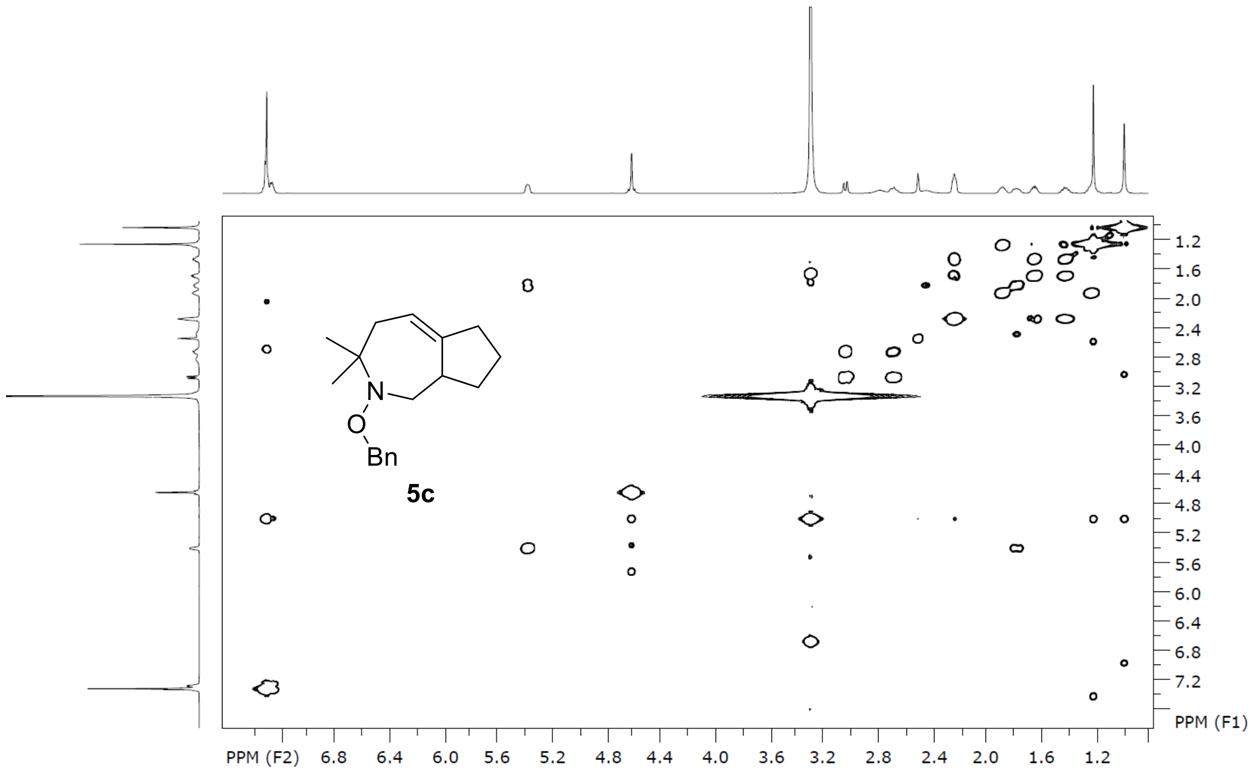

Figure S45. ^{1}H-^{1}H COSY NMR spectrum of **5c** in DMSO-d6 (T=333 K)

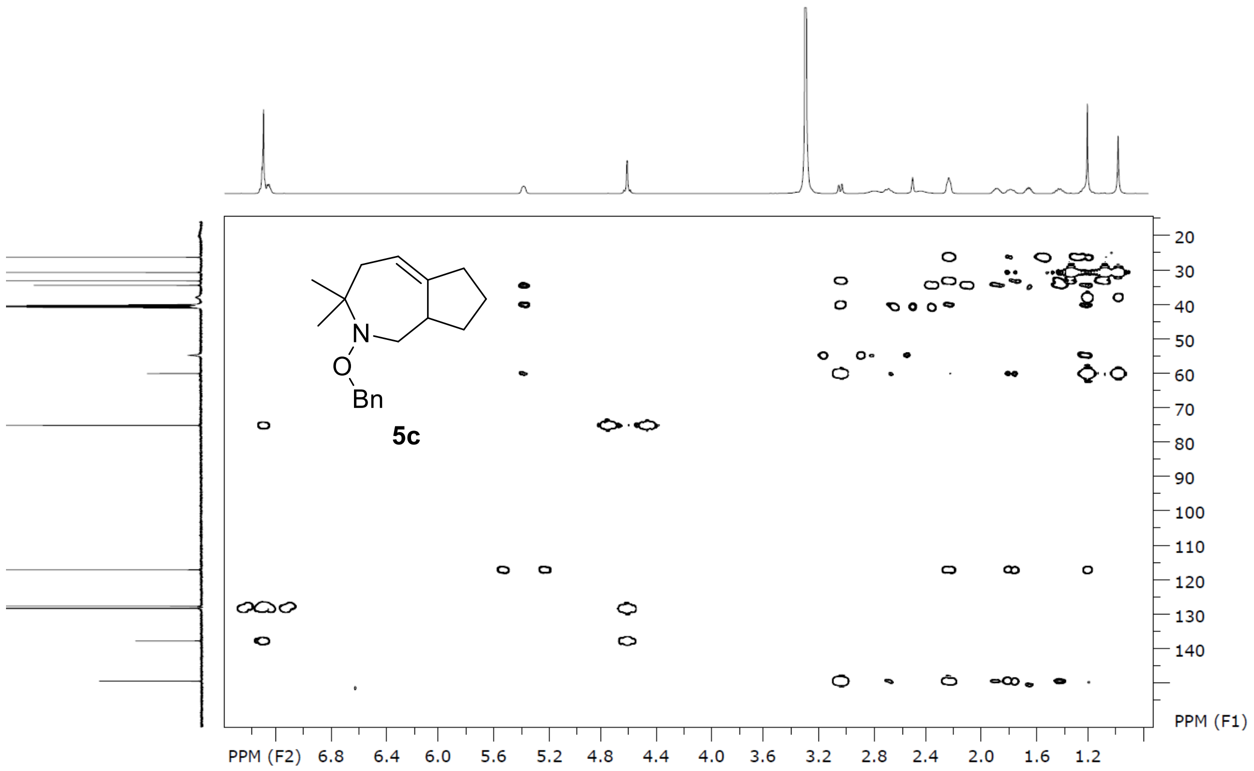

Figure S46. ^{1}H-^{13}C HMBC NMR spectrum of **5c** in DMSO-d6 (T=333 K)

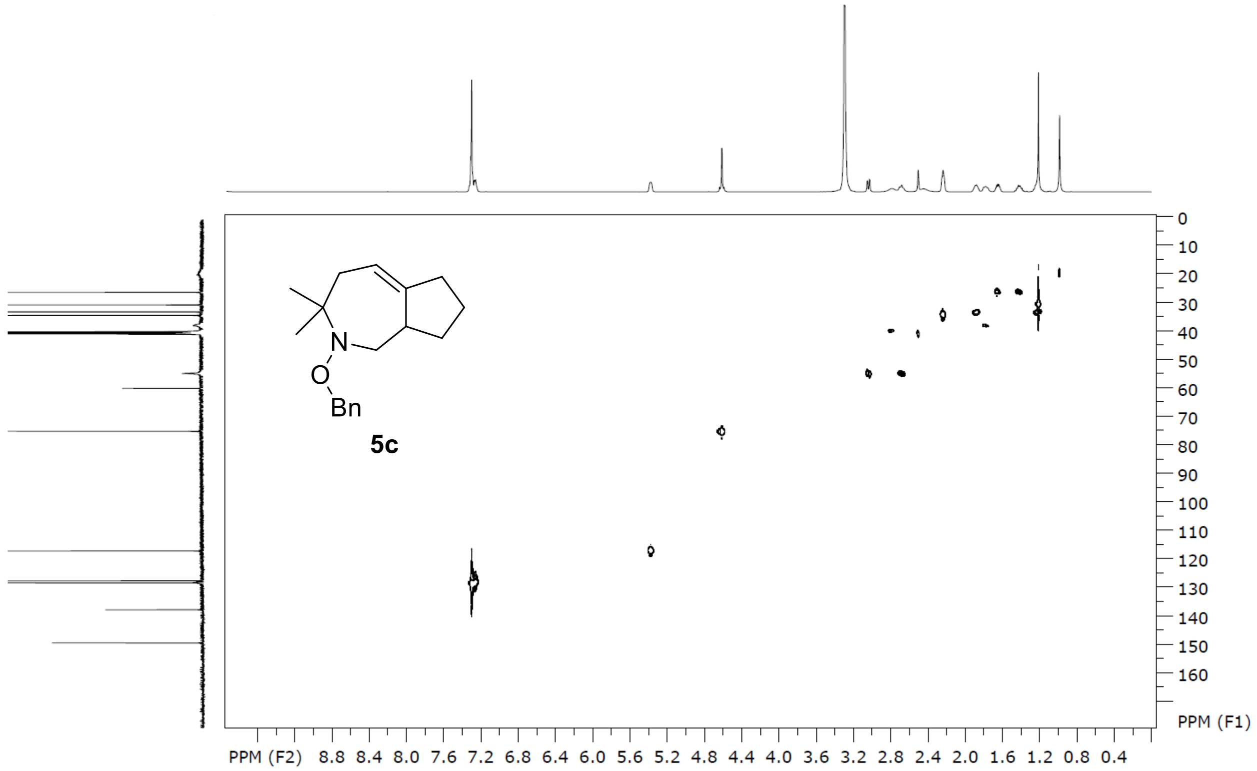

Figure S47. ^{1}H-^{13}C HSQC NMR spectrum of **5c** in DMSO-d$_6$ (T=333 K)

4.5 3,3-Dimethyl-1,4,5,7,8,8*a*-hexahydrocyclopenta[*c*]azepin-2(3*H*)-yl benzoate (**4d**)

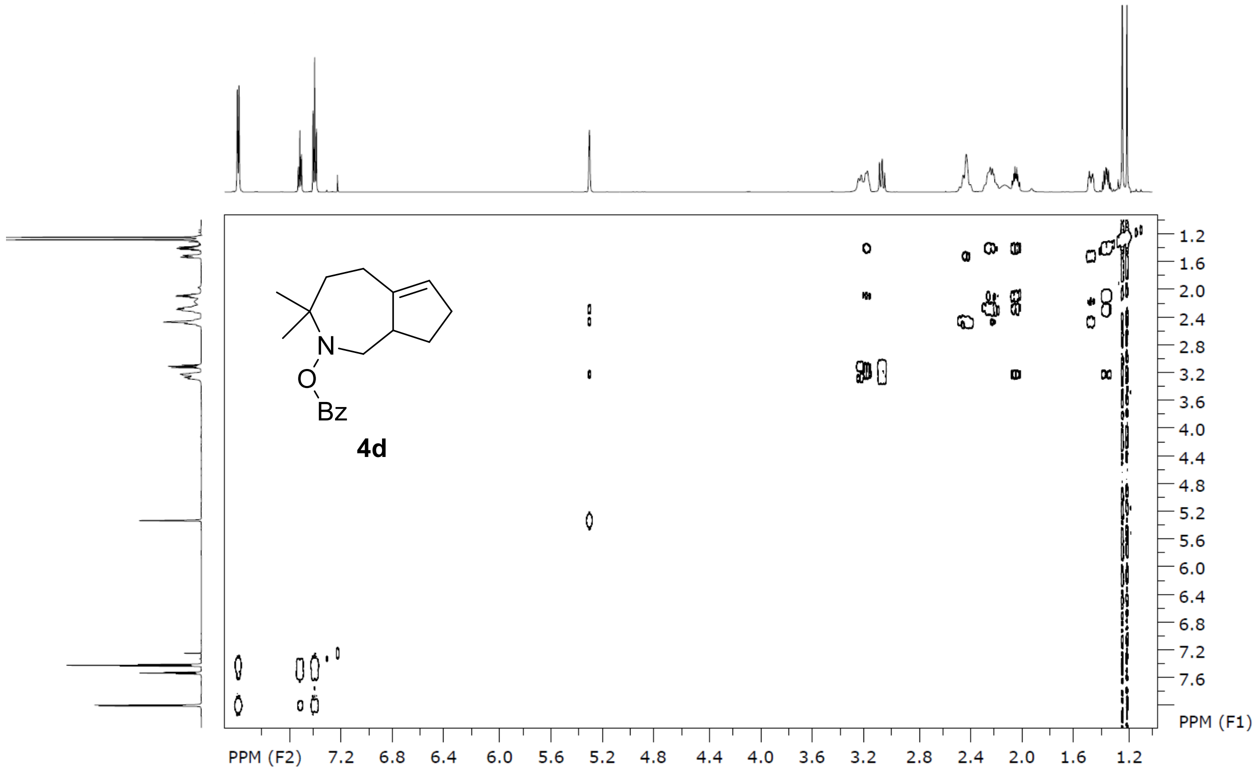

Figure S48. ^{1}H-^{1}H COSY NMR spectrum of **4d** in CDCl$_3$

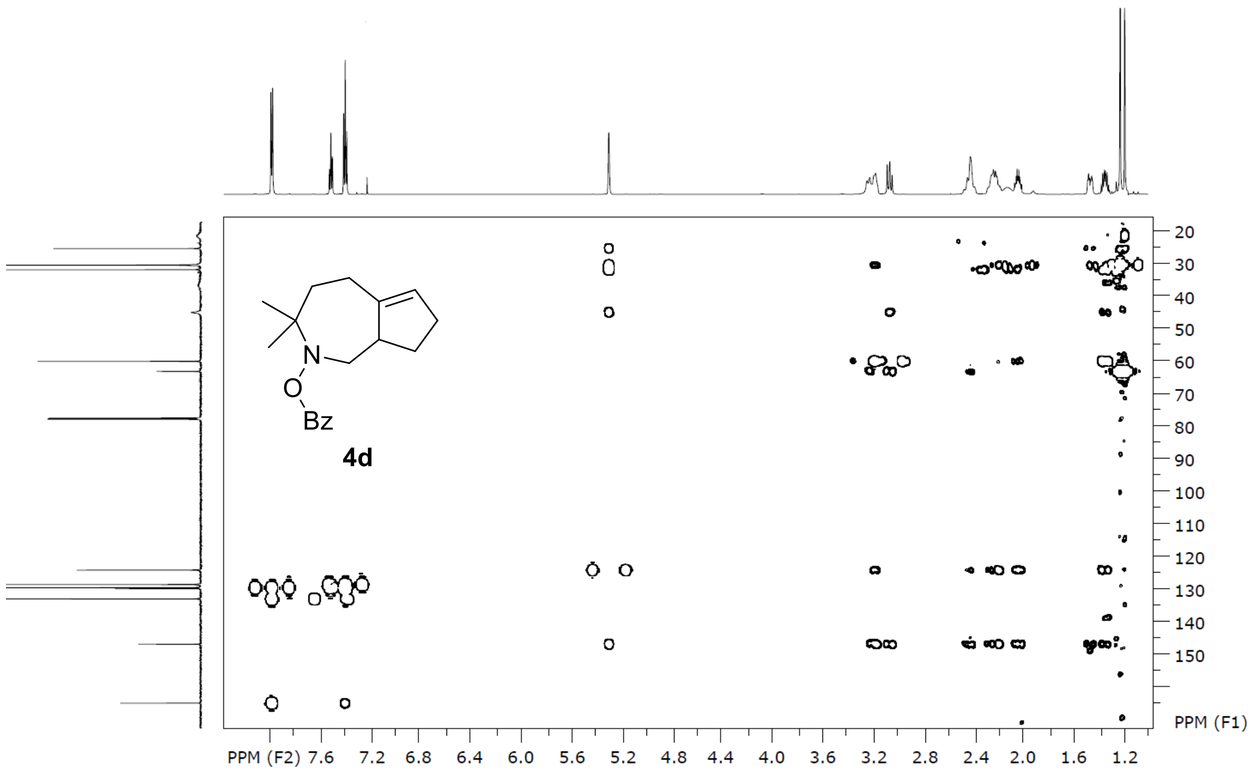

Figure S49. ^1H-^{13}C HMBC NMR spectrum of **4d** in $CDCl_3$

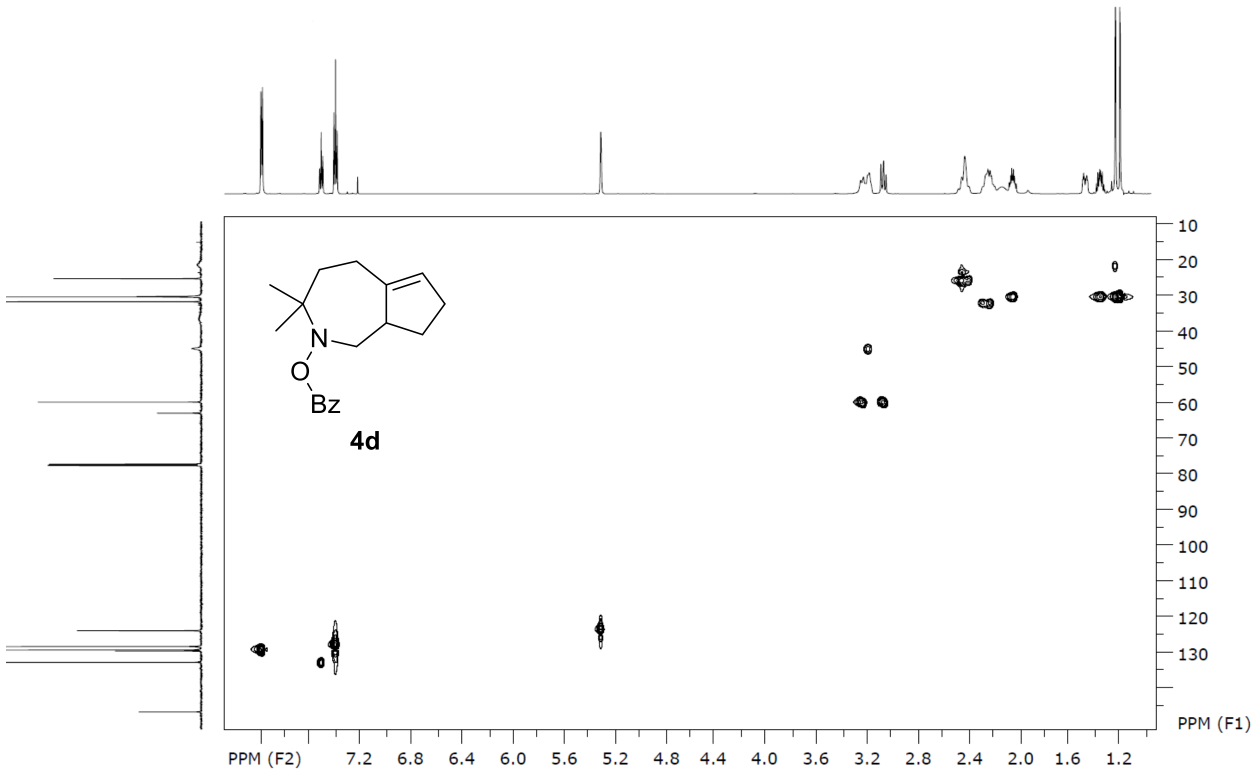

Figure S50. ^1H-^{13}C HSQC NMR spectrum of **4d** in $CDCl_3$

4.6 3,3-Dimethyl-3,4,6,7,8,8*a*-hexahydrocyclopenta[*c*]azepin-2(1*H*)-yl benzoate (**5d**)

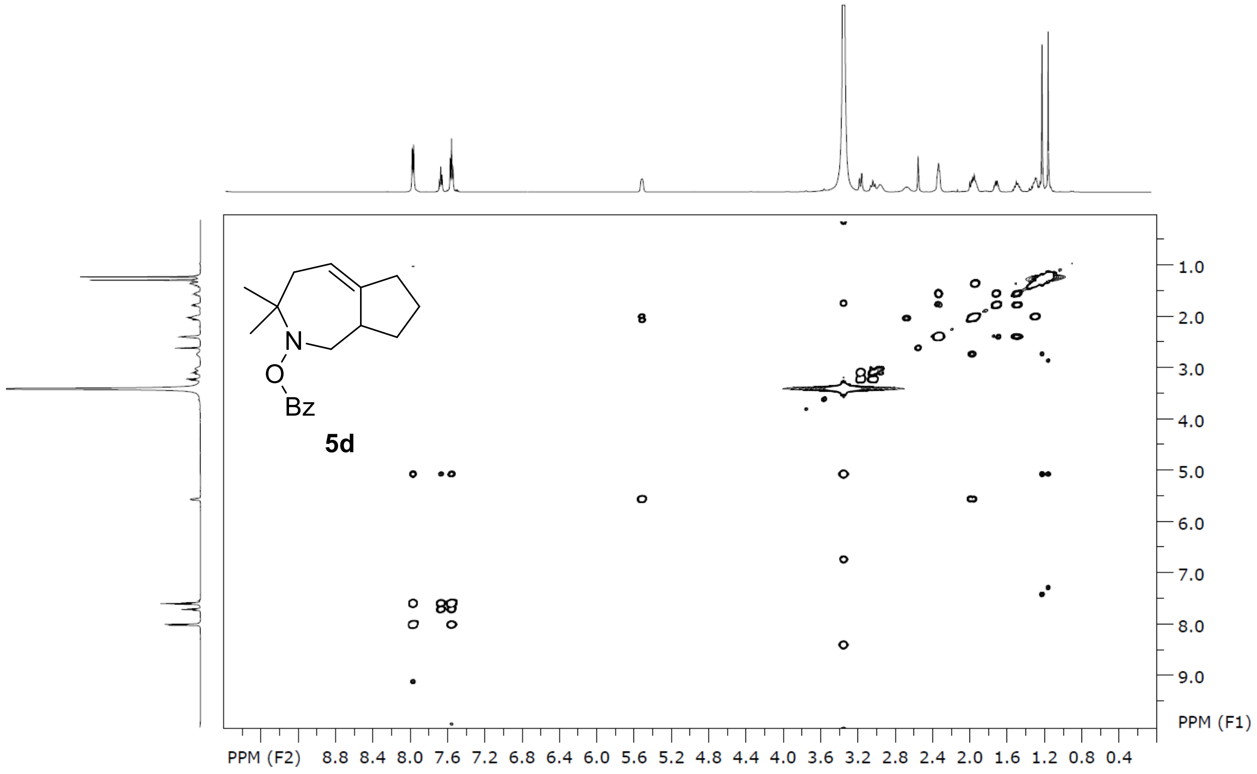

Figure S51. ^{1}H-^{1}H COSY NMR spectrum of **5d** in DMSO-d$_6$ (T=333 K)

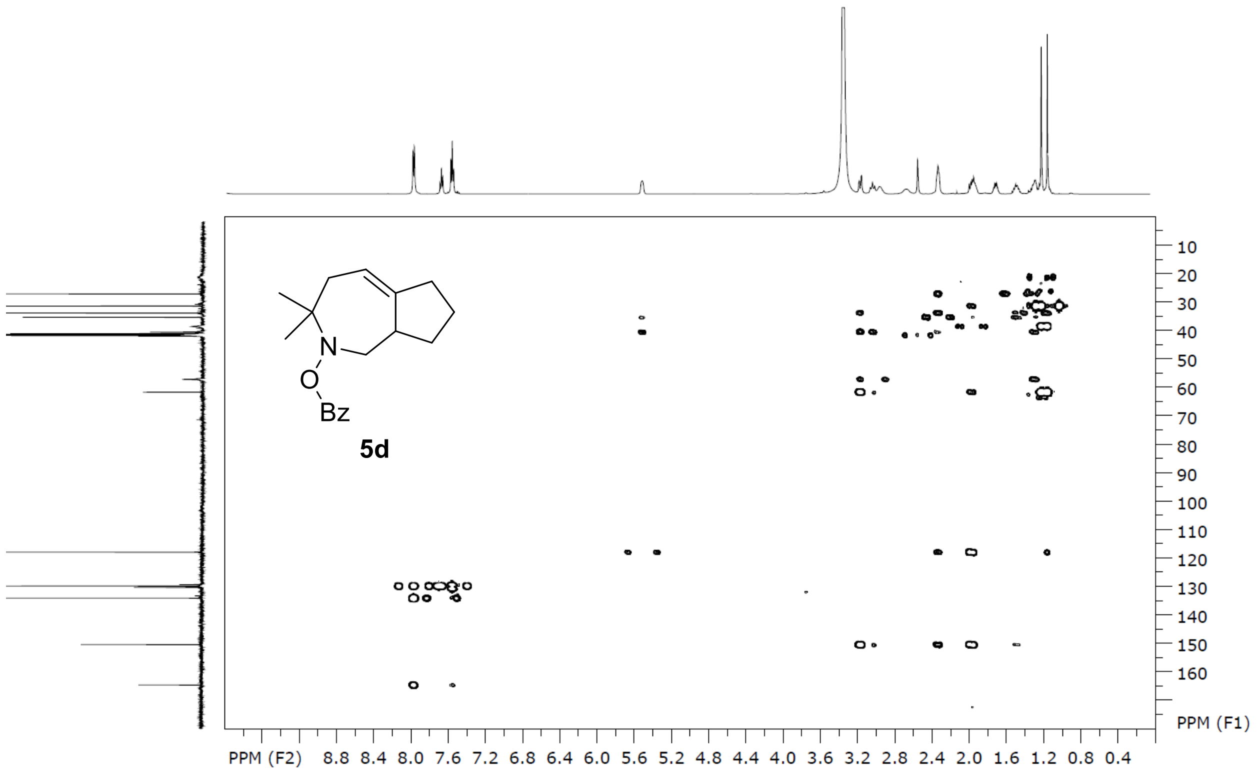

Figure S52. ^{1}H-^{13}C HMBC NMR spectrum of **5d** in DMSO-d$_6$ (T=333 K)

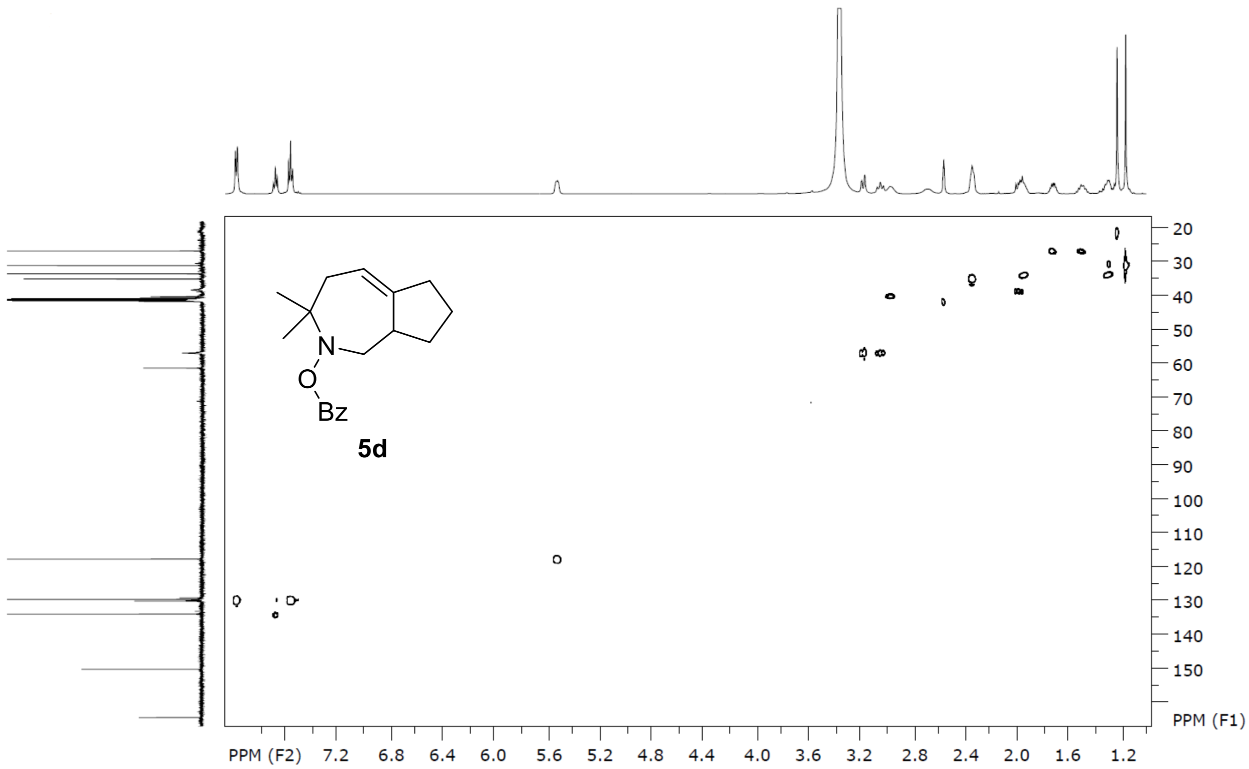

Figure S53. ^{1}H-^{13}C HSQC NMR spectrum of **5d** in DMSO-d_6 (T=333 K)

4.7 2,3,3-Trimethyl-1,2,3,4,5,7,8,8a-octahydrocyclopenta[*c*]azepine (**16**)

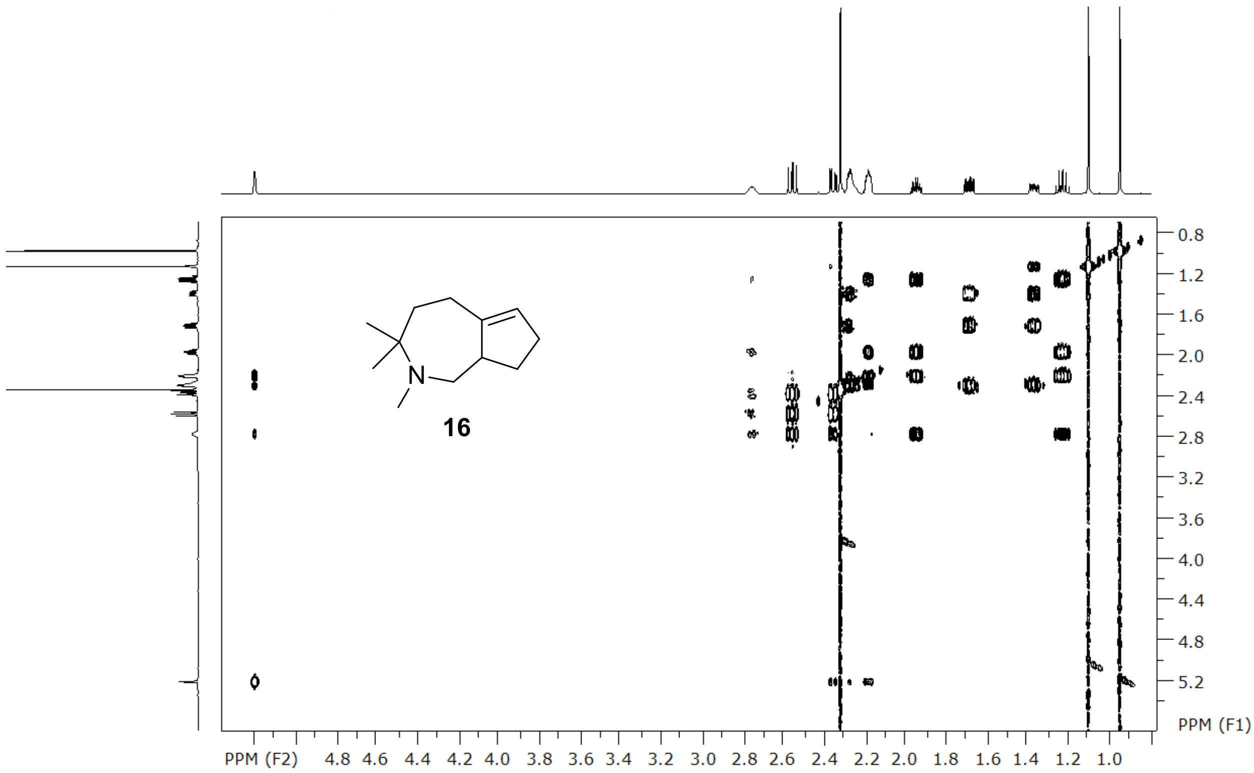

Figure S54. ^{1}H-^{1}H COSY NMR spectrum of **16** in $CDCl_3$

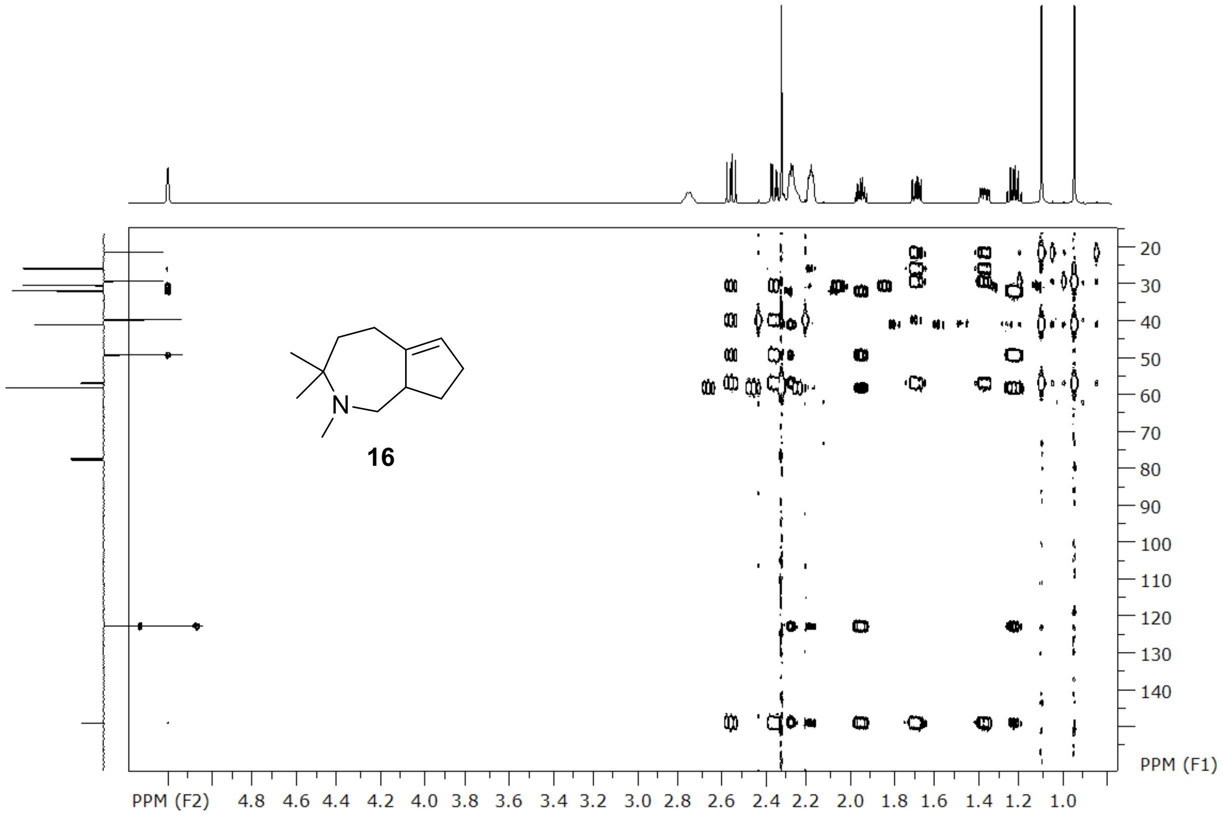

Figure S55. ^{1}H-^{13}C HMBC NMR spectrum of **16** in $CDCl_3$

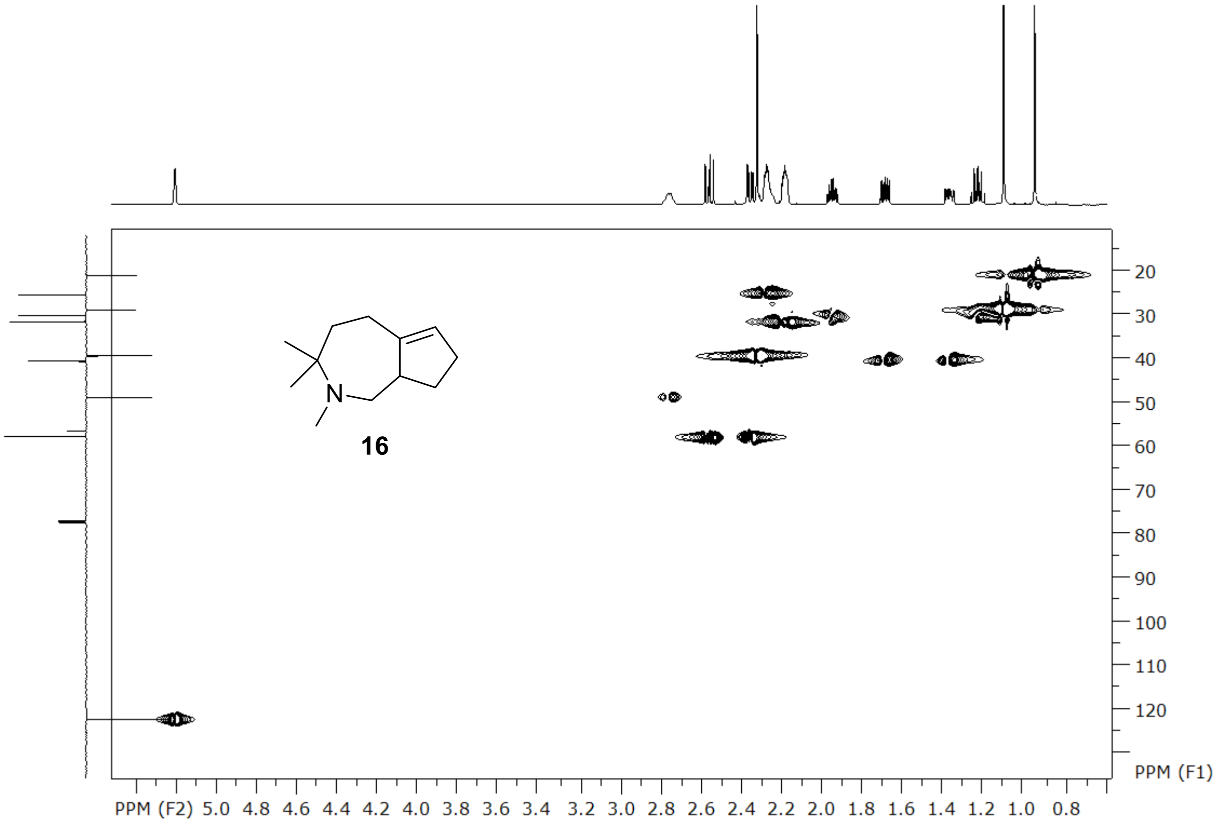

Figure S56. ^{1}H-^{13}C HSQC NMR spectrum of **16** in $CDCl_3$

5. EPR spectral data

5.1 (5*R*(*S*),6*R*(*S*))-6-((Benzoyloxy)methyl)-2,2-dimethyl-1-azaspiro[4.4]nonan-1-oxyl (**13**)

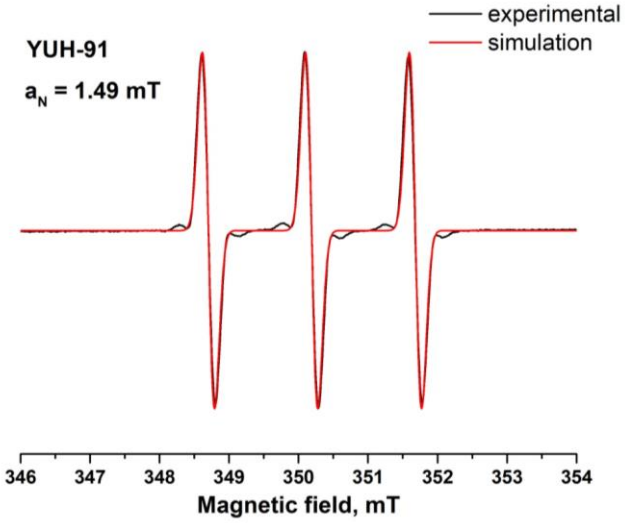

Figure S57. EPR spectrum of **13** in CH_3OH; field sweep, 100 Gs; modulation amplitude, 0.4 Gs; microwave power, 2.0 mW; time constant, 20.48 ms; spectrum scan time, 21.39 s; number of scans 16.

6. Gas chromatography data

6.1 Gas chromatography data of Hofmann elimination of salt **15** (reaction mass)

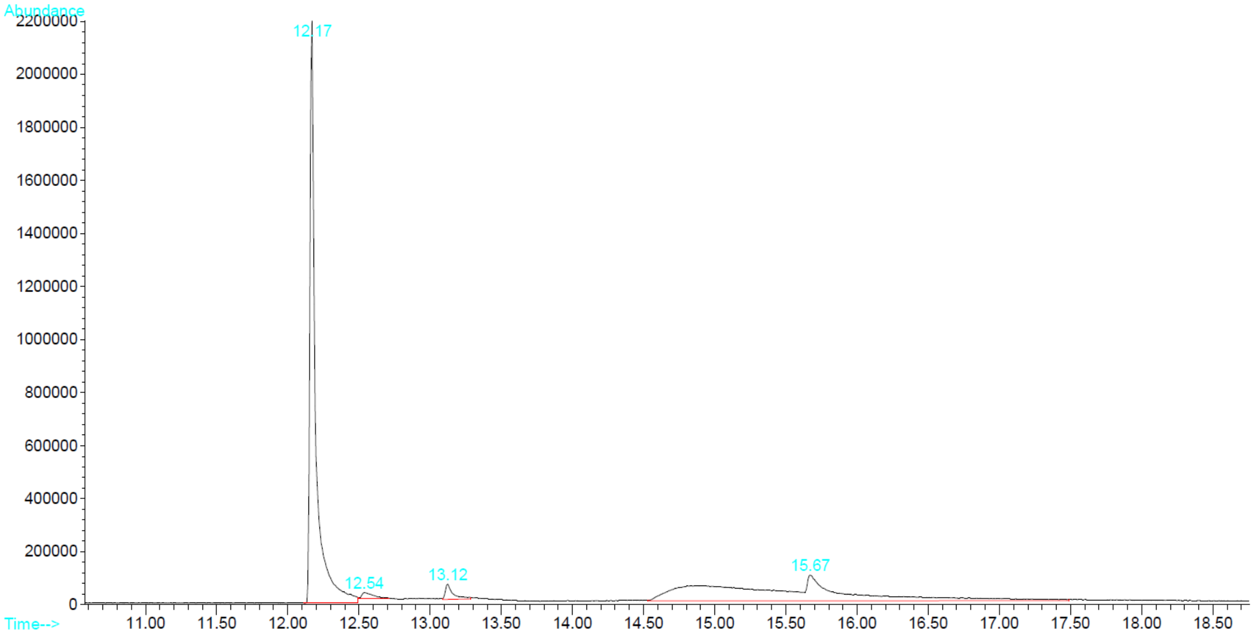

Figure S58. Chromatogram of reaction mass from Hofmann elimination of salt **15**.

Table S1. Total Ion Chromatogram of reaction mass from Hofmann elimination of salt **15**.

Retention Time	Area	Area %	Name
11.816	90170	0.510	MM=165
12.199	14640088	82.760	MM=179
12.516	176244	0.996	MM=179
13.130	110428	0.624	MM=195
13.253	90884	0.514	MM=177
13.837	40827	0.231	MM=225
15.729	2541191	14.365	MM=197

7. X-ray analysis data for compound **3×HBr**

7.1 Experimental details for compound **3×HBr**

Crystallographic data for salt **3×HBr**: $C_{11}H_{20}N^{+}Br^{-}$, *M* 246.19, orthorhombic, *Pna2₁*, *a* 13.5147(5), *b* 8.8425(4), *c* 10.0361(3) Å, *V* 1199.35(8) Å^3, *Z* 4, D_{calcd} 1.363,g·cm^{-3}, μ(Mo-$K\alpha$) 3.388 mm^{-1}, F(000) 512, (θ 3.07 – 26.42°, completeness 98.5%), T 296(2) K, colorless plate, crystal size (0.80 × 0.50 × 0.18) mm^3, transmission 0.0900 − 0.1495, 18018 measured reflections in index range -16<=h<=16, -11<=k<=11, -12<=l<=12, 2394 independent (R_{int} 0.0270), 121 parameters, 11 restraints, R_1 0.0376 (for 1979 observed $I>2\sigma(I)$), wR_2 0.0962 (all data), GOOF 1.069, largest diff. peak and hole 0.70 and -0.44 e.A^{-3}.

7.2 The structure and atom numbering of **3×HBr**

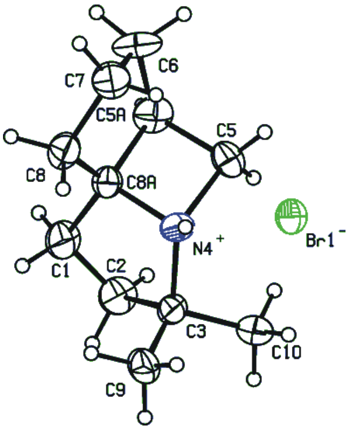

Figure S59. The structure and atom numbering of **3×HBr** (The thermal ellipsoids are drawn at the 30% probability level).

8. NMR spectrum fine structure analysis

8.1 (5a*S*(*R*),8a*R*(*S*))-3,3-Dimethyloctahydro-1*H*-cyclopenta[2,3]azeto[1,2-*a*]pyrrol-4-ium bromide (**3×HBr**)

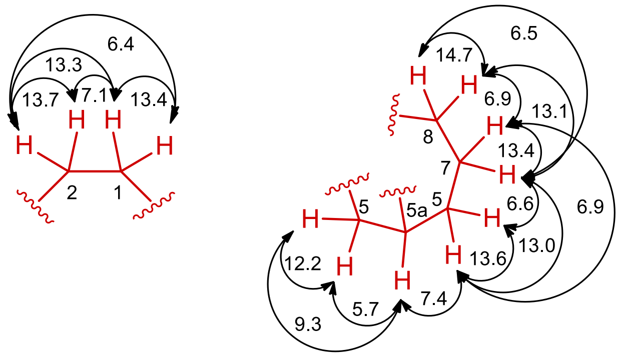

Figure S60. NMR spectrum fine structure analysis of **3×HBr**

8.2 Line shape analysis of multiplets for **3×HBr**

Line shape analysis of multiplets for **3×HBr** was performed using the gNMR 5.0 software is shown in **Figure S61**. Parameters of spin system are shown in **Table S2**.

[Budzelaar, P. H. M. "gNMR, version 5.0. 6.0." *Ivorysoft, Nijmegen, Netherlands* (2006).]

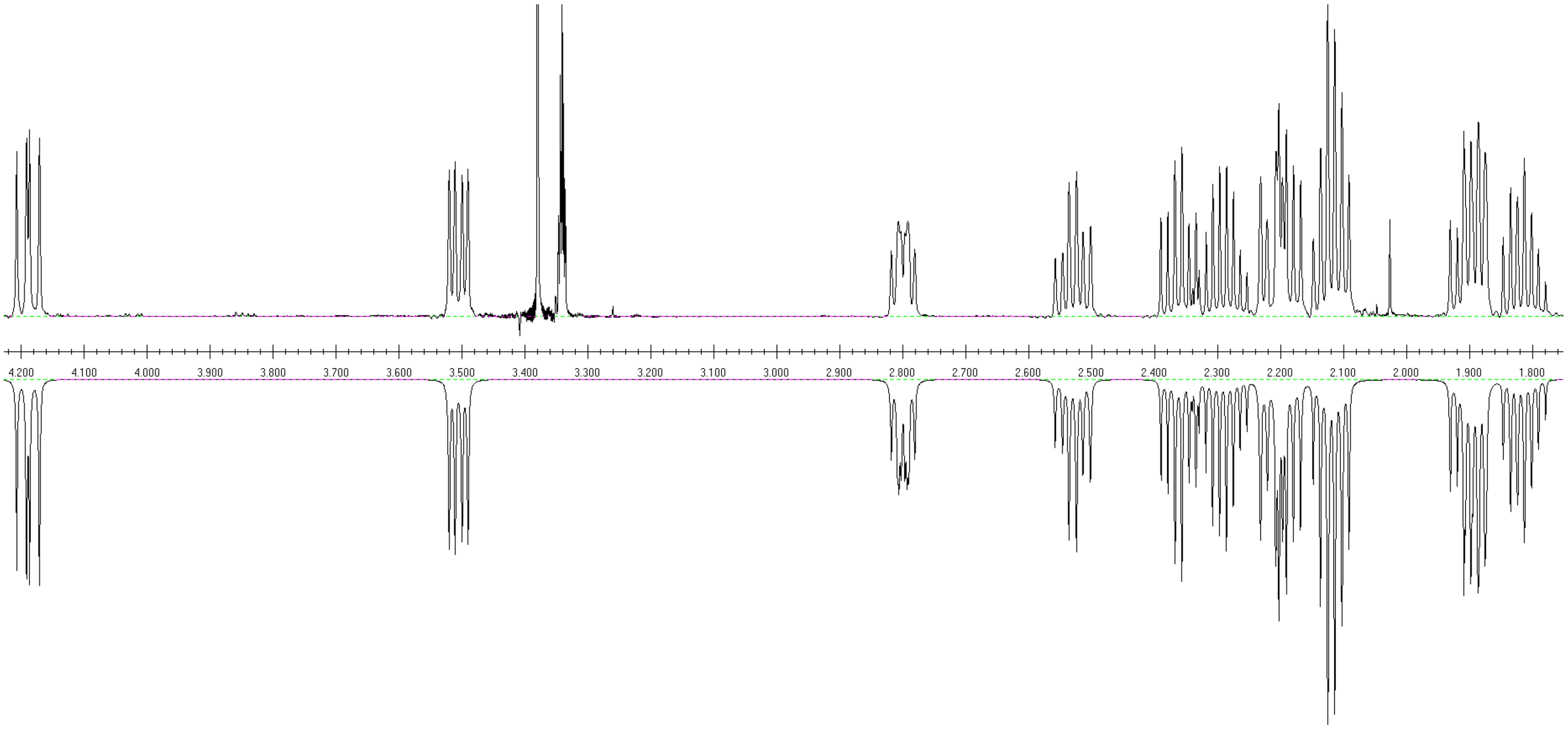

Figure S61. Line shape analysis of multiplets for **3×HBr**

Table S2. Parameters of spin system for **3×HBr**.

№	Nucleus	n	Shift	Width	J[1]	J[2]	J[3]	J[4]	J[5]	J[6]	J[7]	J[8]	J[9]	J[10]	J[11]	J[12]
1	1H	1	4,188	1,40												
2	1H	1	3,505	1,71	-12,25											
3	1H	1	2,799	1,83	9,30	5,67										
4	1H	1	2,529	1,71	0,00	0,00	0,00									
5	1H	1	2,362	1,30	0,00	0,00	0,00	13,26								
6	1H	1	2,295	1,11	0,00	0,00	0,00	0,00	0,00							
7	1H	1	2,214	1,95	0,00	0,00	0,00	0,00	0,00	6,55						
8	1H	1	2,186	1,56	0,00	0,00	0,00	7,09	-13,74	0,00	0,00					
9	1H	1	2,126	1,41	0,00	0,00	0,00	0,00	0,00	-13,44	0,00	0,00				
10	1H	1	2,108	1,55	0,00	0,00	0,00	13,35	6,41	0,00	0,00	0,00	0,00			
11	1H	1	1,903	1,47	0,00	0,00	0,00	0,00	0,00	13,12	-14,76	0,00	6,86	0,00		
12	1H	1	1,890	2,15	0,00	0,00	0,00	0,00	0,00	6,58	0,00	0,00	0,00	0,00	0,00	
13	1H	1	1,815	1,18	0,00	0,00	7,43	0,00	0,00	12,97	0,00	0,00	6,89	0,00	0,00	-13,57

9. HPLC analysis

9.1 HPLC analysis of 2-(benzyloxy)-3,3-dimethyl-1,2,3,4,5,7,8,8a-octahydrocyclopenta[*c*]azepine (**4c**) and 2-(benzyloxy)-3,3-dimethyl-1,2,3,4,5,6,7,8-octahydrocyclopenta[*c*]azepine (**6c**) mixture and pure **4c** fraction.

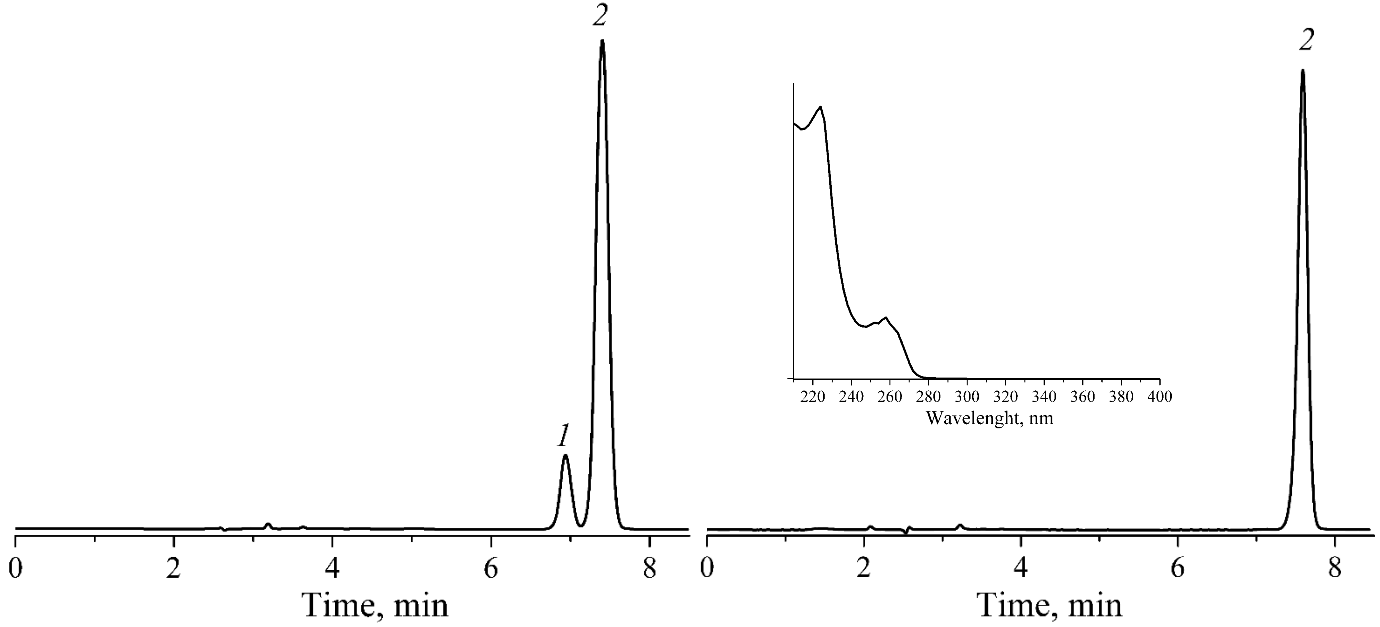

Figure S62. The HPLC-UV analysis of **4c** and **6c** mixture (left) and pure **4c** fraction (right); column: Zorbax C8 (250 mm × 4.6 mm, i.d., 5µm); mobile phase: acetonitrile/water (8:2 v/v); flow rate: 1.0 mL/min; wavelength: 260 nm; temperature: 35 °C; sampling volume: 20 µL; insert: UV spectrum of **4c**.
